# RFC2 promotes aerobic glycolysis and progression of colorectal cancer

**DOI:** 10.1186/s12876-023-02984-0

**Published:** 2023-10-11

**Authors:** Fuchen Lou, Mingbao Zhang

**Affiliations:** 1 Department of Endocrinology, The Second Hospital of Shandong University, Jinan, Shandong 250033 P.R. China; 2 Department of Gastroenterology, The Second Hospital of Shandong University, Beiyuan Street 247,Tianqiao District, Jinan, Shandong 250033 P.R. China

**Keywords:** Colorectal cancer, RFC2, CREB5, Aerobic glycolysis, MET/PI3K/AKT/mTOR

## Abstract

**Background:**

Replication factor C subunit 2 (RFC2) participates in the growth and metastasis of various malignancies. Our study investigated the roles of RFC2 in colorectal cancer (CRC).

**Results:**

RFC2 expression was upregulated in CRC tissues and cells. High RFC2 expression was associated with poor prognosis. Knockdown RFC2 inhibited proliferation, induced apoptosis, and suppressed migration and invasion of CRC cells. CREB5 was a transcription factor of RFC2, and CREB5 knockdown suppressed RFC2 expression. Furthermore, RFC2 promoted aerobic glycolysis and MET/PI3K/AKT/mTOR pathway.

**Conclusion:**

RFC2 promoted the progression of CRC cells via activating aerobic glycolysis and the MET/PI3K/AKT/mTOR pathway.

**Supplementary Information:**

The online version contains supplementary material available at 10.1186/s12876-023-02984-0.

## Introduction

Colorectal cancer (CRC) is a leading cause of cancer-related death worldwide [1]. China accounts for 28.8% of the world’s diagnosed cases of CRC in 2020 [2]. CRC screening has been widely used in a large number of countries [3]. However, the participation rate of colonoscopy screening remains low in China [4]. A study showed that CRC cases with distant metastasis account for 25% [5]. Great progress has been made in surgical technique and adjuvant therapy, however, the five-year survival rate is not satisfactory in CRC patients with distant metastasis [6–8]. Therefore, investigating effective biomarkers and therapeutics of CRC is conducive to better treatment of CRC.

Replicate factor C (RFC) is a primer recognition factor of DNA polymerase, which is reported to participate in DNA replication and repair [9]. The RFC family possess five subunits (RFC1-5), those aberrantly expressed in a variety of malignant tumors [10]. RFC3 induces EMT, invasion, and migration of lung adenocarcinoma cells though the Wnt/β-catenin pathway [11]. RFC4 promotes metastasis and stemness by activating Notch1 signaling in non-small-cell lung cancer [12]. RFC5 might be an oncogene and prognostic biomarker of lung cancer [13]. RFC3 mutation and loss of RFC3 expression occur in large fractions of gastric cancer and CRC [14]. RFC4 is frequently overexpressed in CRC, and is associated with tumor progression and worse survival outcome [15]. RFC4 is identified as a radioresistance factor that promotes NHEJ-mediated DNA repair in CRC cells [16]. circ_0038985/miR-3614-5p RFC5 promotes the progression of CRC [17]. RFC2 promotes diffuse lower-grade gliomas progression and correlates with the immune infiltration of lower-grade gliomas [18]. RFC2 plays an oncogenic role in progression of hepatocellular carcinoma by regulating DNA replication and cell cycle [19]. RFC2 promotes cell cycle arrest and its expression is inhibited by miR-744 in CRC [20]. RFC2 is up-regulated in nasopharyngeal carcinoma and might be a candidate molecular marker [21]. Nonetheless, the underline mechanism of RFC2 in CRC is not fully investigated.

CREB5, a transcription factor (TF), interacts with the promoter of genes, and promotes the progression of cancers [22, 23]. Our previous research has shown that CREB5 is increased in CRC tissues and cells, and lncRNA SNHG5/miR-132-3p/CREB5 makes for malignant development of CRC cells [24]. However, the regulatory mechanism of CREB5 in CRC is still unclear.

In this study, we explored the expression and prognostic value of RFC2 in CRC. We sought to investigate the underlying mechanisms of RFC2 in CRC.

## Material and methods

### Bioinformatics analysis

Two hundred seventy-five colon adenocarcinoma and 349 normal tissues were collected from TCGA-COAD dataset (http://gepia2.cancer-pku.cn/#index). The survival analysis of RFC2 expression in patients with CRC was analyzed using Kmplot (http://kmplot.com/analysis/). RFC2 expression was performed gene set enrichment analysis (GSEA) using R package.

### Tissue collection and IHC

Eight pairs of cancer and matched normal tissues were collected from patients at The Second Hospital of Shandong University from January 2021 to December 2021. 50 CRC tumor tissues were collected at The Second Hospital of Shandong University from January 2016 to December 2021. The protocol of this research has been approved by the Ethics Committee of The Second Hospital of Shandong University. Written informed consent was obtained from all patients. The sections of tissues were incubated with an RFC2 antibody (Abcam, 1:200, ab251796).

### Cell culture

Normal human colonic epithelial cells (FHC) and five human CRC cell lines (HCT116, LoVo, SW480, Caco-2, and DLD-1) were purchased from Nanjing Cobioer Biotechnology Co., LTD. FHC cells were cultured in DMEM/F12 (HyClone, USA) containing 10% fetal bovine serum (FBS), cholera toxin (10 ng/ml), insulin (0.005 mg/ml), transferrin (0.005 mg/ml), hydrocortisone (100 ng/ml), and human recombinant EGF (20 ng/ml). HCT116 cells were cultured in McCoy's 5a containing 10% FBS. DLD-1 cells were cultured in 1640 containing 10% FBS. LoVo and SW480 cells were cultured in DMEM containing 10% FBS. Caco-2 cells were cultured in MEM containing 20% FBS. All cell lines were maintained in a humidified chamber containing 5% CO2 at 37 °C.

### Cell transfection

The overexpression plasmid of RFC2 and CREB5 (oe-RFC2 and oe-CREB5), small interfering RNA of RFC2 and CREB5 (si-RFC2 and si-CREB5), and their negative controls were obtained from GenePharma (Shanghai, China). Lipofectamine™ 3000 (Invitrogen) was used for above plasmids transfection.

### Reverse transcription-quantitative polymerase chain reaction (RT-qPCR)

The TRIzol RNA reagent (Invitrogen, Carlsbad, CA) was used to extract RNA from cells. The HiScript II Q Select RT SuperMix (Vazyme, China) was employed to make cDNA. SYBR green (Takara Biotechnology Co., Ltd.) was employed to real-time PCR analysis. RT-qPCR was performed using an ABI StepOnePlus real-time PCR system (Applied Biosystems). The expression of RFC2 was determined by 2^−ΔΔCt^ method. RFC2, forward primer: CTACGAACTGCCGTGGGTT, reverse primer: GAGGGCCCGCAATGATGATG. β-actin, forward primer: AACACCCCAGCCATGTACGTT, reverse primer: CCATCTCTTGCTCGAAGTCCA.

### Cell viability

Cells (2 × 10^4^ cells/well) were seeded in 96-well plates. At 0, 48, and 72 h, 10 µl of CCK-8 reagent (Beyotime, Shanghai, China) was added to each well for 2 h. The absorbance of each well was measured at 450 nm using a microplate reader (Bio-Rad, Hercules, CA, USA).

### Apoptosis assay

Cells were suspended in buffer and stained with FITC Annexin V Apoptosis Detection Kit (Beyotime) in dark place for 10 min. Apoptosis was measured using flow cytometry (BD Biosciences).

### Wound healing and invasion assays

For wound healing assays, until 95% of the cells covered the 6-well plate, wound was produced by scratching the cell monolayer with 10 μl pipette tips. Wound images were taken with a microscope at 0 and 24 h. For invasion assays, Matrigel (BD Biosciences) was added to the upper chamber. Cells were shifted to the upper chamber, while medium containing 10% FBS was added to the lower chamber. After 24 h, cells were stained with crystal violet solution for 2 h. The pictures were taken under a microscope (Olympus, Tokyo, Japan).

### Chromatin immunoprecipitation (ChIP) assay

The assay was performed using ChIP Assay Kit (Beyotime, China). Chromatin was immunoprecipitated using polymerase II, CREB5, or IgG antibody. RFC2 expression was evaluated using RT-qPCR.

### Dual-luciferase assay

Wild type (wt) and mutant type (mut) promoter region of RFC2 were cloned into pGL3-report vector (GenePharma). HEK-293 T cells were co-transfected with RFC2-wt, RFC2-mut, ov-CREB5, or ov-NC plasmids using Lipofectamine™ 3000 (Invitrogen). To examine luciferase activity, dual-luciferase reporter assay system (Promega) was used at 24 h post-transfection.

### Western blot

Cells were lysed in RIPA, and protein concentration was detected by a BCA Kit (Beyotime). Proteins were separated by SDS-PAGE and further transferred onto PVDF membranes (Millipore, MA, USA). Membranes were incubated overnight at 4 °C with RFC2 (Abcam, 1:1000, ab251796), CRBB5 (Abcam, 1:1000, ab168928), LDHA (Abclonal, 1:1000, A1146), GLUT1 (Abcam, 1:1000, ab115730), HK2 (Abclonal, 1:1000, A0994), p-PI3K (Abcam, 1:1000, ab182651), PI3K (Abcam, 1:1000, ab191606), p-AKTSer473 (Cell Signaling Technology (CST), 1:1000, 4060), AKT (Proteintech, 1:1000, 10,176–2-AP), p-mTOR (Abcam, 1:1000, ab109268), mTOR (Proteintech, 1:1000, 28,273–1-AP), p-70S6K (CST, 1:1000, 9205), 70S6K (CST, 1:1000, 9202), MET (Proteintech, 1:1000, 25,869–1-AP), and β-actin antibody (Proteintech, 1:10,000, 20,536–1-AP). Membranes were incubated with secondary antibody (Abcam, 1:10,000, ab6721). The proteins were detected using a GEL imaging system (Bio-Rad, CA, USA).

### Glucose uptake and lactate production

Glucose uptake and lactate production were determined in the supernatants from the cells according to the protocols of Glucose Uptake Assay Kit and Lactate Assay Kit (Abcam, USA).

### Statistical analysis

GraphPad Prism 8.0 software was used to conduct statistical analyses. The differences among groups were analyzed using the student’s t-test or one-way ANOVA. *p* < 0.05 was defined as statistically significant.

## Results

### RFC2 is upregulated in CRC tissues

**Fig. 1:**
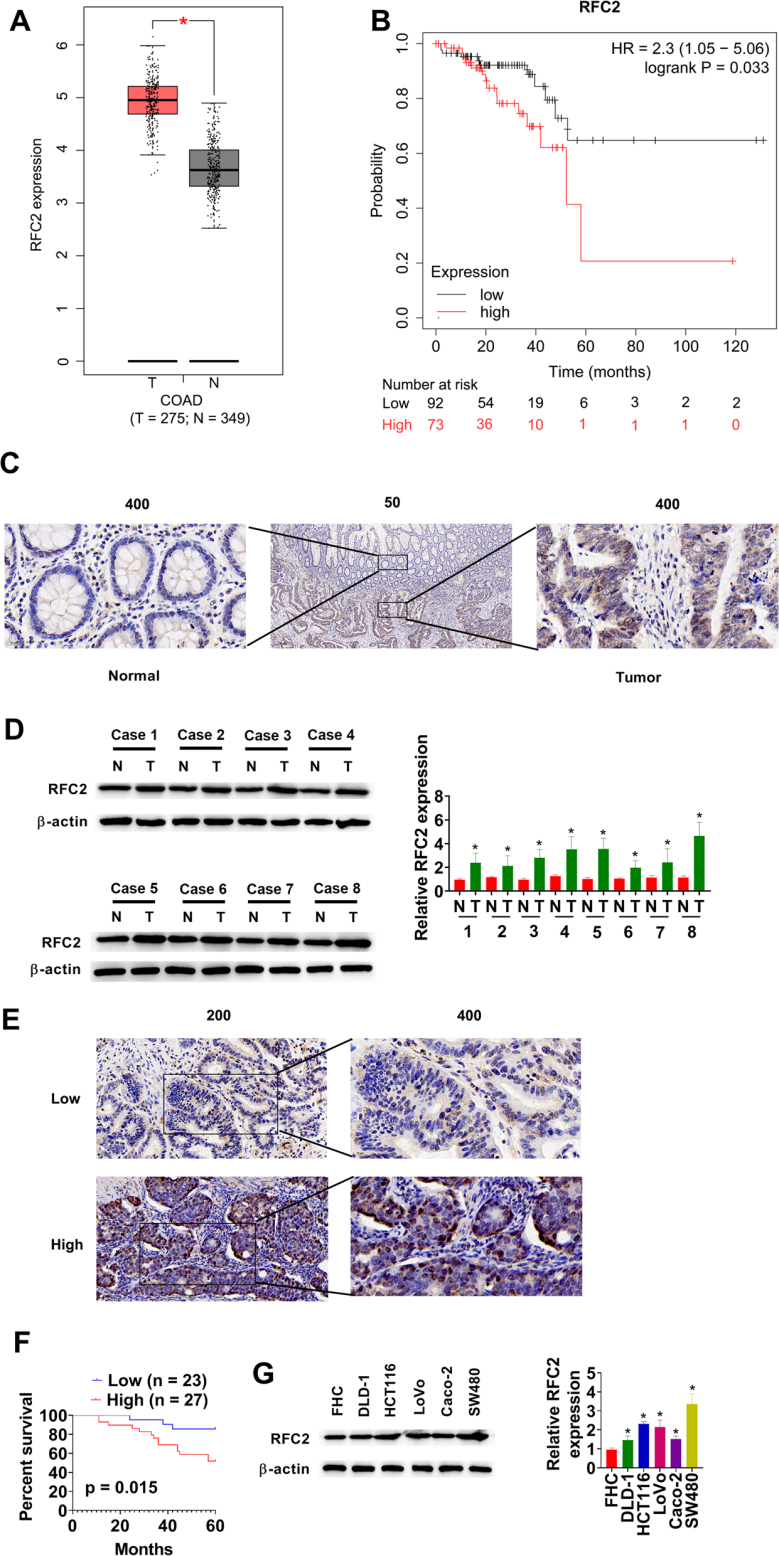
RFC2 is upregulated in CRC tissues. **A** Expression of RFC2 in TCGA-COAD datasets. **B** Overall survival of patients with CRC in TCGA-COAD datasets, who were divided into high or low RFC2 expression. **C** and **D** RFC2 protein expressions in CRC and matched normal tissues were measured using IHC and western blot. **E** RFC2 protein expression in 50 CRC tissues was assessed by IHC. **F** Kaplan–Meier survival curve of overall survival. **G** RFC2 protein levels were detected in normal human colonic epithelial cells (FHC) and five human CRC cell lines (HCT116, LoVo, SW480, Caco-2, and DLD-1). *p < 0.05 vs. Normal or FHC group

RFC2 expression was analyzed using TCGA dataset. RFC2 was upregulated in CRC tissues compared with normal tissues (Fig. 1A). Higher expression of RFC2 (*p* = 0.033) was found to related with a poor prognosis of CRC (Fig. 1B). RFC2 protein in CRC tissues was higher than that in the matched normal tissues (Fig. 1C and D). RFC2 protein expression was also evaluated in 50 CRC patients by IHC (Fig. 1E). Kaplan–Meier analysis revealed that high RFC2 expression in CRC was correlated with poor overall survival (Fig. 1F). Compared with that in normal human colonic epithelial cells (FHC), RFC2 was highly expressed in CRC cell lines (DLD-1, HCT116, LoVo, Caco-2, and SW480) (Fig. 1G). RFC2 expression was particularly high in HCT116 and SW480 cells, compared with other cell lines. We selected HCT116 and SW480 cells to further investigate the roles of RFC2 in CRC.

### RFC2 promotes proliferation, migration, and invasion of CRC cells

**Fig. 2:**
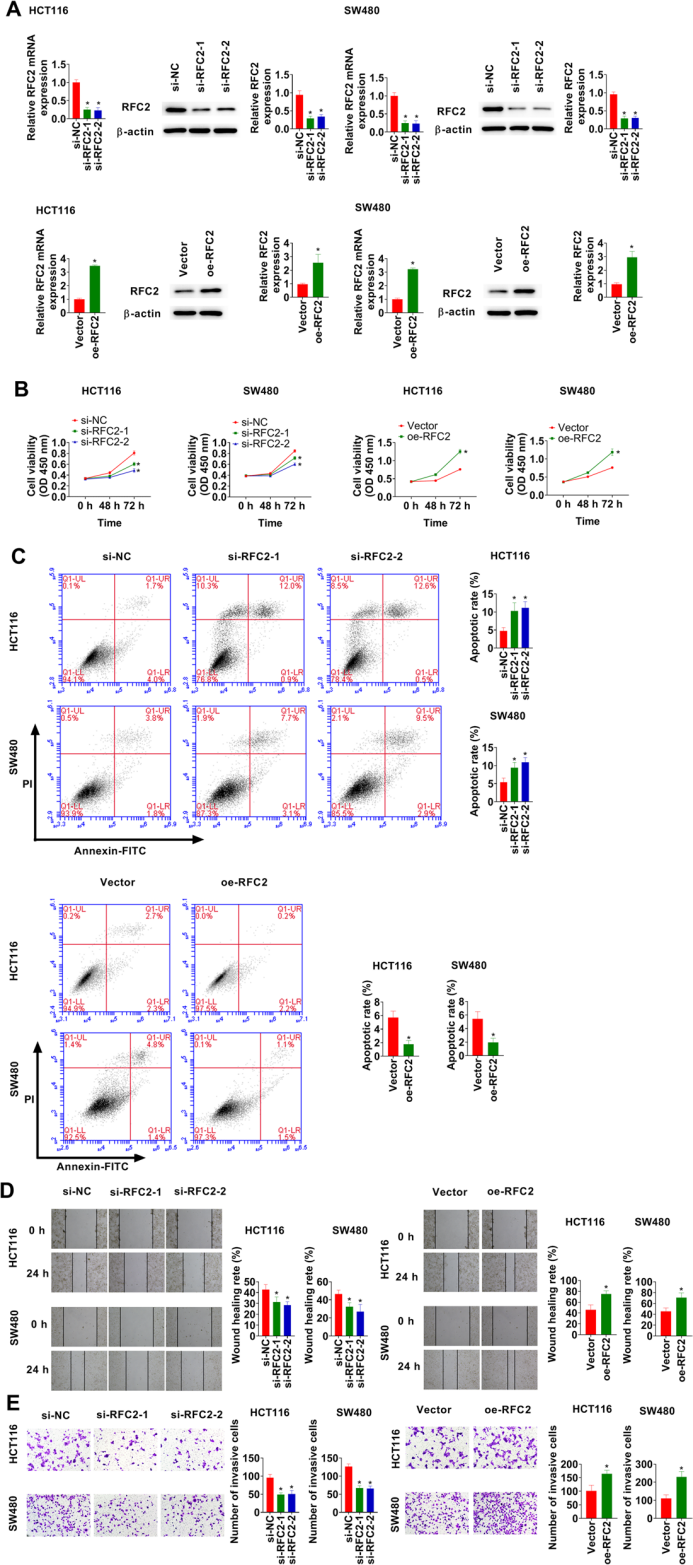
RFC2 promotes proliferation, migration, and invasion of CRC cells. **A** RFC2 mRNA and protein expressions were determined in HCT116 and SW480 cells. **B** Cell viability was examined using CCK-8 assay. **C** Apoptosis was detected using flow cytometry. **D** Wound healing assay was conducted in HCT116 and SW480 cells. **E** Transwell assay was performed in HCT116 and SW480 cells. *p < 0.05 vs. si-NC or Vector group

si-RFC2 and oe-RFC2 were transfected in HCT116 and SW480 cells. The efficacy was validated using RT-qPCR and western blot (Fig. 2A). Silencing of RFC2 dramatically decreased cell viability, whereas, overexpression of RFC2 promoted cell viability (Fig. 2B). RFC2-knockdown considerably induced apoptosis, while, RFC2-overexpression reduced apoptosis (Fig. 2C). RFC2 knockdown dramatically decreased migration and invasion abilities of HCT116 and SW480 cells (Fig. 2D and E). In contrast, RFC2 overexpression promoted migration and invasion in HCT116 and SW480 cells (Fig. 2D and E).

### CREB5 regulates RFC2 transcriptional activity

**Fig. 3:**
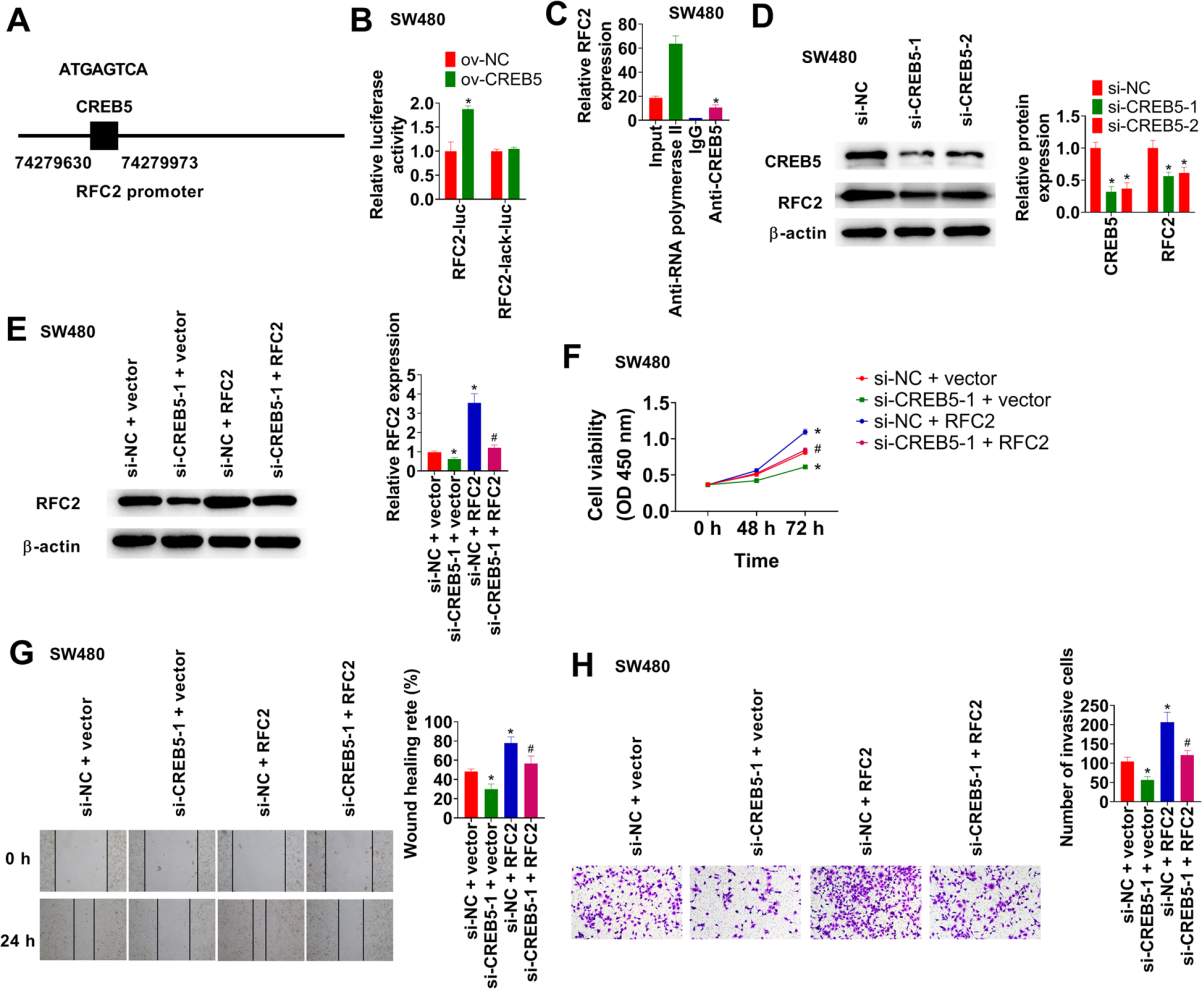
CREB5 regulates RFC2 transcriptional activity. **A** A sketch map of RFC2 promoter region. **B** The ov-CREB5 or ov-NC-transfected cells were transfected with promoter WT or mutant and luciferase activity was detected using dual-luciferase reporter assay. **C** ChIP assay was performed in cells. **D** Western blot of CREB5 and RFC2 in cells. **E** RFC2 protein in cells was measured using western blot. **F** Cell viability was examined using CCK-8 assay. **G** Wound healing assay was conducted in cells. **H** Transwell assay was performed in cells. *p < 0.05 vs. ov-NC + RFC2-luc, lgG, si-NC, or si-NC + vector group. #*p* < 0.05 vs. si-NC + RFC2 group

A sketch map of RFC2 promoter region was shown in Fig. 3A. We performed a luciferase activity assay using HEK-293 T cells to verify the binding of CREB5 in the RFC2 promoter region. CREB5 overexpression increased RFC2 transcriptional activity (Fig. 3B). ChIP assay results demonstrated that CREB5 interacted with RFC2 promoter region (Fig. 3C). Next, we knocked down CREB5 and detected RFC2 expression in SW480 cells. Both CREB5 and RFC2 levels were decreased when CREB5 was silenced (Fig. 3D). CREB5 knockdown reversed increased RFC2 expression induced by oe-RFC2 (Fig. 3E). CREB5 knockdown inhibited cell proliferation, migration, and invasion, which were blocked by RFC2 overexpression (Fig. 3F-H).

### RFC2 promotes aerobic glycolysis in CRC cells

**Fig. 4:**
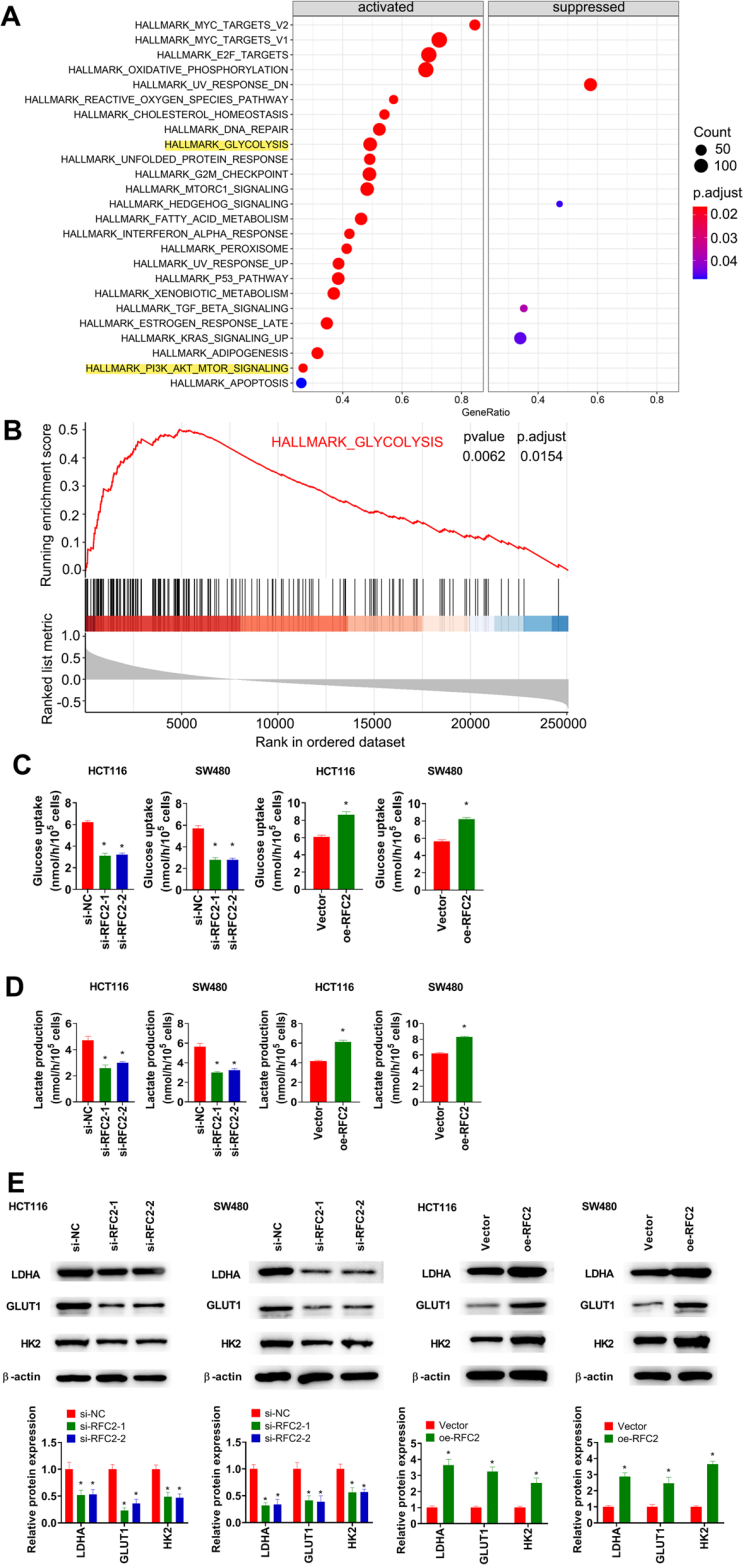
RFC2 promotes aerobic glycolysis in CRC cells. **A** and **B** Results from GSEA forecast. **C** and **D** Glucose uptake and lactate production were detected in cells. **E** Marker proteins of glycolysis were detected using western blot. *p < 0.05 vs. si-NC or Vector group

By using GSEA analyzers based on RFC2 expression in CRC from the TCGA database, RFC2-enriched pathways are shown in Fig. 4A. GSEA indicated that RFC2 was associated heavily with glycolysis (Fig. 4B). Compared with si-NC treatment, si-RFC2 treatment in HCT116 and SW480 cells was associated with a remarkable decrease in glucose uptake and lactate production; however, compared with vector treatment, oe-RFC2 treatment in HCT116 and SW480 cells resulted in a notable induction in glucose uptake and lactate production (Fig. 4C and D). RFC2 knockdown remarkably decreased LDHA, GLUT1, and HK2 expression in HCT116 and SW480 cells; whereas their expression was significantly increased after upregulation of RFC2 (Fig. 4E).

### RFC2 activates the MET/PI3K/AKT/mTOR pathway in CRC cells

**Fig. 5:**
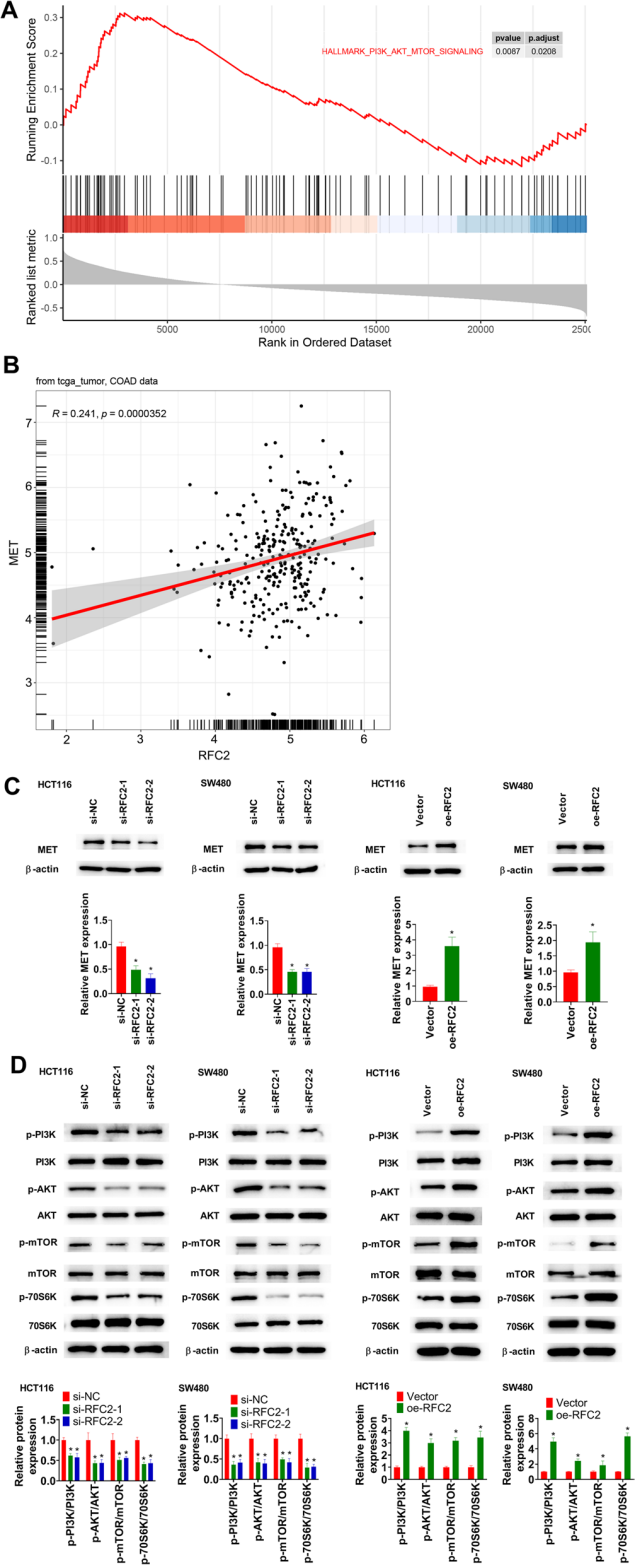
RFC2 activates the MET/PI3K/AKT/mTOR pathway in CRC cells. **A** Results from GSEA forecast. **B** Correlation analysis between RFC2 and MET based on TCGA-COAD datasets using Pearson’s correlation analysis. **C** MET protein expression in cells was measured using western blot. **D** Representative protein expression in the PI3K/AKT/mTOR pathway was measured using western blot. *p < 0.05 vs. si-NC or Vector group

Based on the GESA forecast, RFC2 activated the PI3K/AKT/mTOR pathway in CRC (Fig. 5A). RFC2 expression was positively correlated with MET expression (Fig. 5B). RFC2 knockdown restrained MET protein expression in both HCT116 and SW480 cell lines, and its overexpression significantly enhanced MET protein expression (Fig. 5C). RFC2 knockdown decreased the levels of p-PI3K, p-AKT, p-mTOR, and p-70S6K in both HCT116 and SW480 cell lines, whereas RFC2 overexpression induced above levels (Fig. 5D).

### Activator or inhibitor partially rescues the effect of RFC2 knockdown or overexpression in CRC cells

**Fig. 6:**
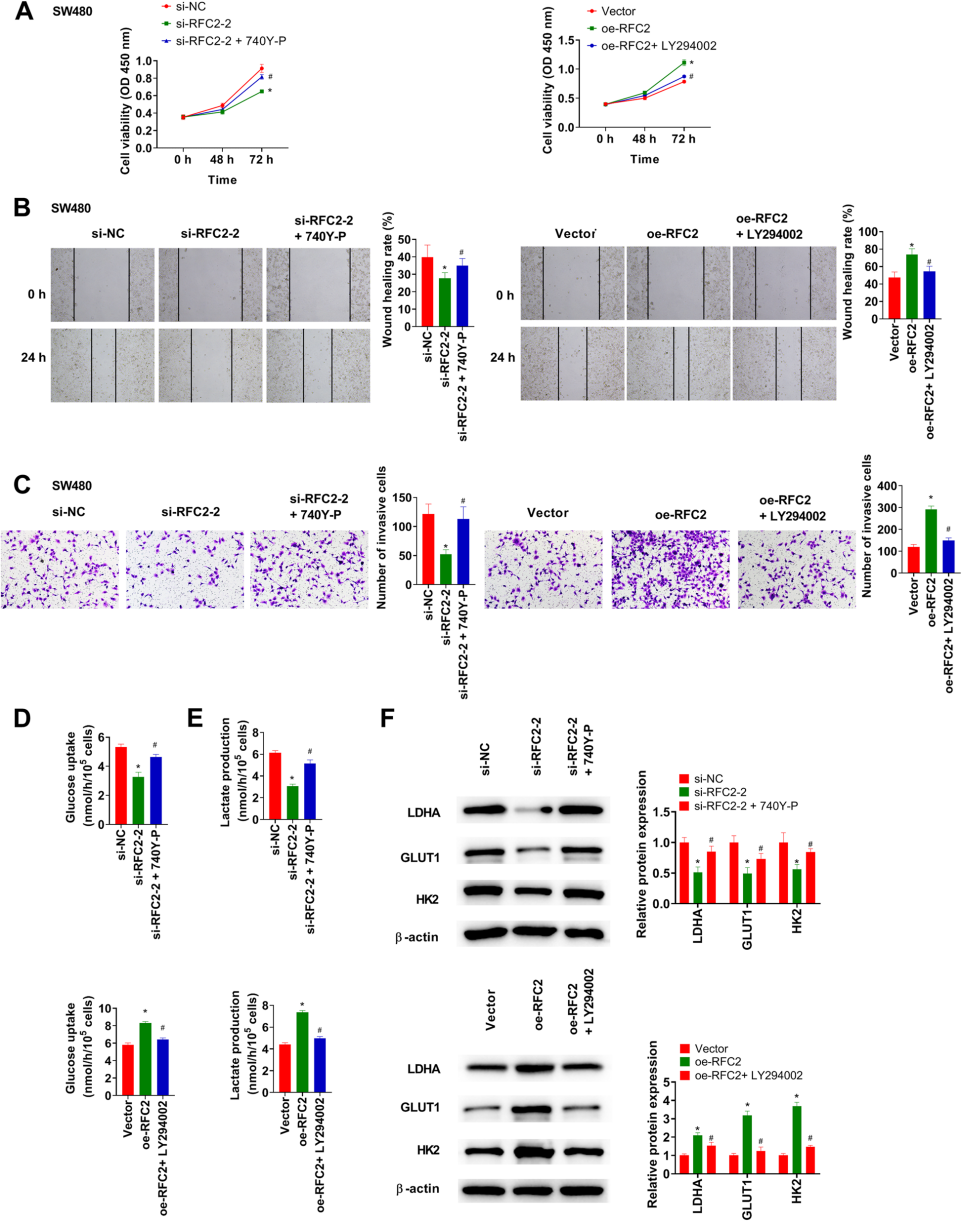
Activator or inhibitor partially rescues the effects of RFC2 knockdown or overexpression in CRC cells. **A** Cell viability was examined using CCK-8 assay. **B** and **C** Wound healing and transwell assays were conducted in SW480 cells. **D** and **E** Glucose uptake and lactate production were detected in SW480 cells. **F** Marker proteins of glycolysis were detected using western blot. *p < 0.05 vs. si-NC or Vector group. #*p* < 0.05 vs. si-RFC2-2 or oe-RFC2 group

740Y-P attenuated the decreased cell viability, migration, and invasion caused by knockdown of RFC2. Furthermore, LY294002 led to a decrease in cell viability, migration, and invasion in RFC2-overexpression cells compared with untreated cells (Fig. 6A-C). 740Y-P restored RFC2 silencing-induced inhibition of glucose uptake, lactate production, and LDHA, GLUT1, and HK2 expression. LY294002 reversed RFC2-mediated promotion of glucose uptake, lactate production, and LDHA, GLUT1, and HK2 expression (Fig. 6D-F).

## Discussion

RFC2 is famous for DNA replication and damage repair, and participates in disorder of biological process, such as Williams-Beuren syndrome, type 2 diabetes mellitus, and cancers [25–27]. Previous experimental studies have shown that RFC2 is highly expressed in various cancer, such as sarcoma, diffuse lower-grade gliomas, hepatocellular carcinoma, and CRC [18, 20, 28, 29]. We also found that RFC2 is overexpressed in CRC tissues compared to normal tissues. High expression of RFC2 predicted poor prognosis of patients with CRC. Similarly, high expression of RFC2 is related to worse overall survival of hepatocellular carcinoma [29]. Our results suggested that RFC2 is an oncogene and a promising prognostic biomarker of CRC. In CRC cells, RFC2 aggravated malignant proliferation, migration, and invasion.

TF is important regulators of intrinsic life processes within cells, possess DNA-binding domains and initiate transcription of genes [30]. Over the years, a large amount of data from different laboratories has shown that TFs are involved in progression of cancer [31–33]. For instance, FoxO1 promotes glioma cell proliferation by acting as a transcriptional activator of RFC2 [34]. CREB5 is a TF that mediates gene expression in cells [35]. CREB5 promotes metastasis of CRC by interacting with the MET promoter and activating the MET pathway [22]. CREB5 inhibits mitochondrial apoptosis by promoting the transcription of TOP1MT in head and neck squamous cell carcinoma [23]. Our study found that CREB5 enhanced RFC2 transcriptional activity by interacting with RFC2 promoter in CRC cells. CREB5 knockdown inhibited proliferation, migration, and invasion abilities, which were blocked by RFC2 overexpression.

Aerobic glycolysis is a notable feature of malignant cancers, that enhances unrestricted tumor growth and metastasis [36]. Normally, tumor cells preferentially rely on aerobic glycolysis for the source of energy and nutrition, even when oxygen is abundant [37]. Therefore, targeting glycolysis is a valuable and promising therapeutic strategy for the treatment of CRC. In our study, silencing of RFC2 dramatically decreased glucose uptake and lactate production, whereas, overexpression of RFC2 promoted glucose uptake and lactate production. LDHA, GLUT1, and HK2 expression are elevated in CRC [38–40]. In our study, RFC2 knockdown remarkably decreased LDHA, GLUT1, and HK2 expression in HCT116 and SW480 cells; whereas their expression was significantly increased after downregulation of RFC2. Conformably, Jing et al. have demonstrated that NCAPD3 induces the expression of glycolytic regulators (GLUT1, HK2, ENO1, PKM2 and LDHA), and finally promotes the progression of CRC [41]. We elucidated that RFC2 enhanced aerobic glycolysis in CRC cells.

MET regulates the proliferation, migration, and invasion of cancer cells by mediating the pathways such as PI3K/AKT pathway, MAPK pathway, and FAK pathway [42, 43]. MET is highly expressed in advanced stages of CRC and indicates worse prognosis and mortality [44]. Zhang et al. have revealed that HSF4 facilitates tumor progression of CRC by transactivating MET [45]. In current study, RFC2 expression was positively correlated with MET expression. RFC2 knockdown restrained MET protein expression in both HCT116 and SW480 cell lines, and its overexpression significantly enhanced MET protein expression.

In order to better understand the mechanisms of RFC2 in CRC, pathways were enriched using GSEA. RFC2 expression is connected with the PI3K/AKT/mTOR pathway. Down-regulated phosphorylation of PI3K, AKT, mTOR, and 70S6K in CRC cells leads to inhibition of proliferation and aerobic glycolysis [46, 47]. Li et al. have demonstrated that knockdown FOXO6 suppresses the proliferation, invasion, and glycolysis of CRC cells by inactivating PI3K/Akt/mTOR pathway [48]. In this study, knockdown RFC2 inactivated PI3K/AKT/mTOR pathway. Treatment with 740Y-P (PI3K activator) abrogated phenomena induced by silencing of RFC2, demonstrating that RFC2 could regulate malignant progression of tumor cells through PI3K/AKT/mTOR pathway.

## Conclusion

RFC2 expression was upregulated in CRC. RFC2 promoted proliferation, migration, invasion by affecting aerobic glycolysis and the MET/PI3K/AKT/mTOR pathway in CRC cells. Our research demonstrated that RFC2 might be a candidate gene for the therapy of CRC.

### Supplementary Information

**Additional file 1. **

## Data Availability

The datasets used and/or analysed during the current study are available from the corresponding author on reasonable request.
