# Supplementary material for: RFC2 promotes aerobic glycolysis and progression of colorectal cancer
Source: BMC Gastroenterol. 2023 Oct 11;23:353. doi: 10.1186/s12876-023-02984-0 (PMC10566032; doi:10.1186/s12876-023-02984-0)

Fig1D

RFC2 case 1-4

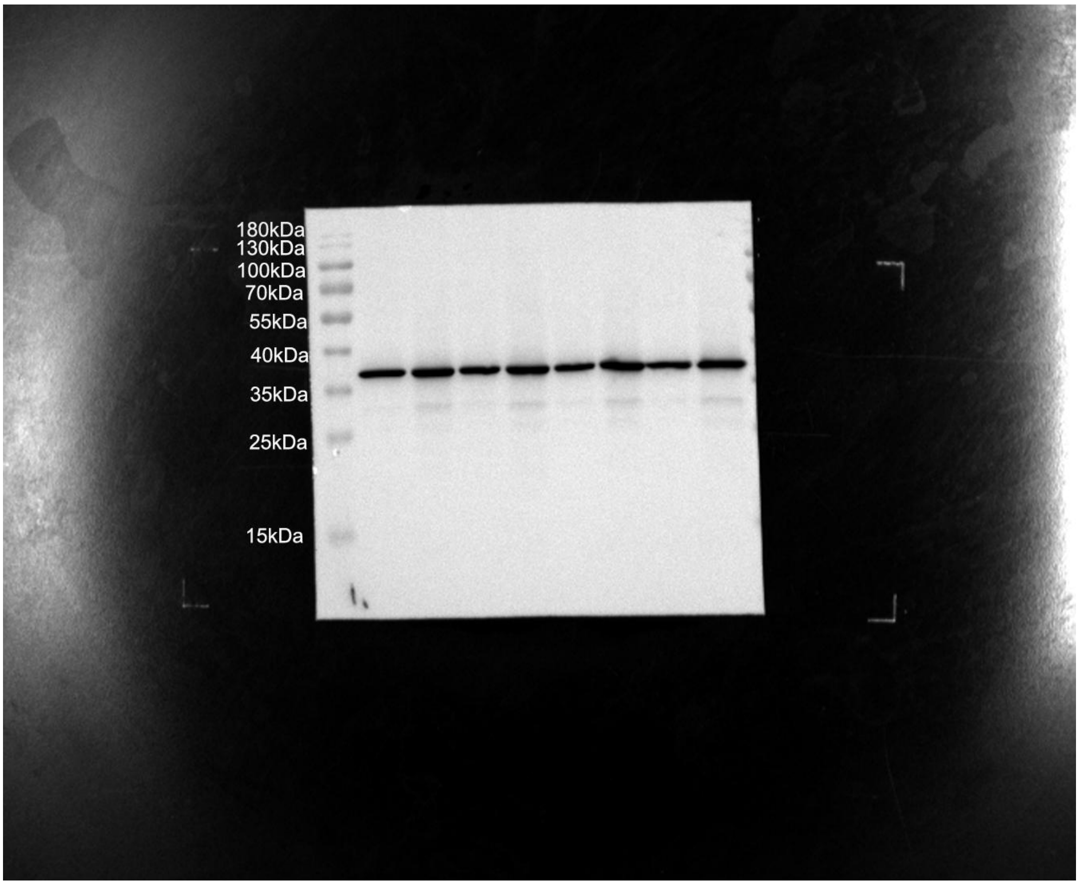

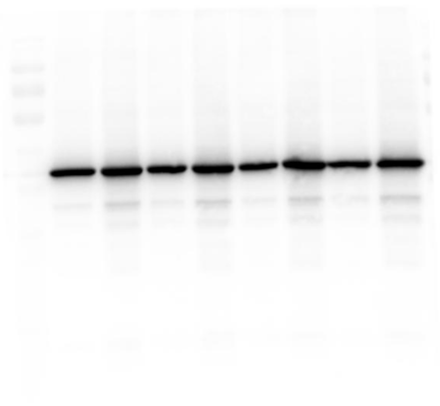

β-actin 1-4

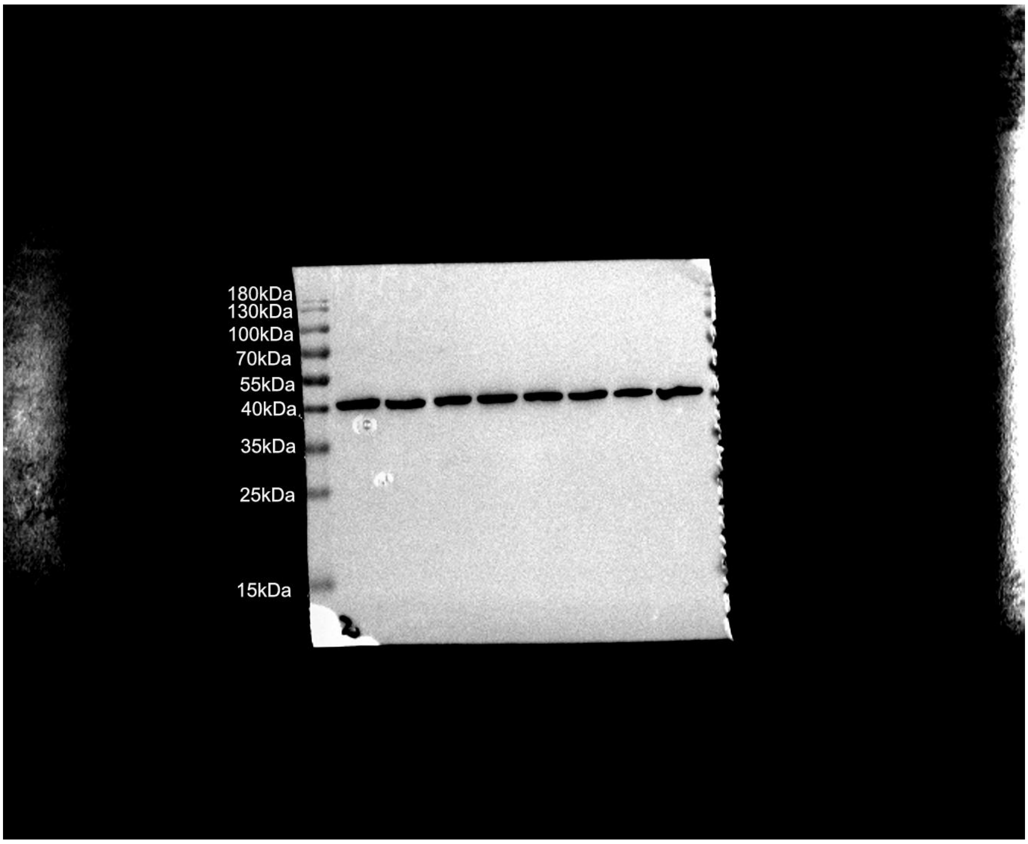

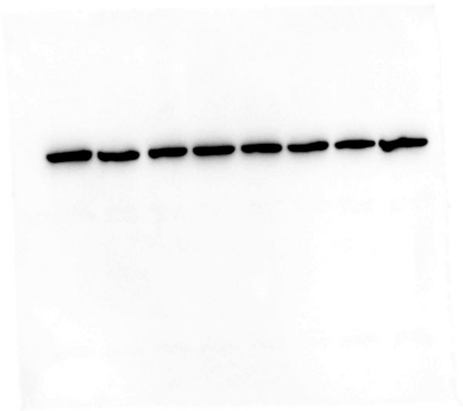

RFC2 5-8

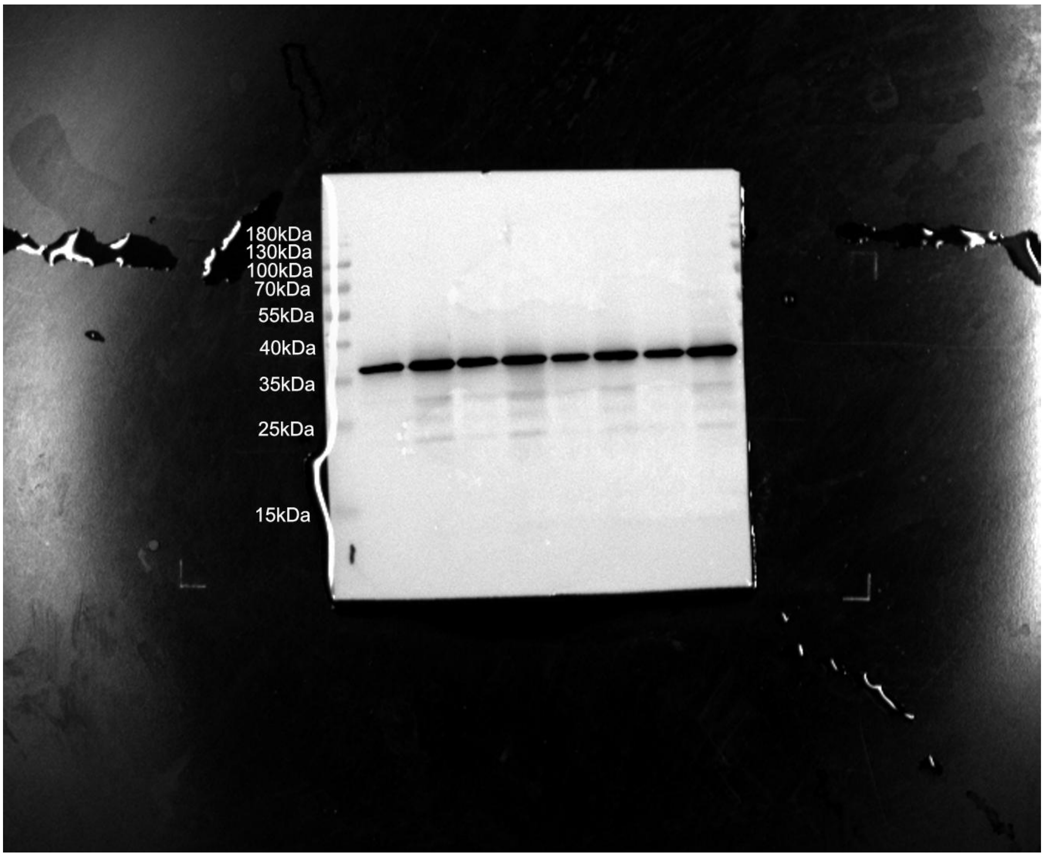

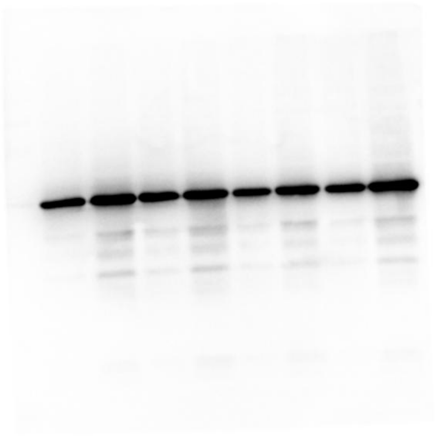

β-actin 5-8

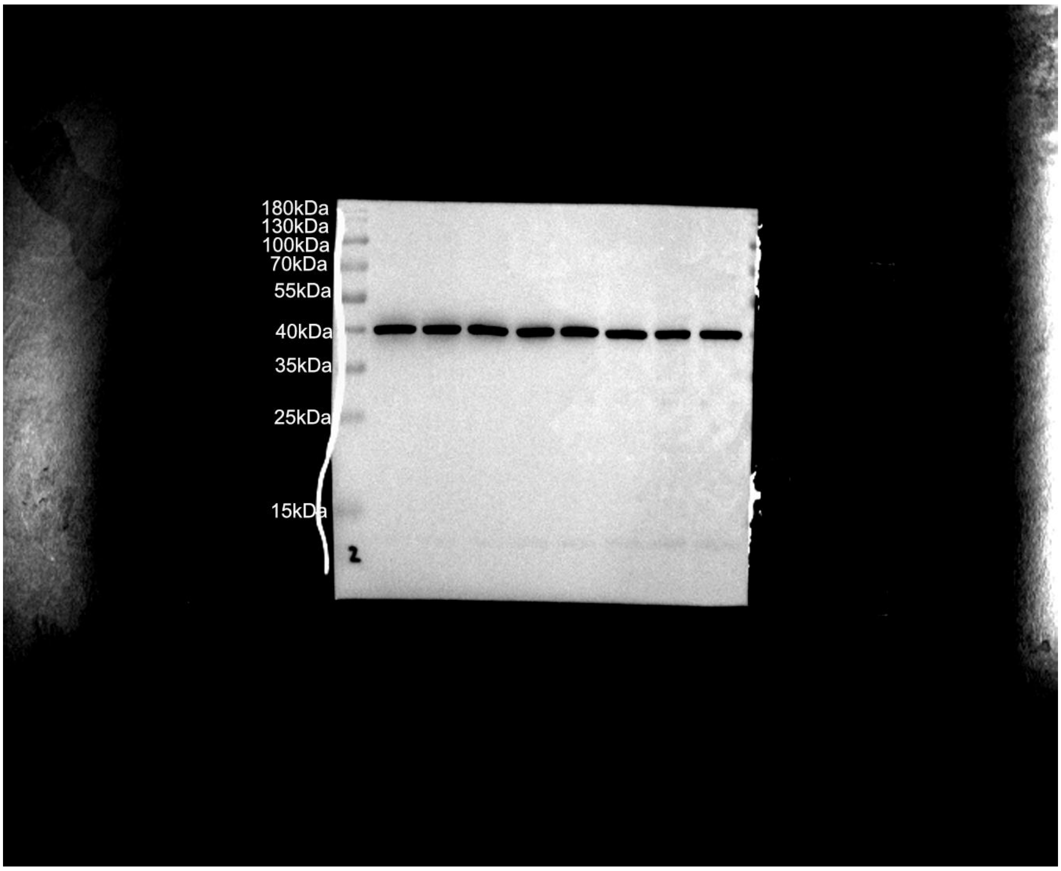

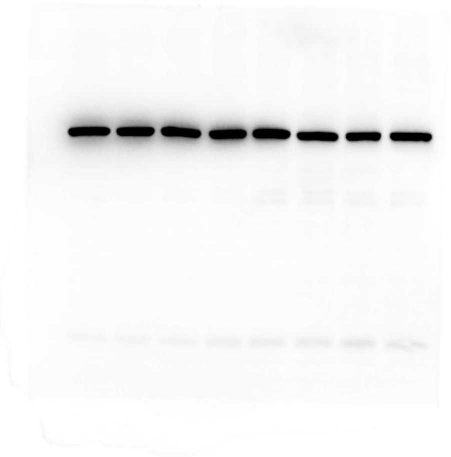

Fig1E RFC2

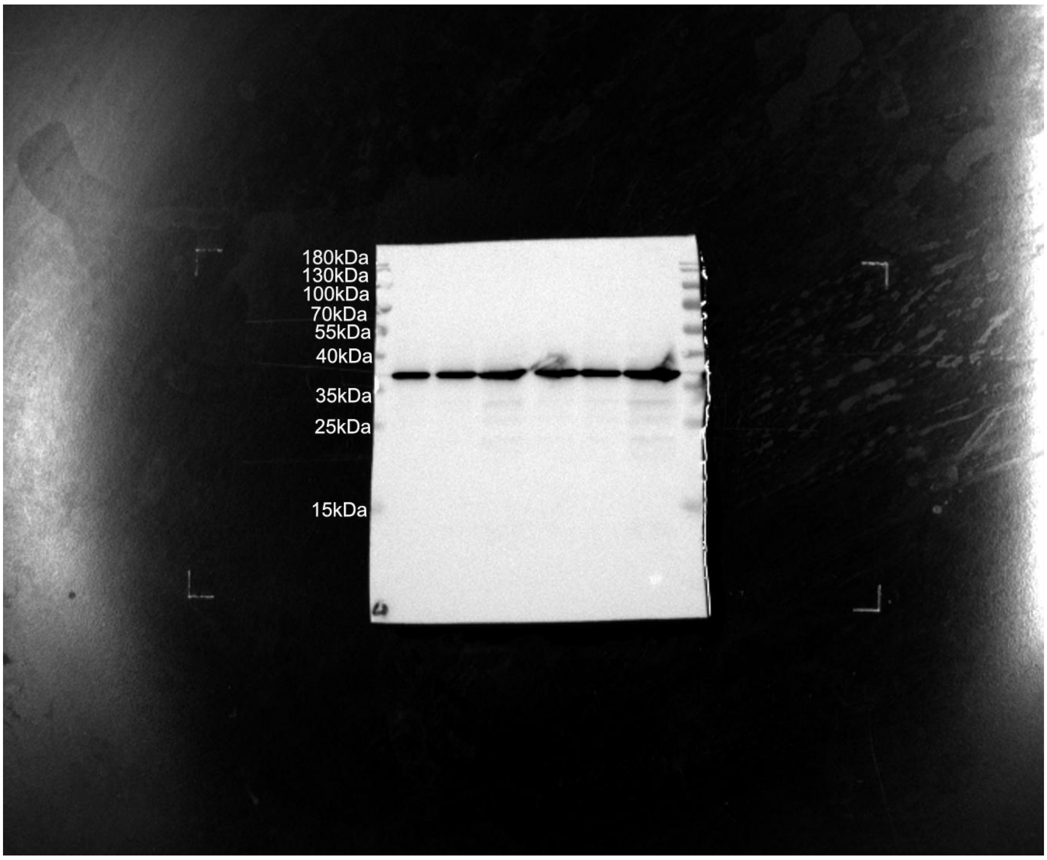

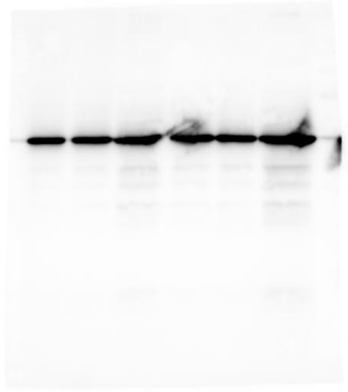

Fig1E β-actin

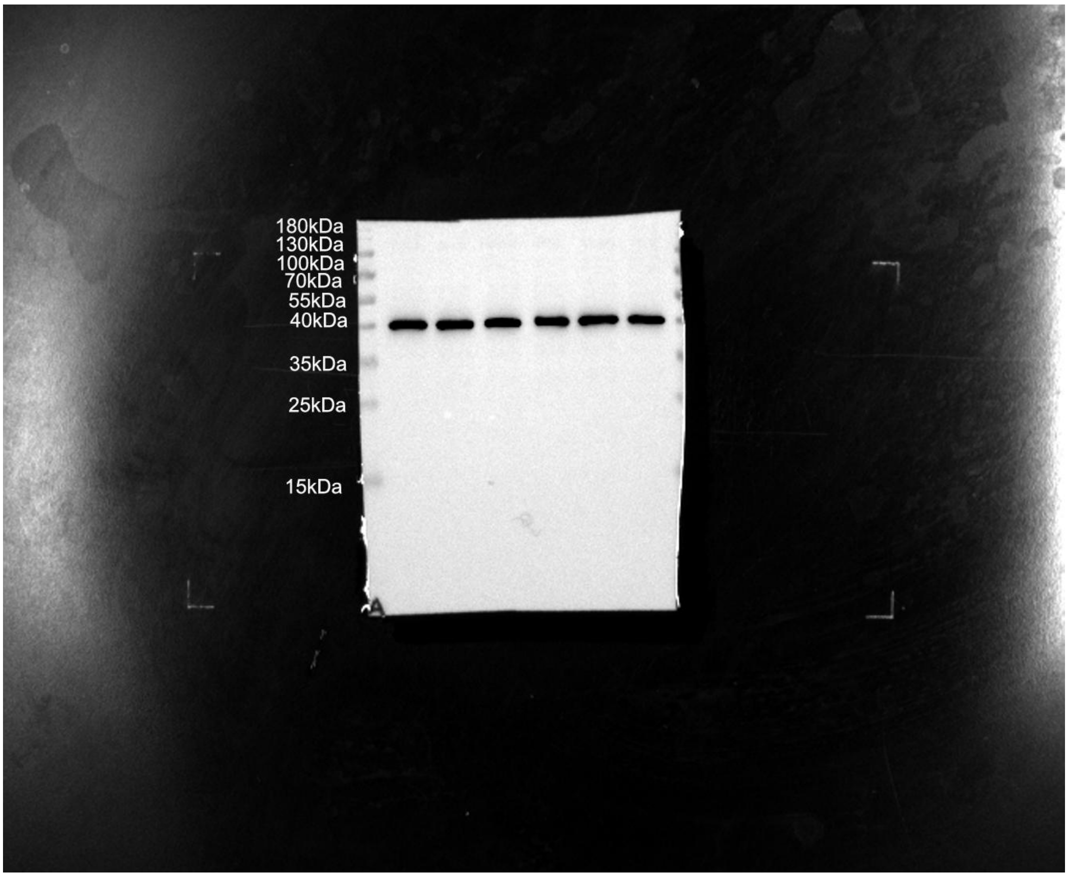

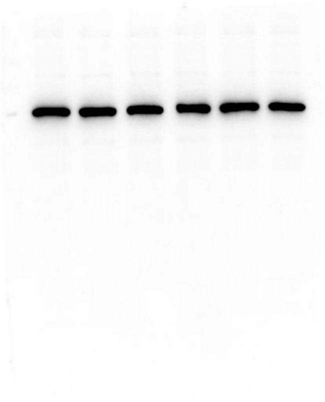

Fig2A HCT116 si-RFC2

RFC2

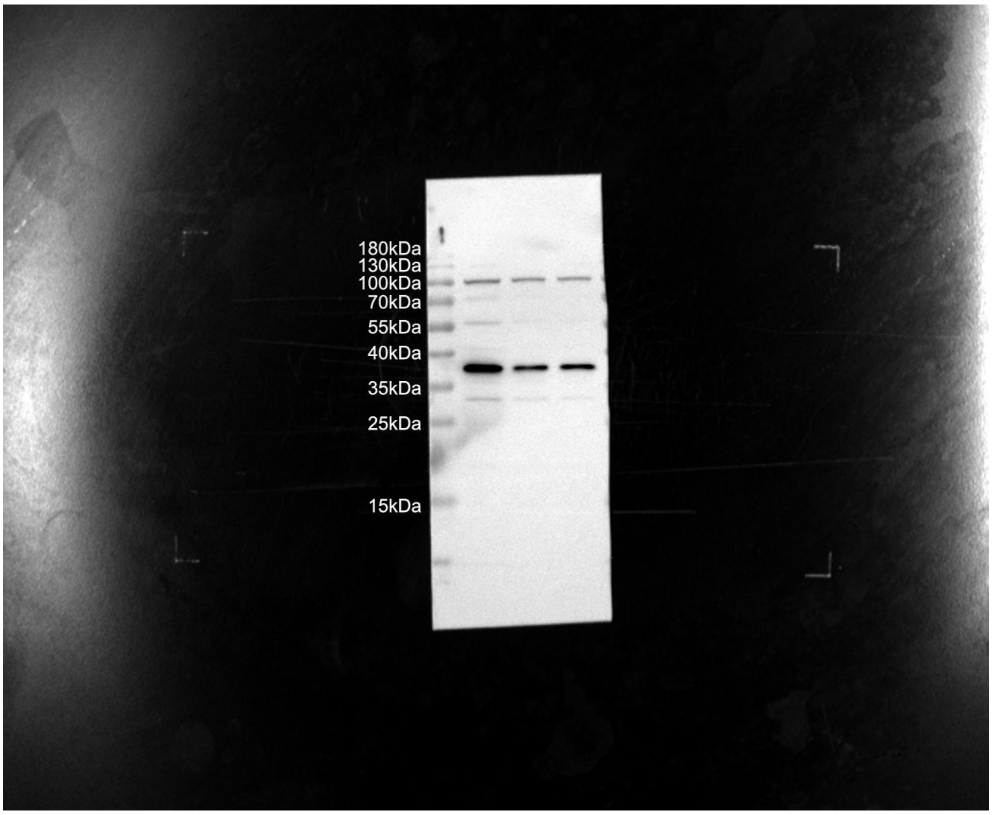

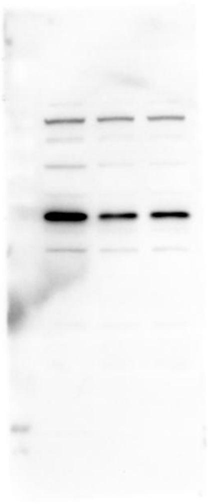

β-actin

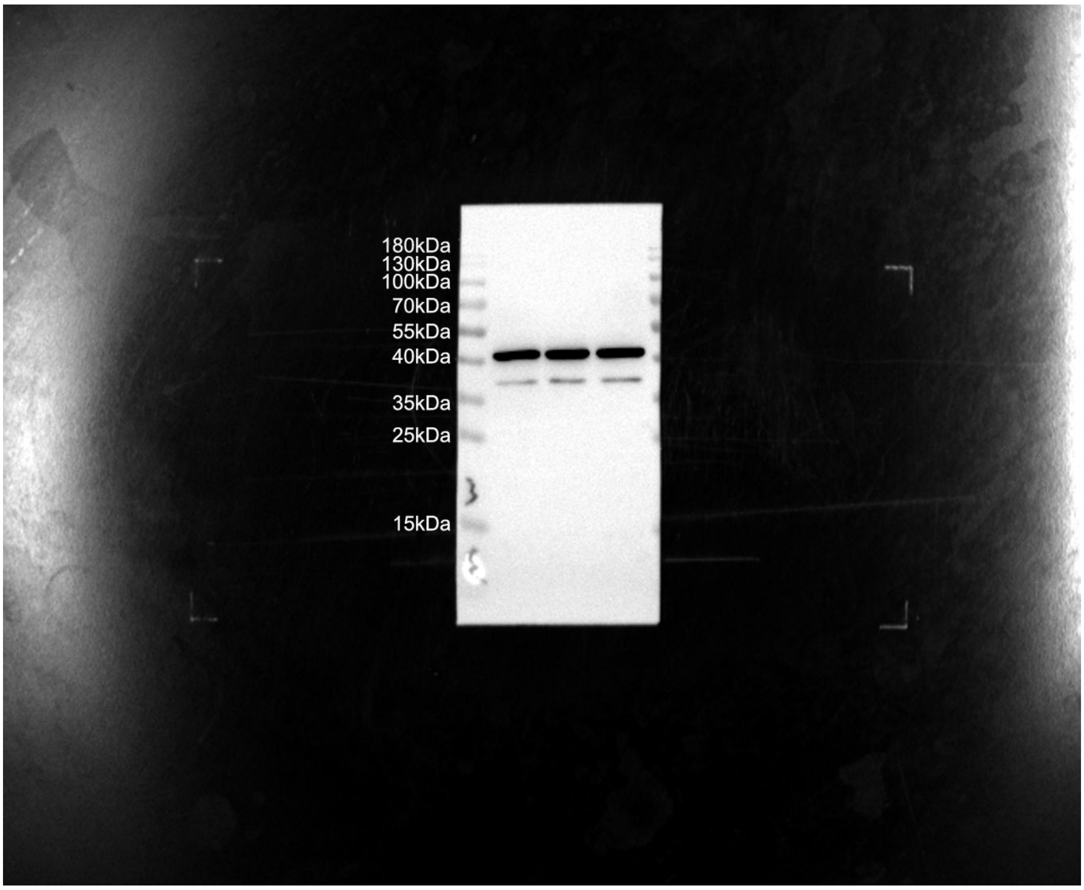

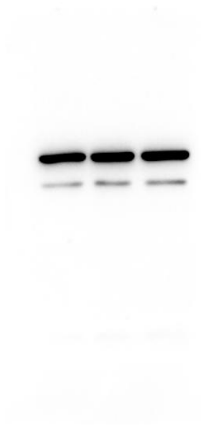

Fig2A SW480 si-RFC2

RFC2

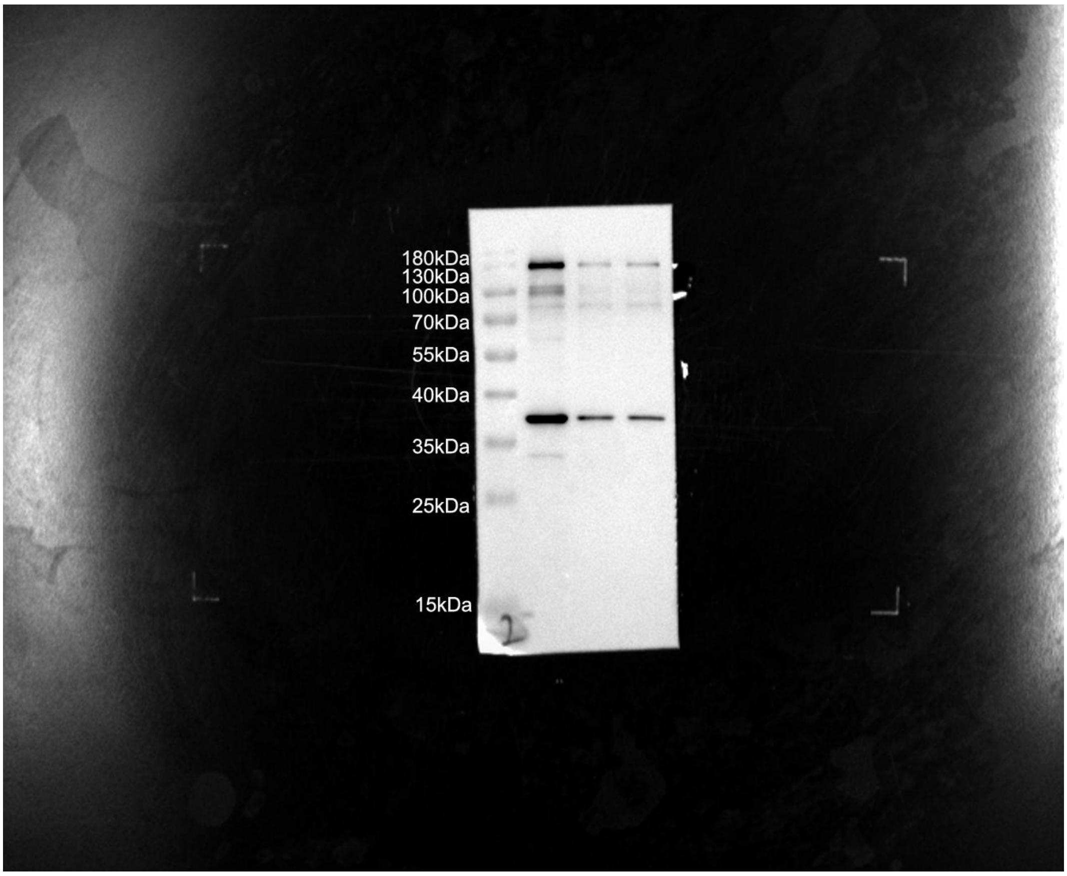

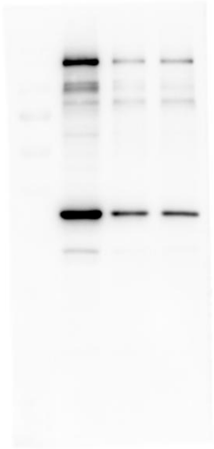

β-actin

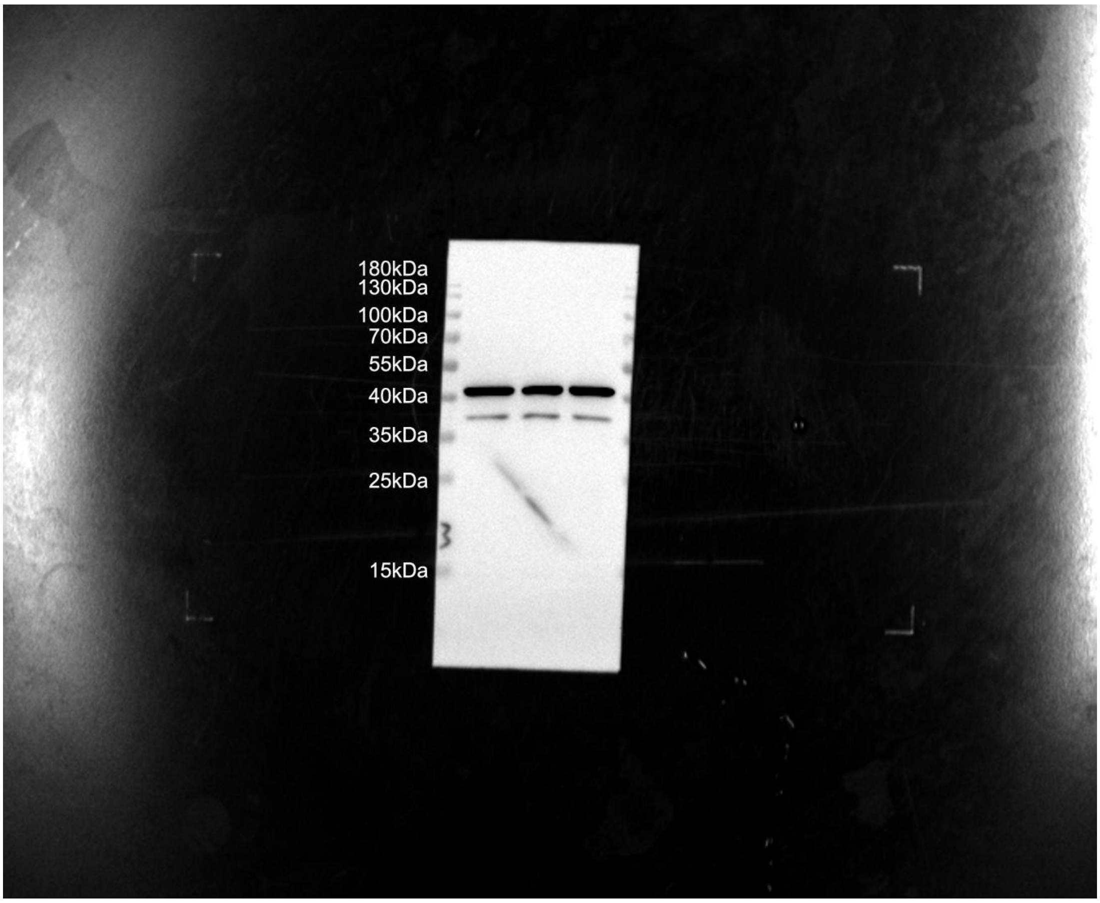

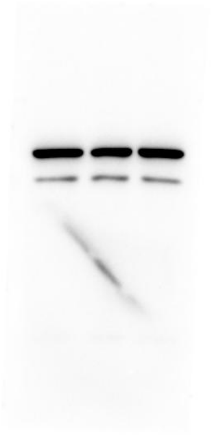

Fig2A HCT116 oe-RFC2

RFC2

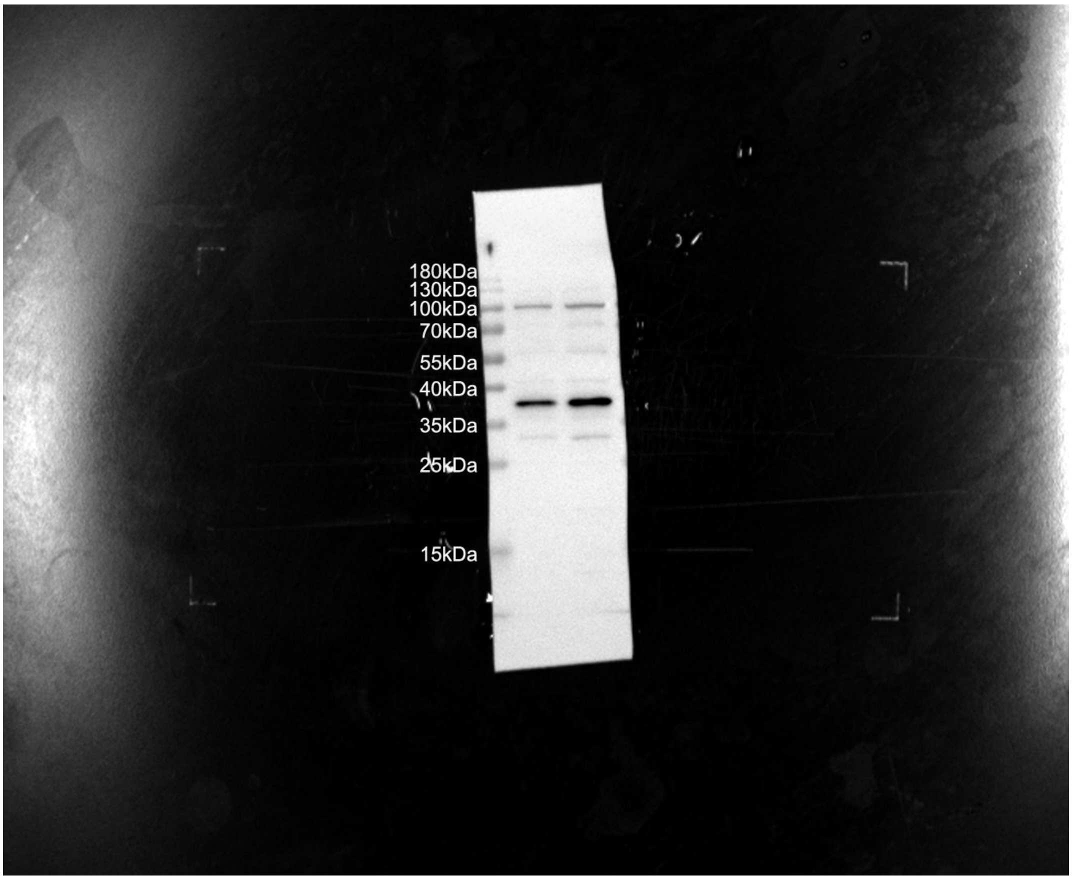

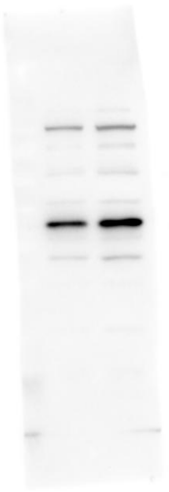

β-actin

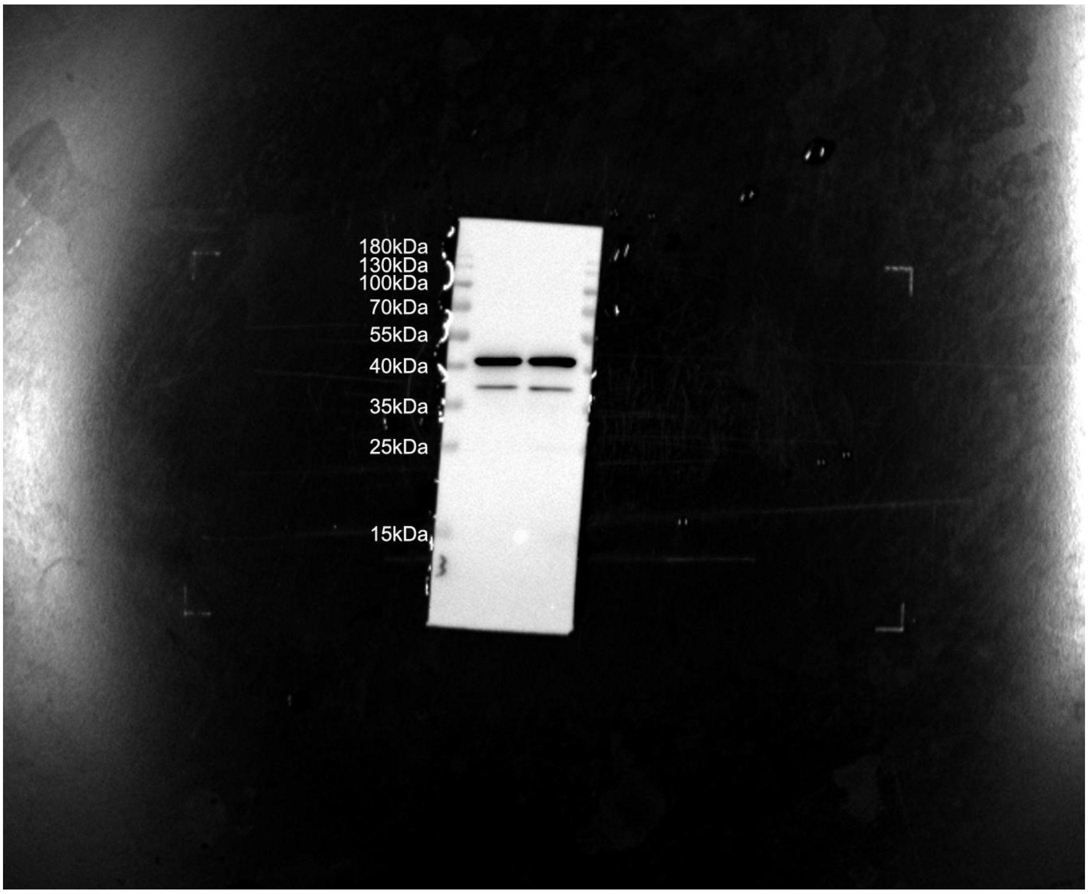

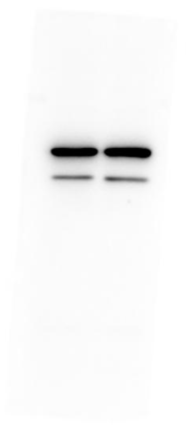

Fig2A SW480 oe-RFC2

RFC2

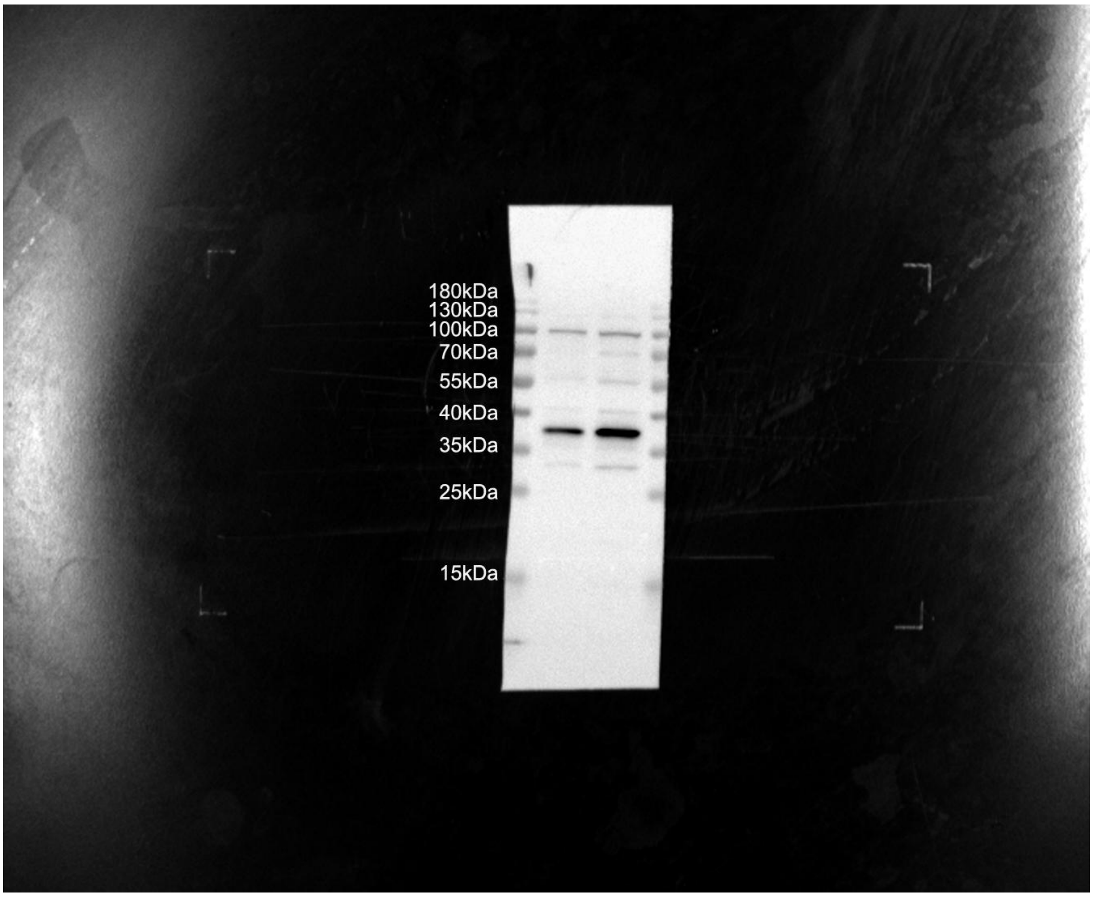

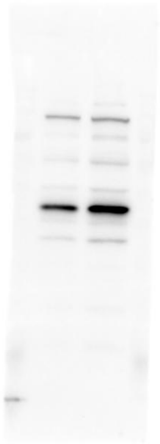

β-actin

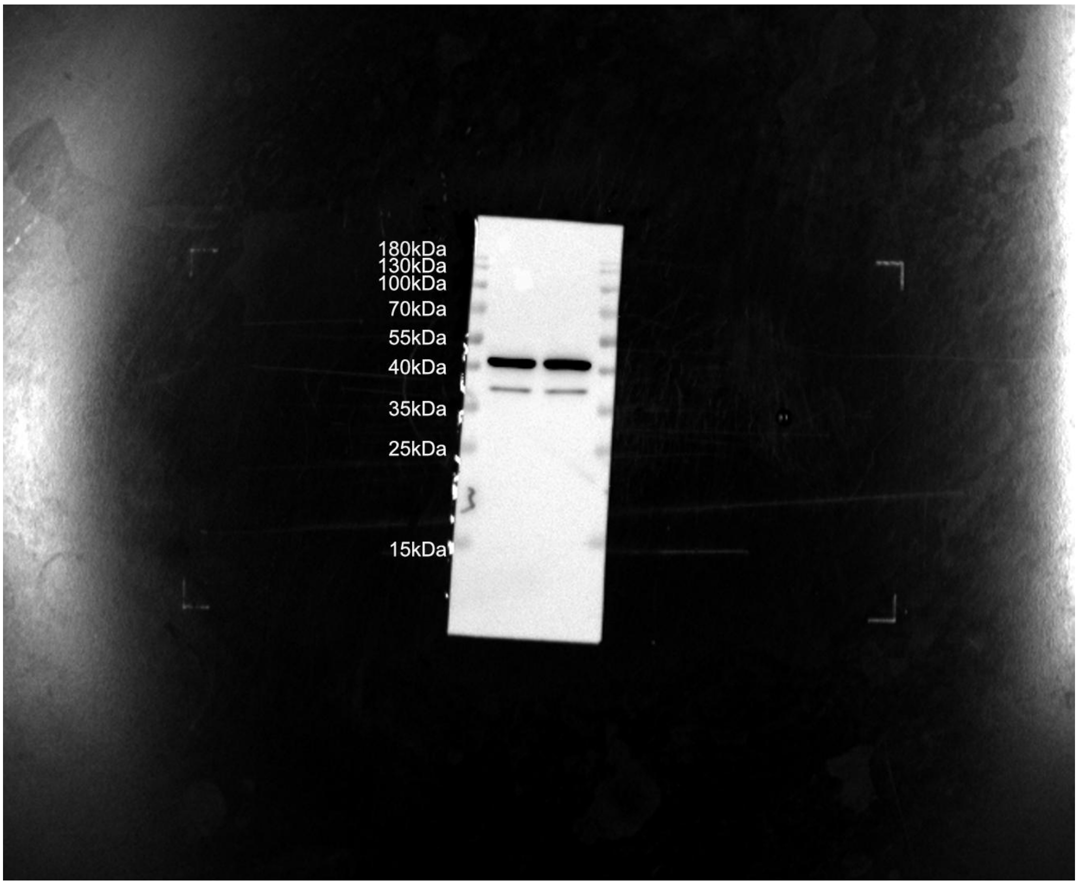

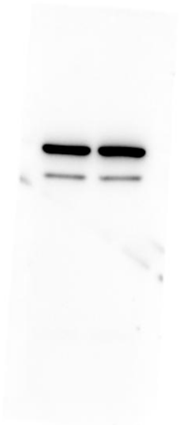

Fig3D SW480

CREB5

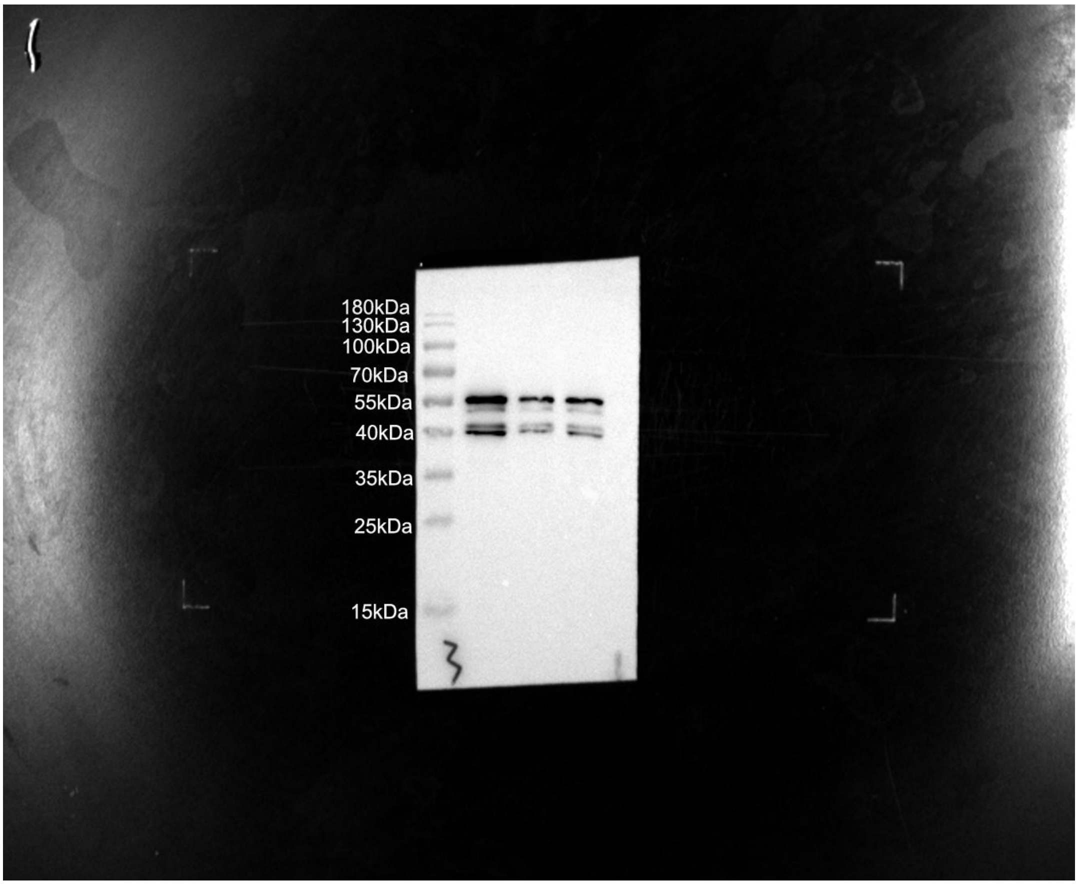

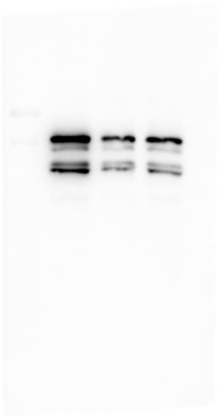

RFC2

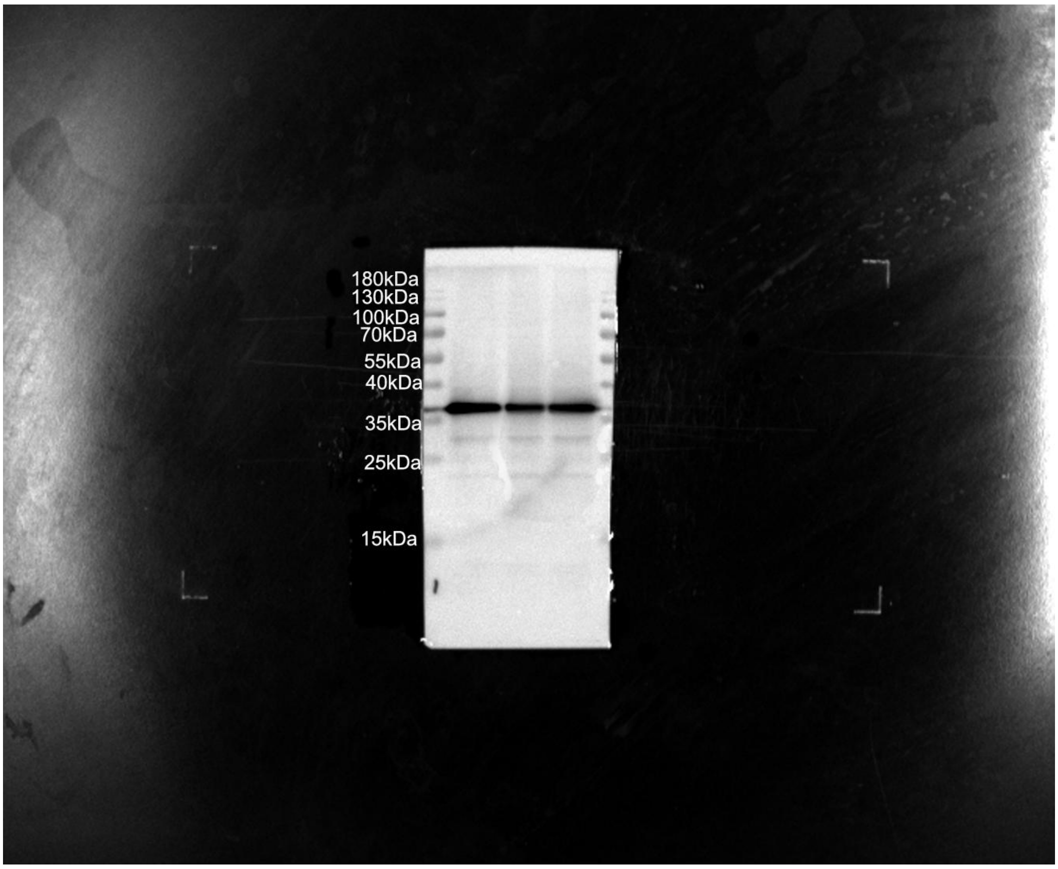

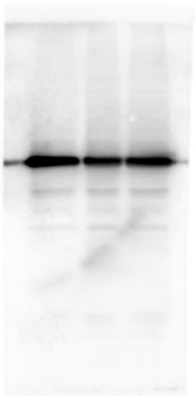

β-actin

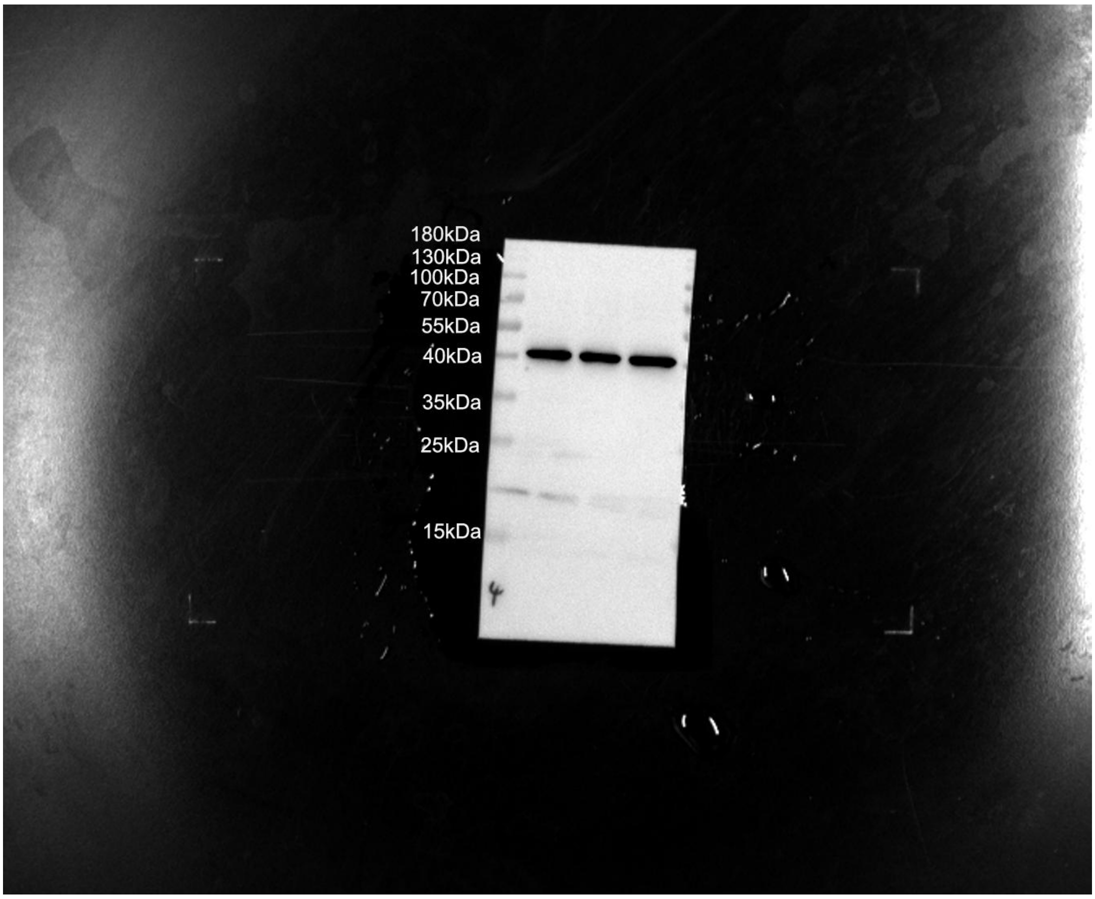

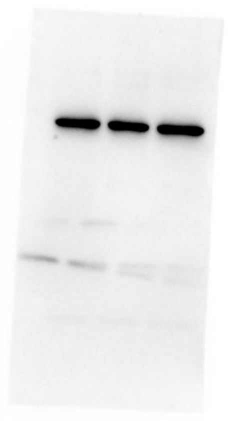

Fig3E

RFC2

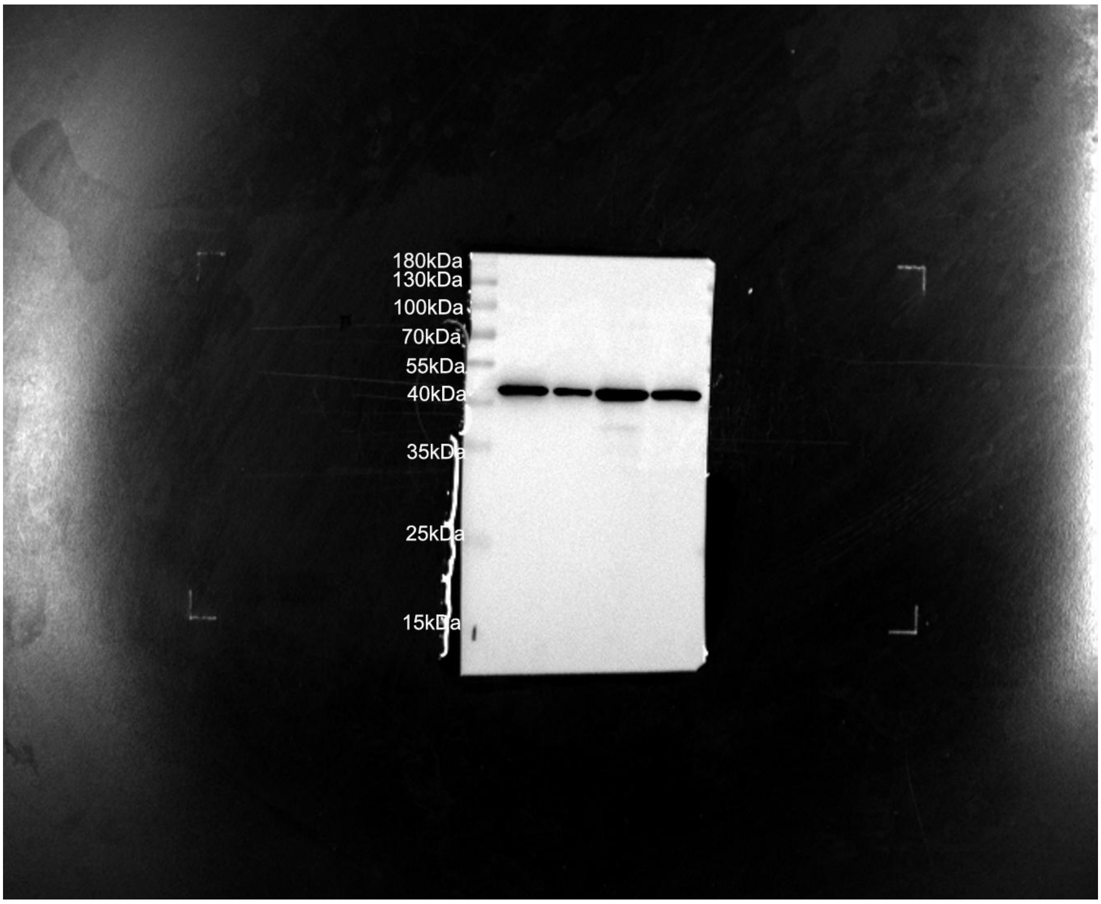

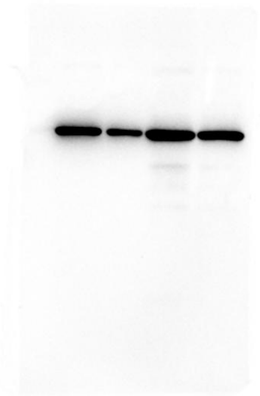

β-actin

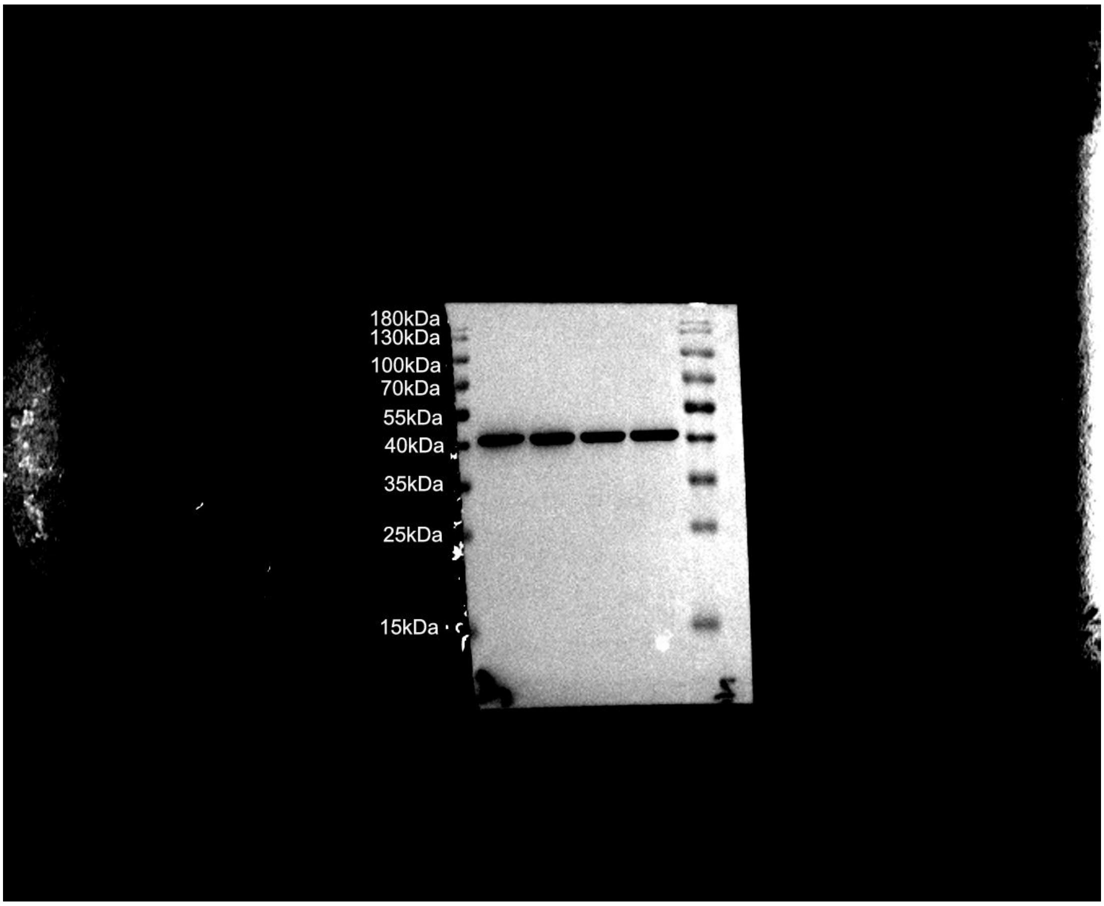

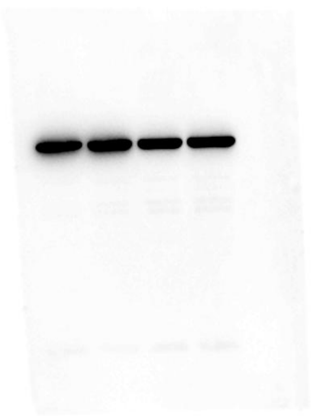

Fig4E HCT116 si-RFC2

LDHA

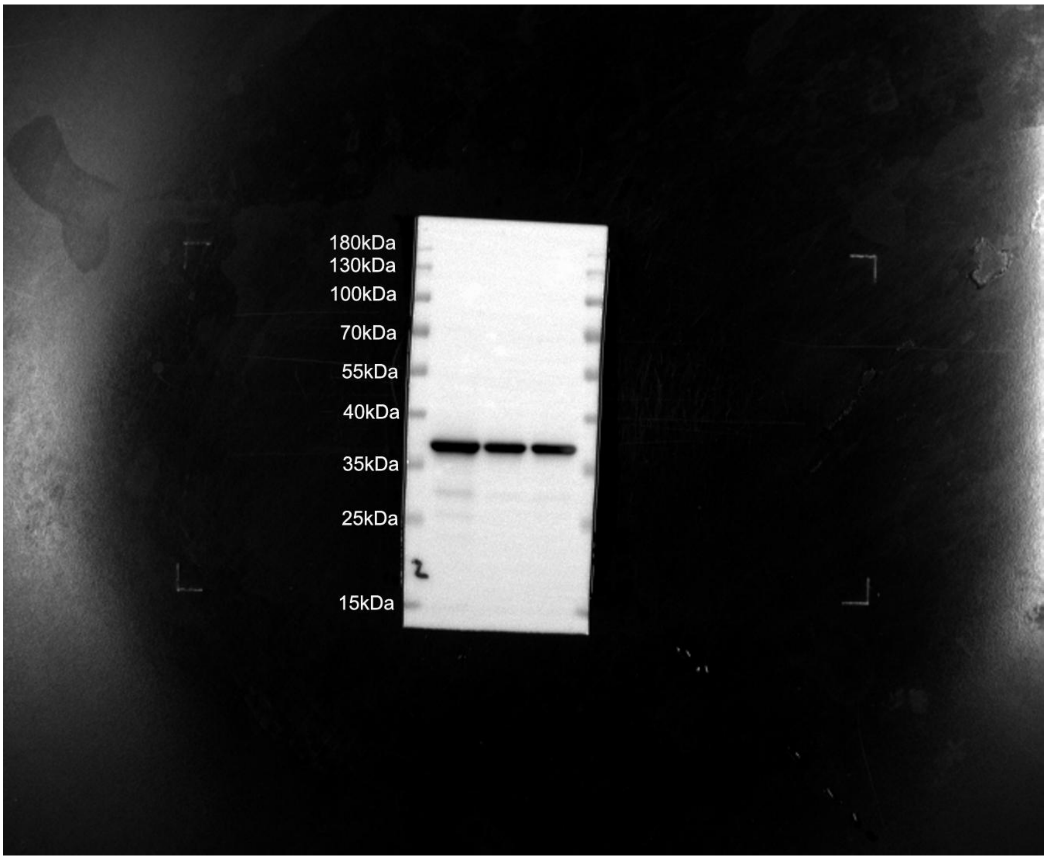

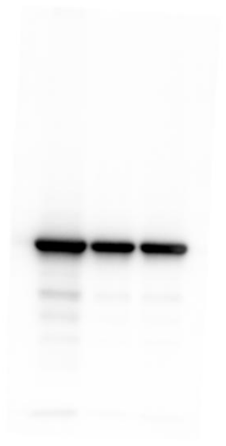

GLUT1

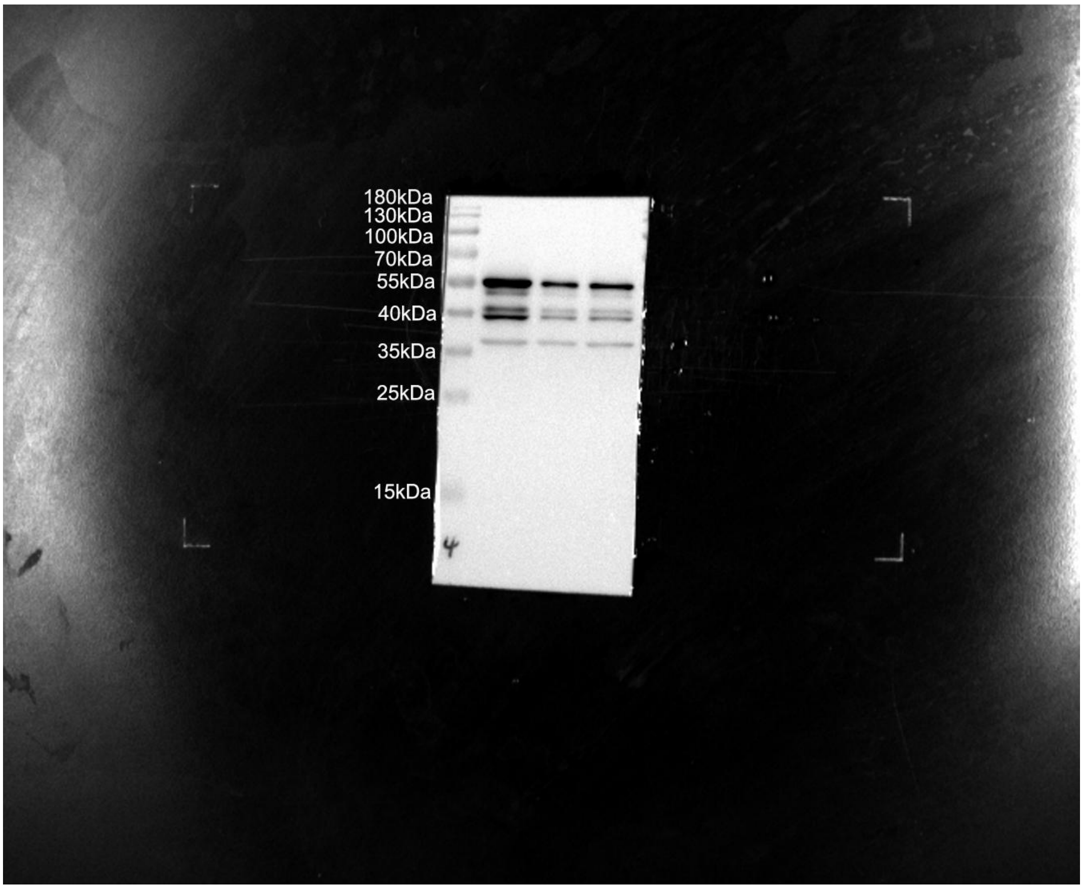

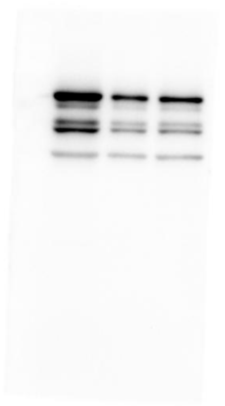

HK2

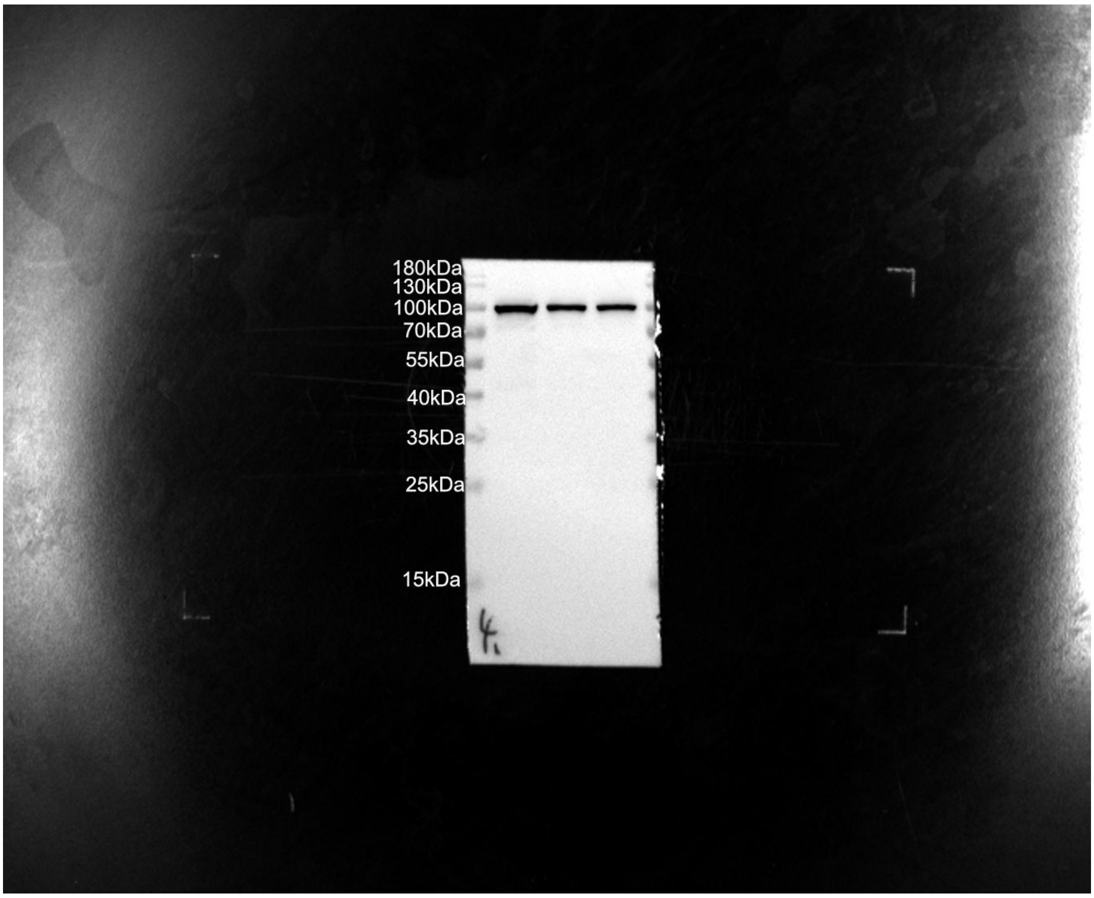

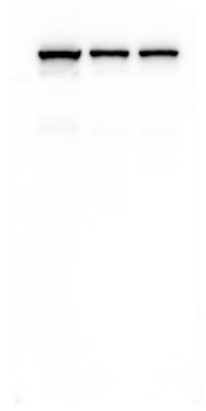

β-actin

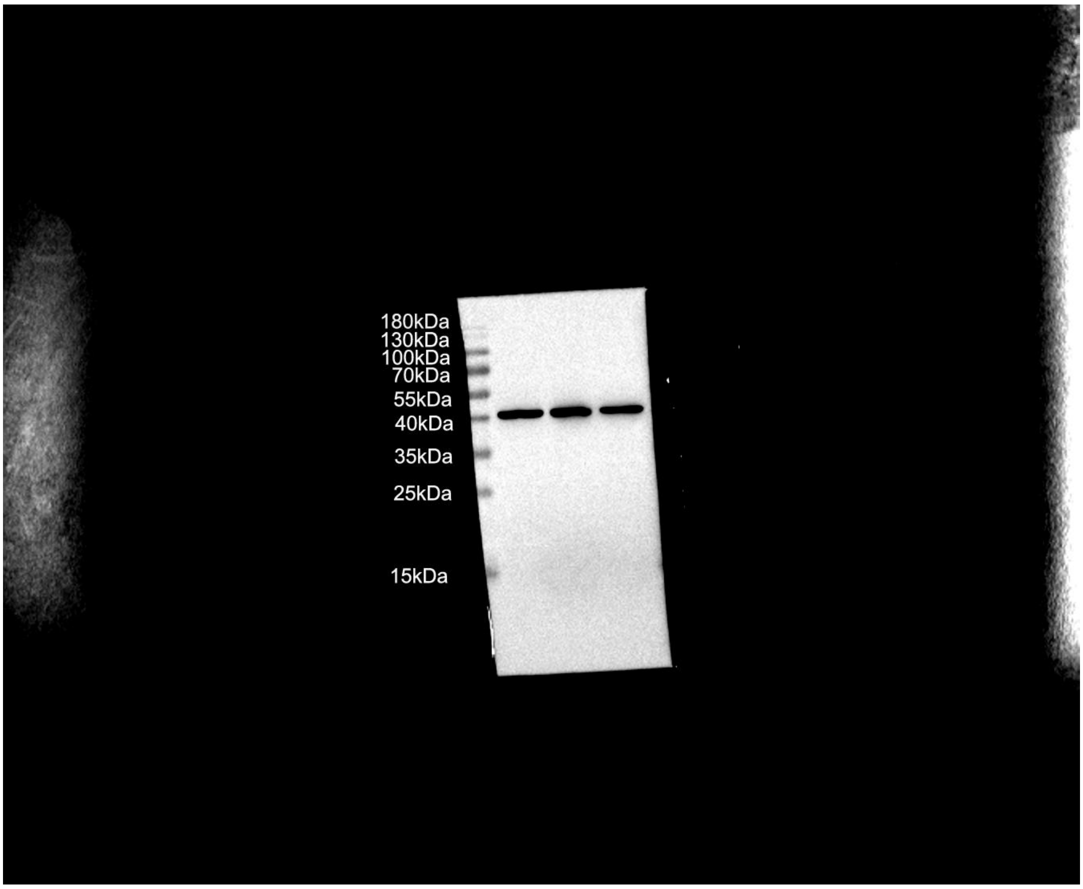

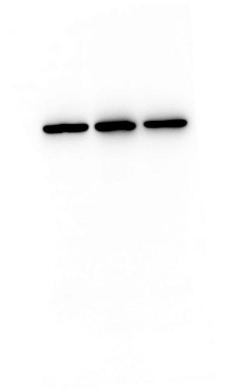

Fig4E SW480 si-RFC2

LDHA

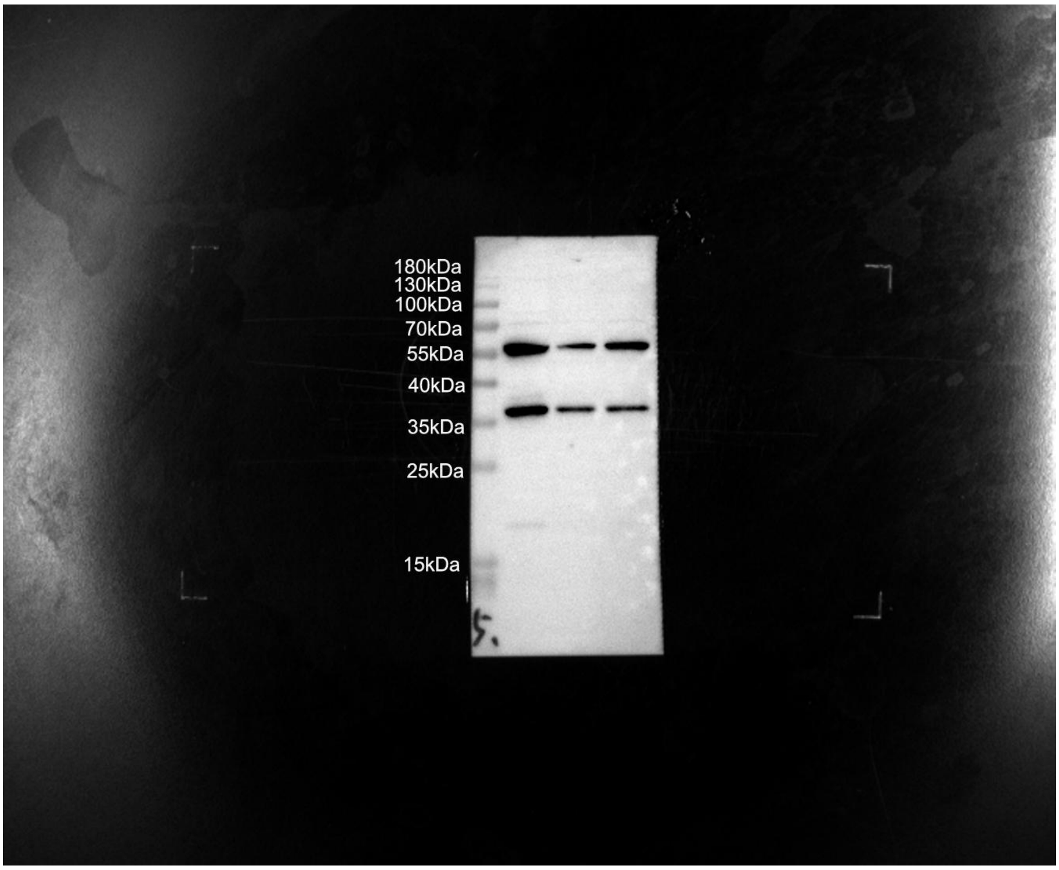

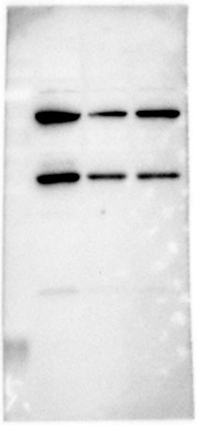

GLUT1

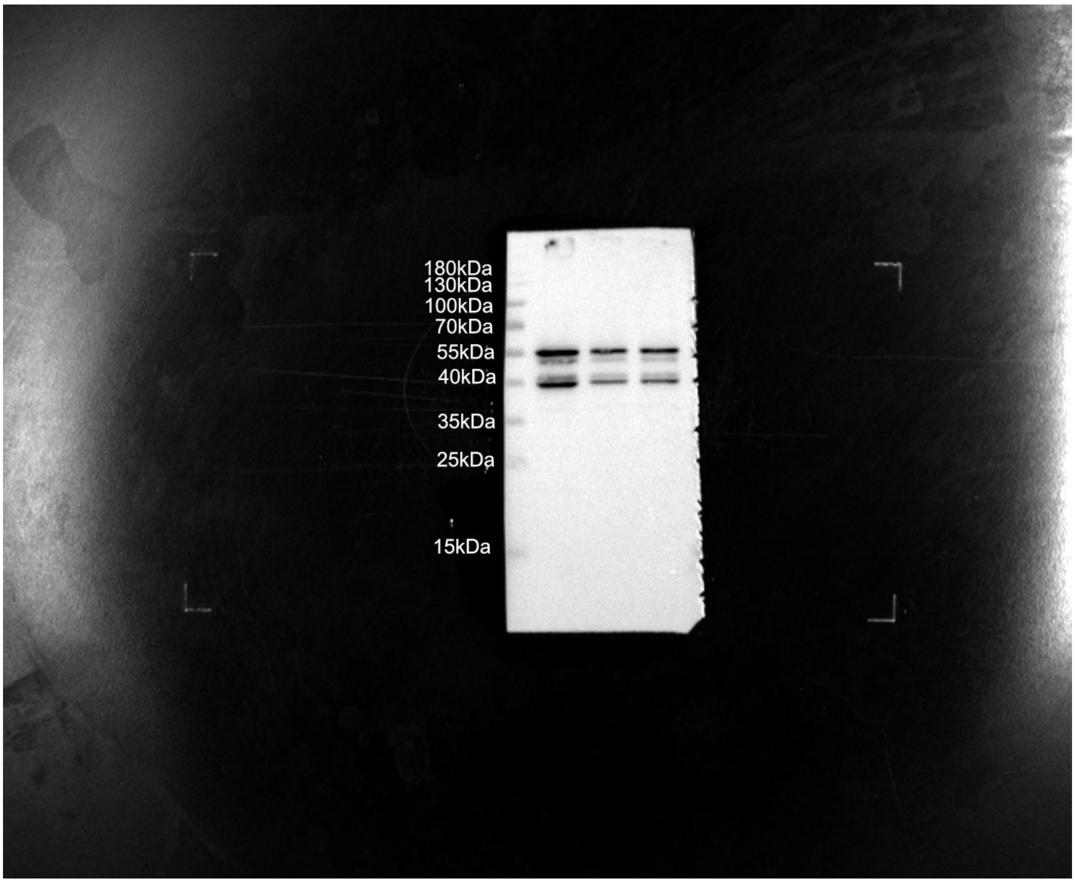

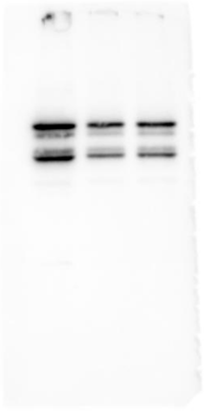

HK2

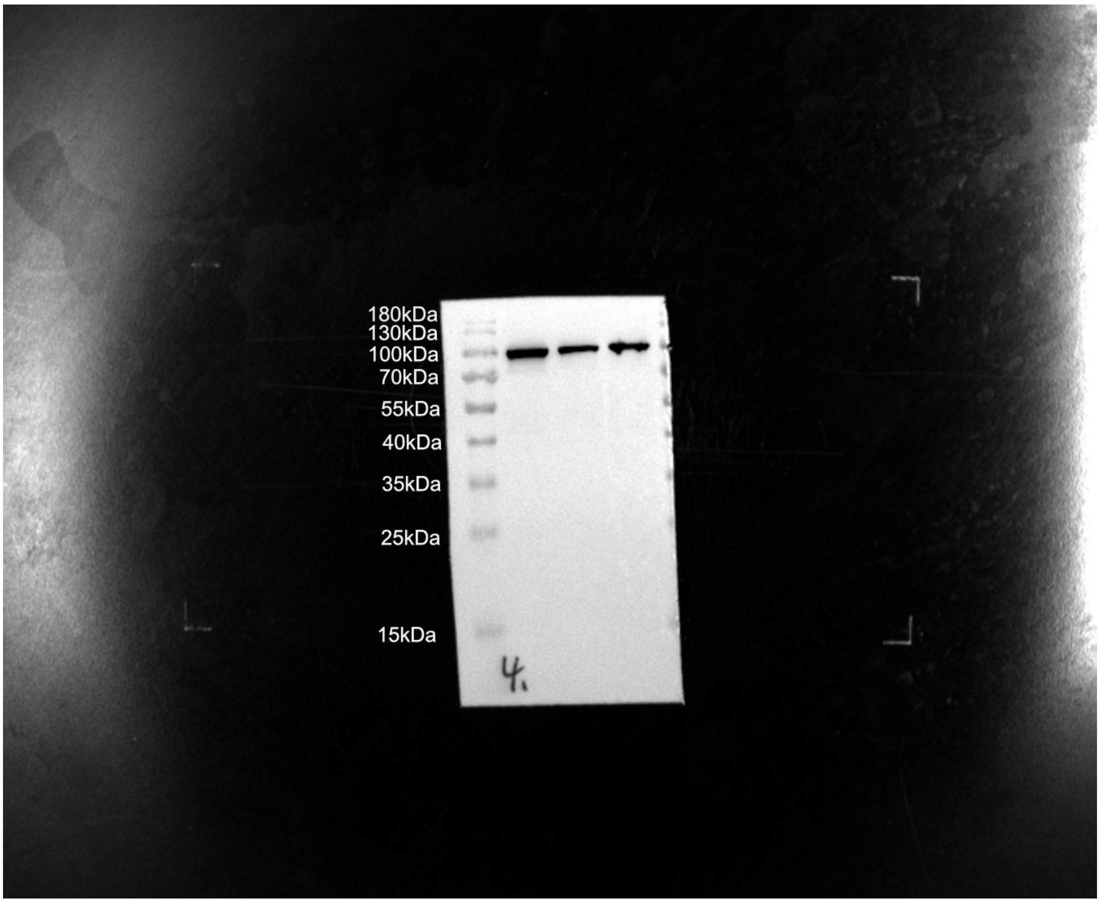

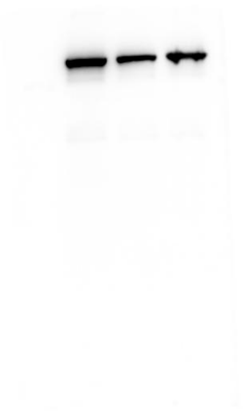

β-actin

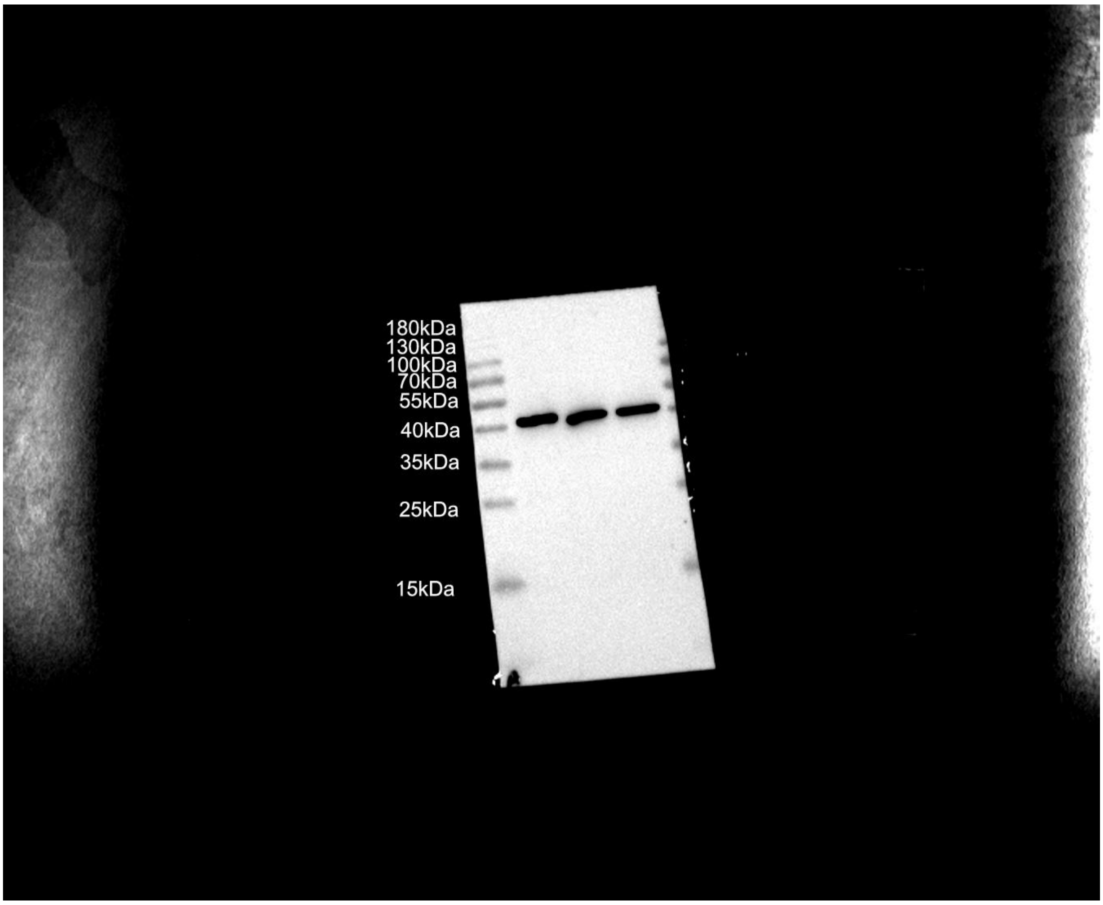

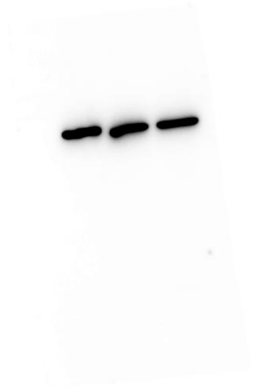

Fig4E HCT116 oe-RFC2

LDHA

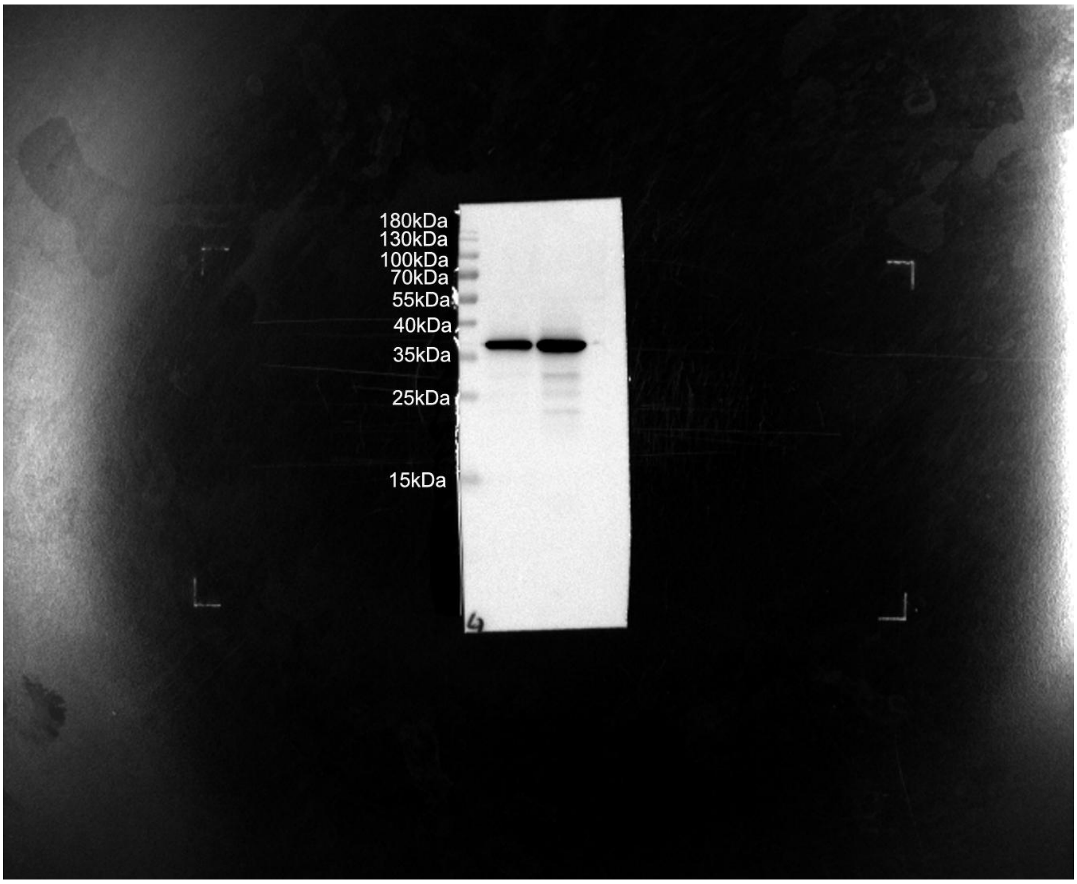

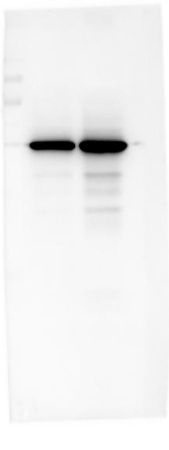

GLUT1

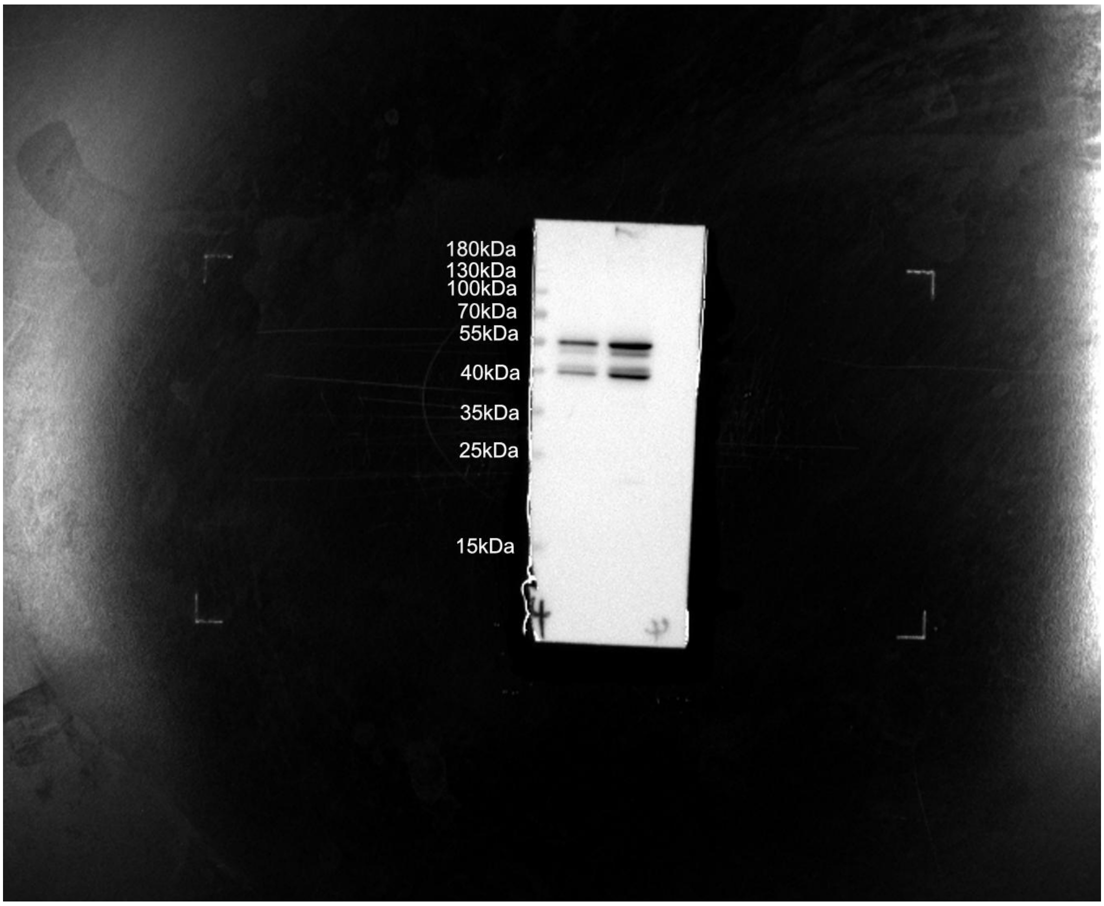

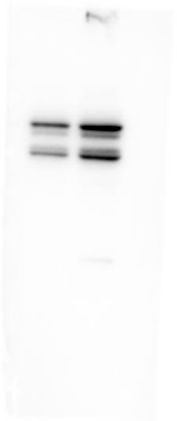

HK2

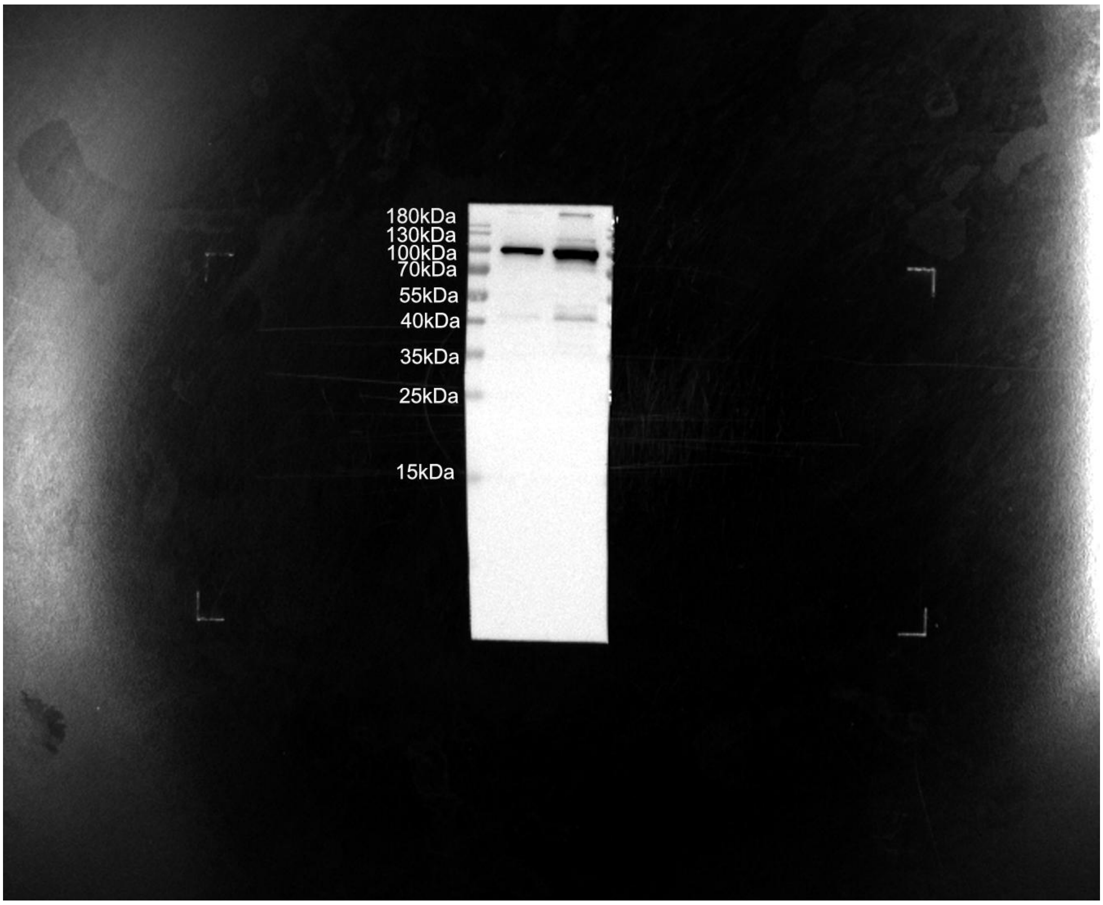

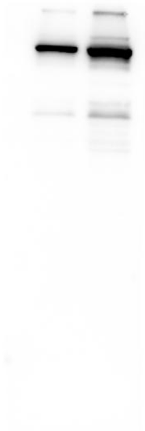

β-actin

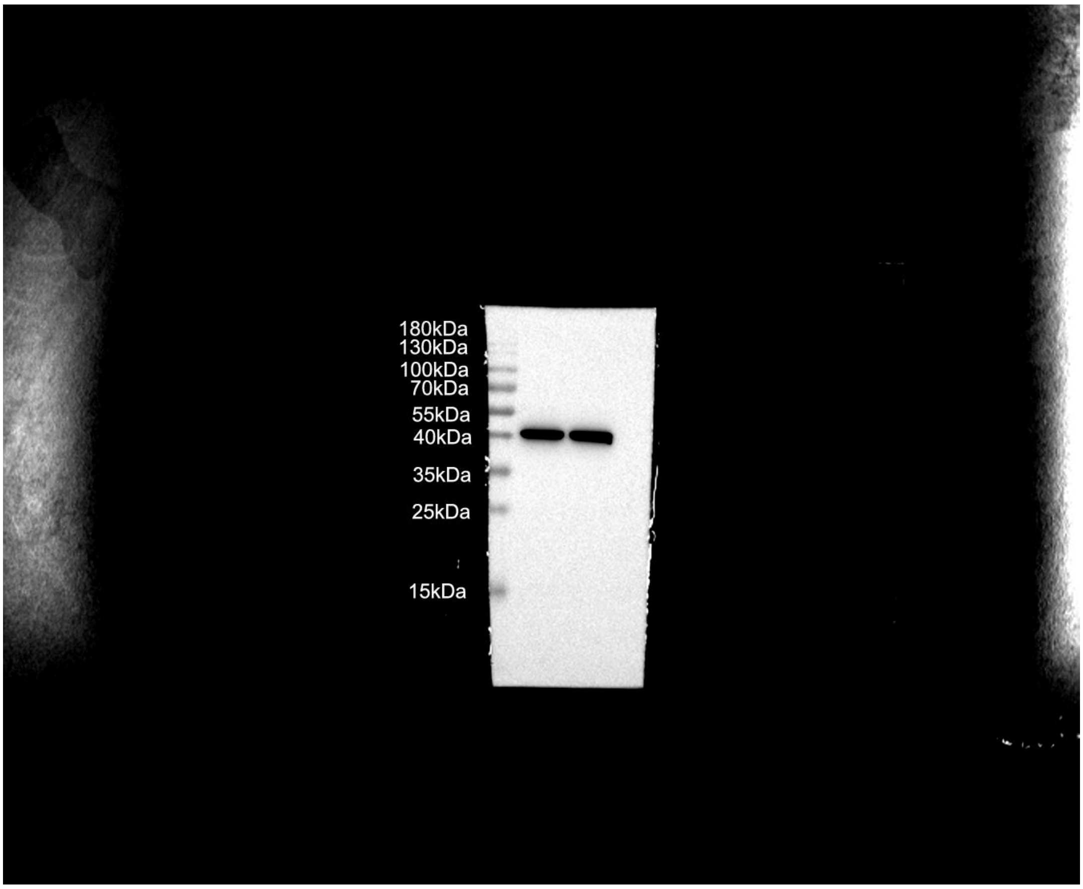

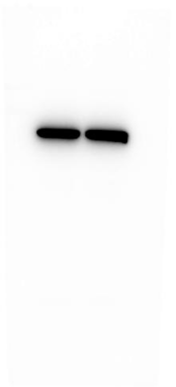

Fig4E SW480 oe-RFC2

LDHA

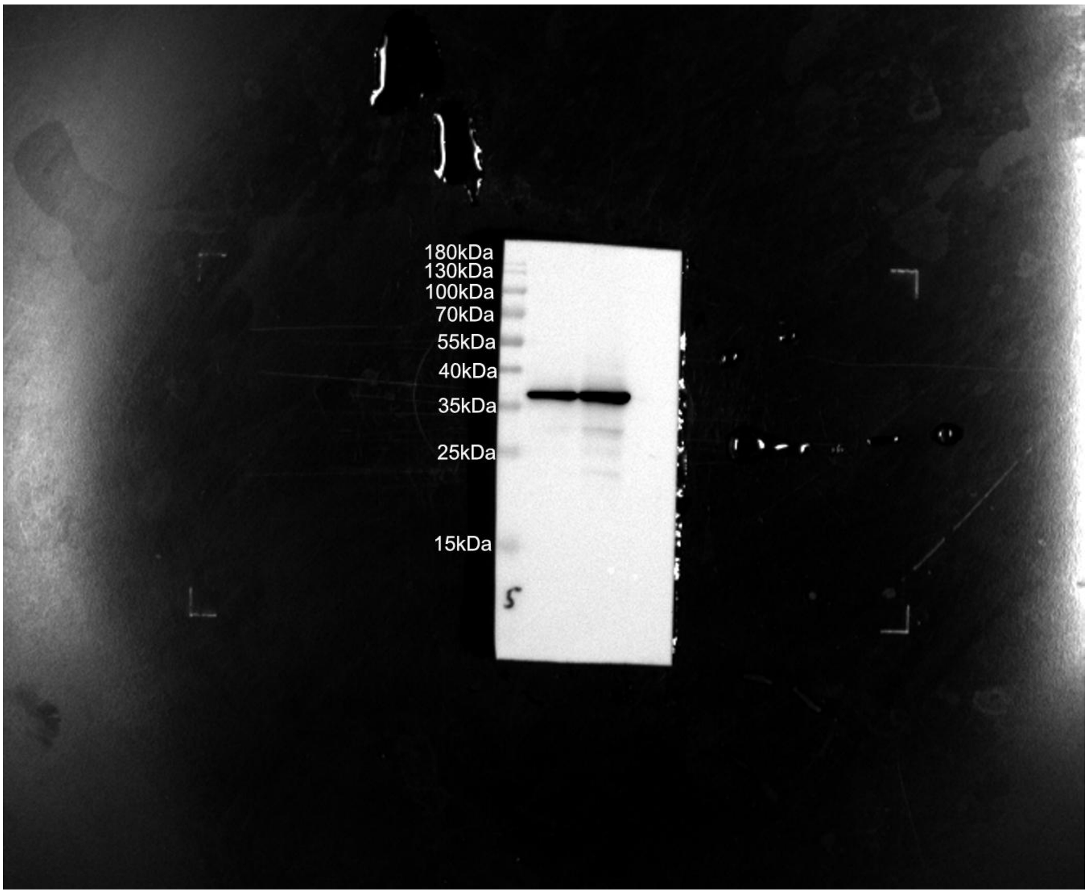

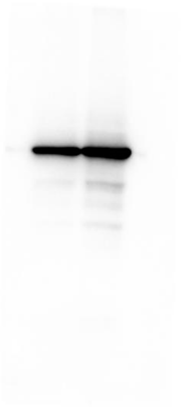

GLUT1

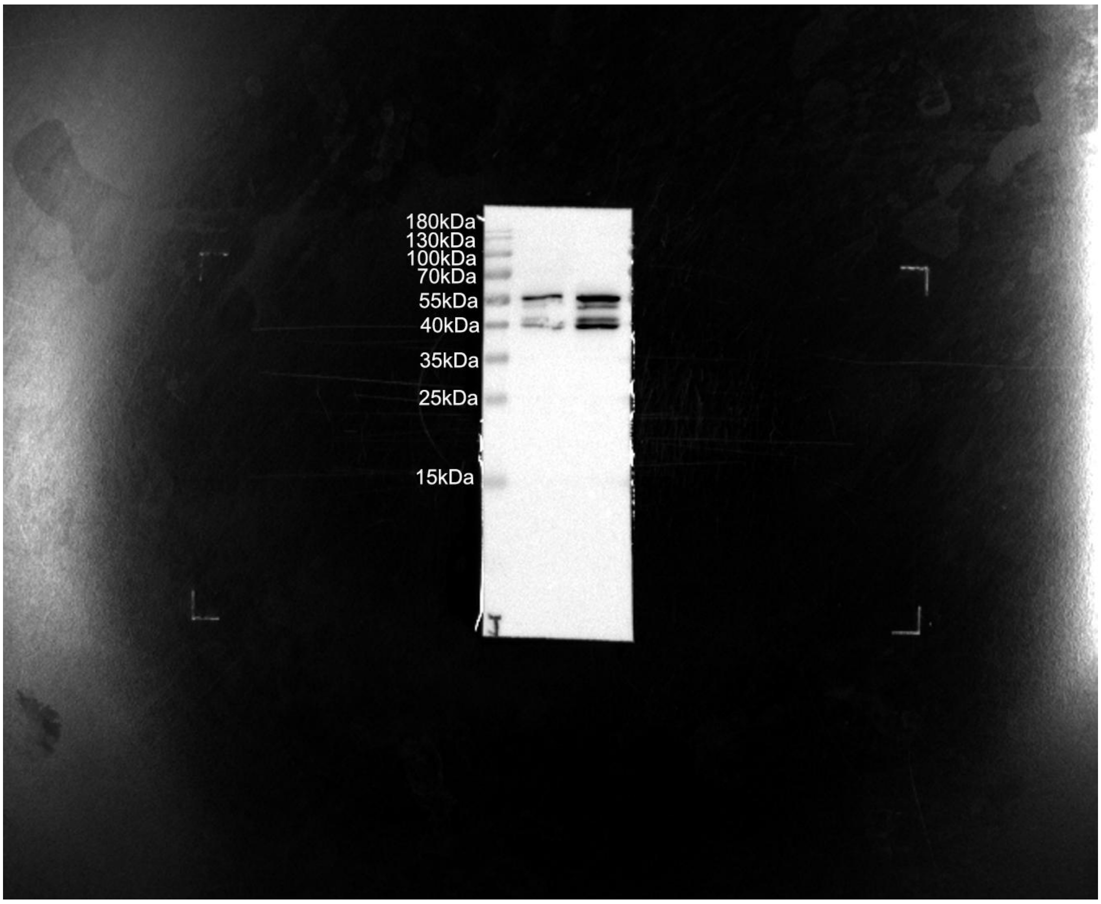

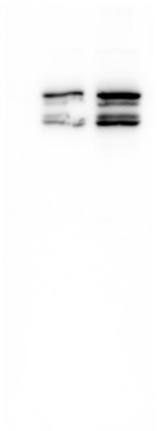

HK2

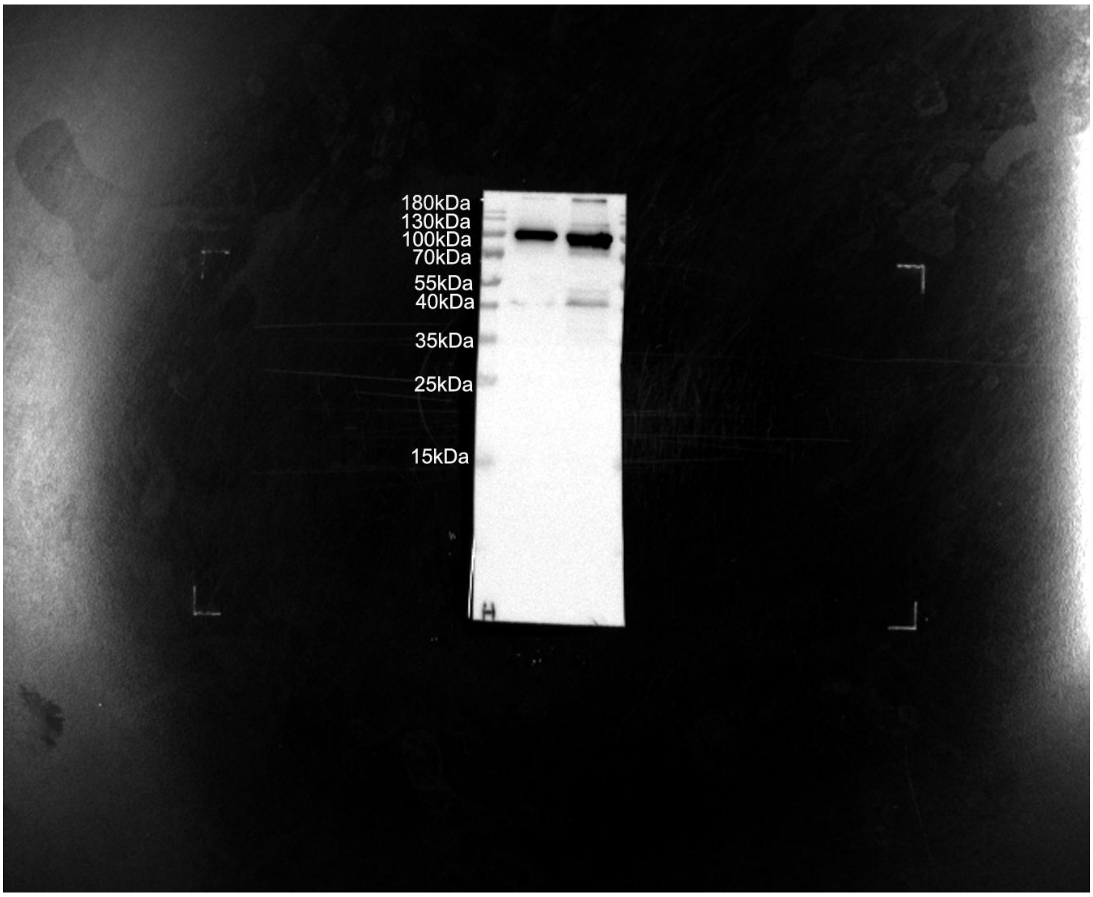

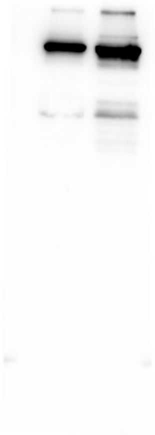

β-actin

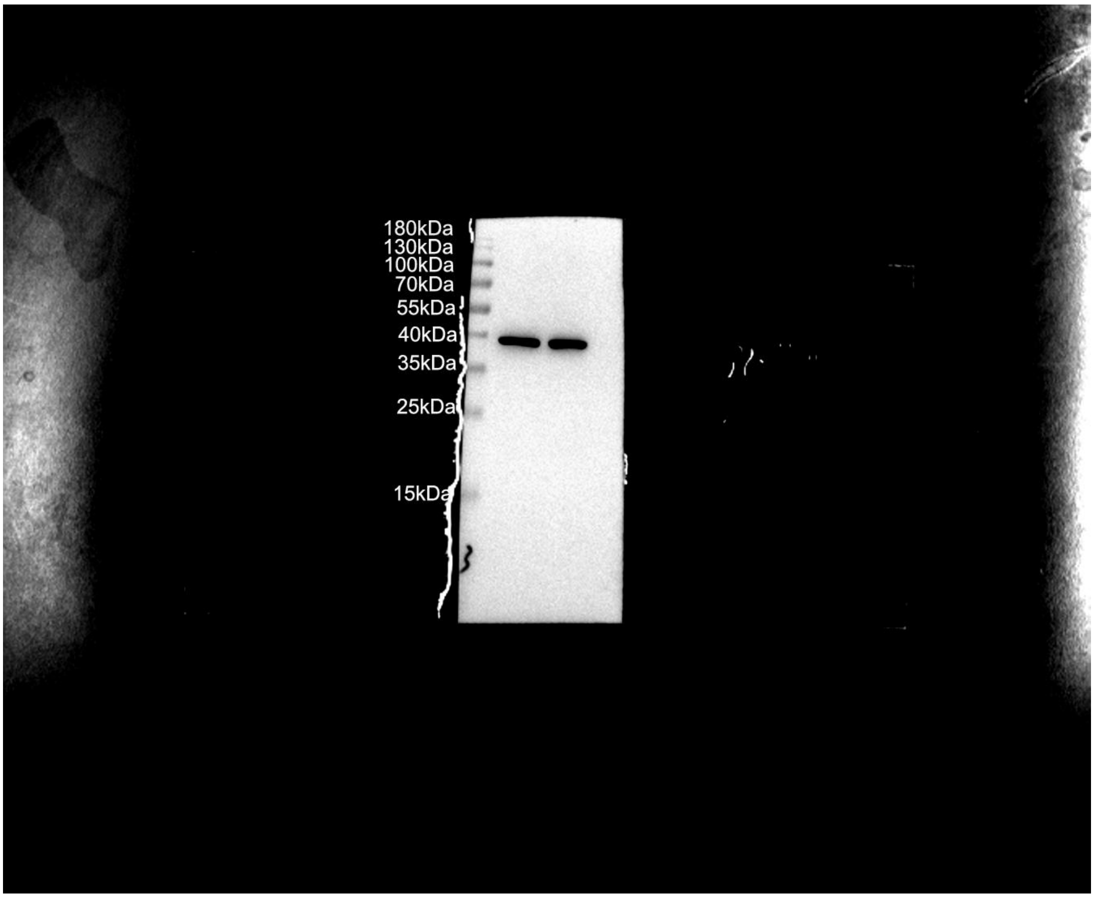

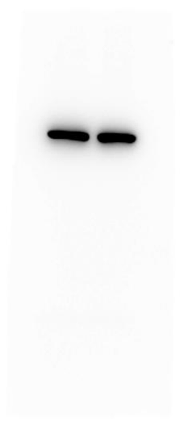

Fig5C HCT116 si-RFC2

MET

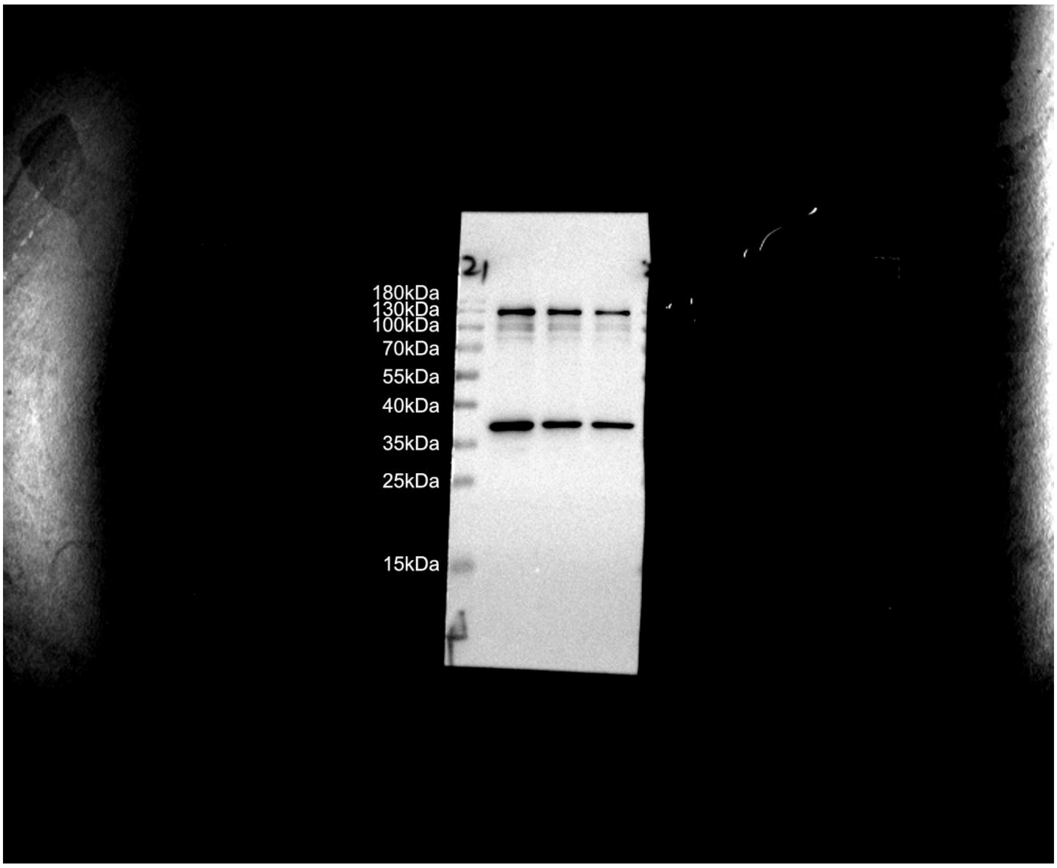

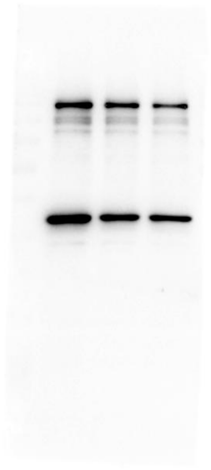

β-actin

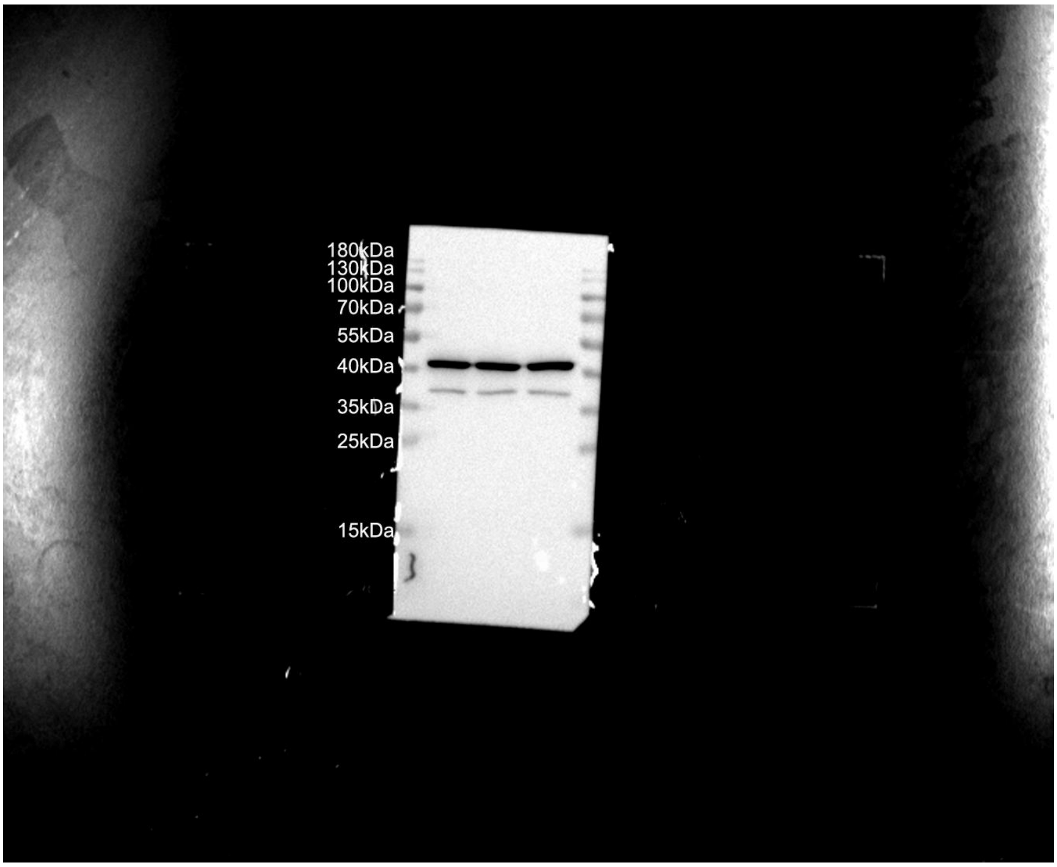

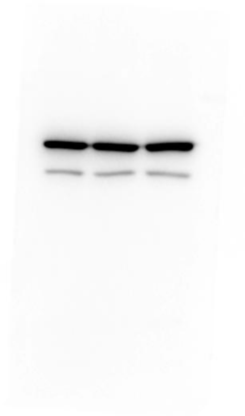

Fig5C SW480 si-RFC2

MET

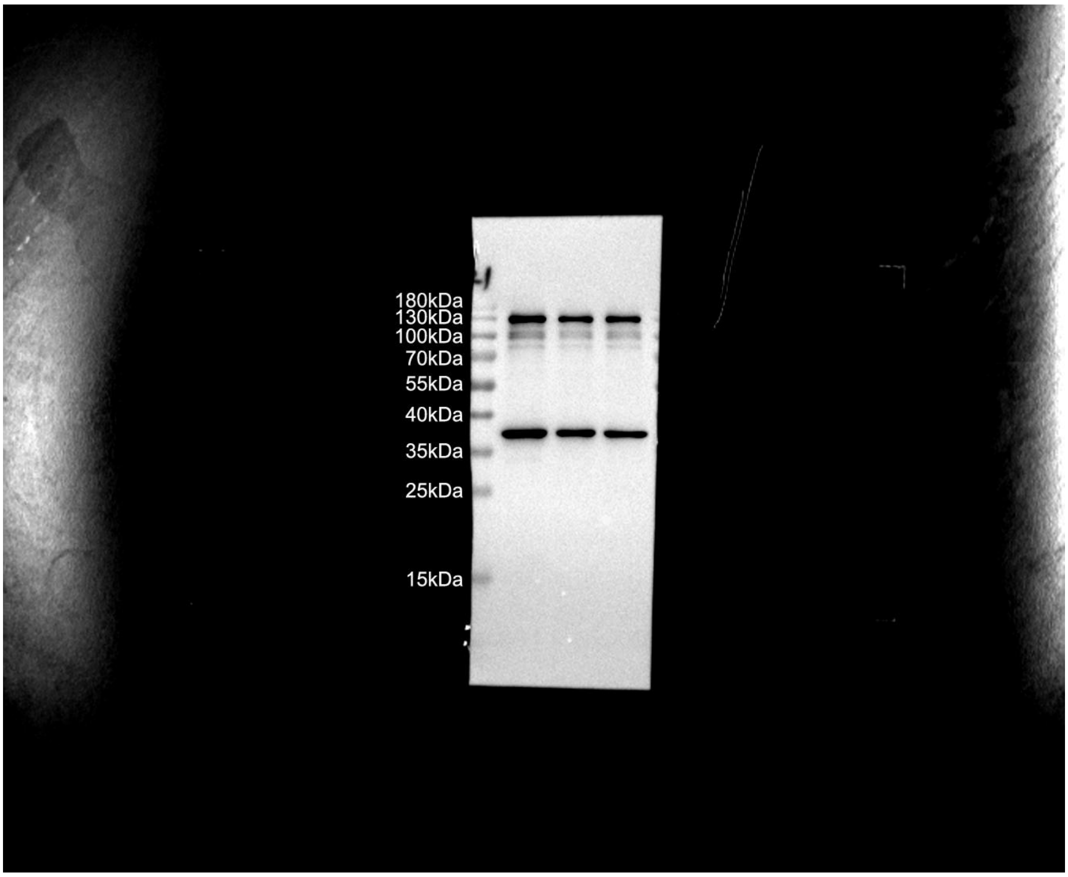

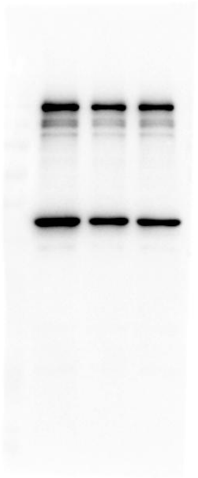

β-actin

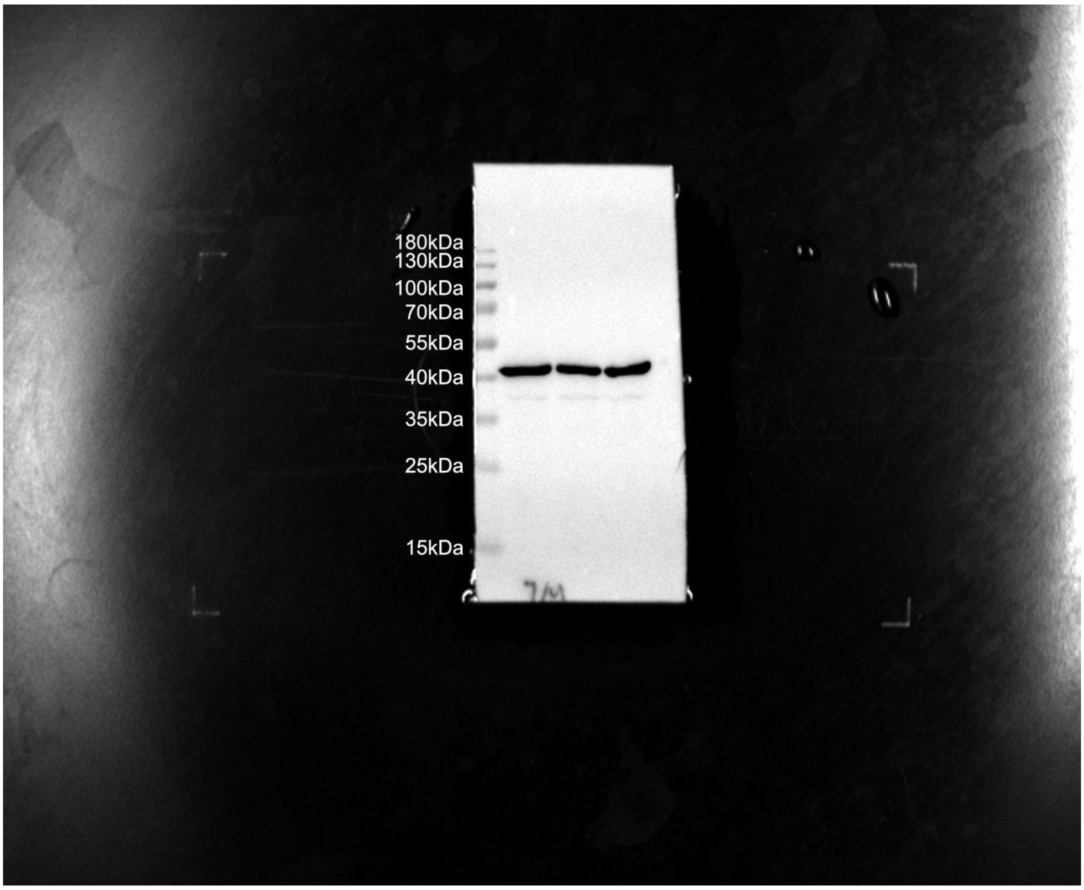

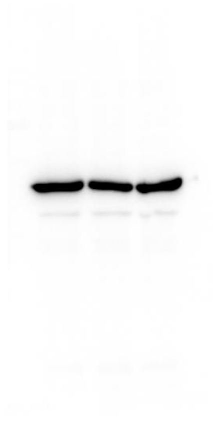

Fig5C HCT116 oe-RFC2

MET

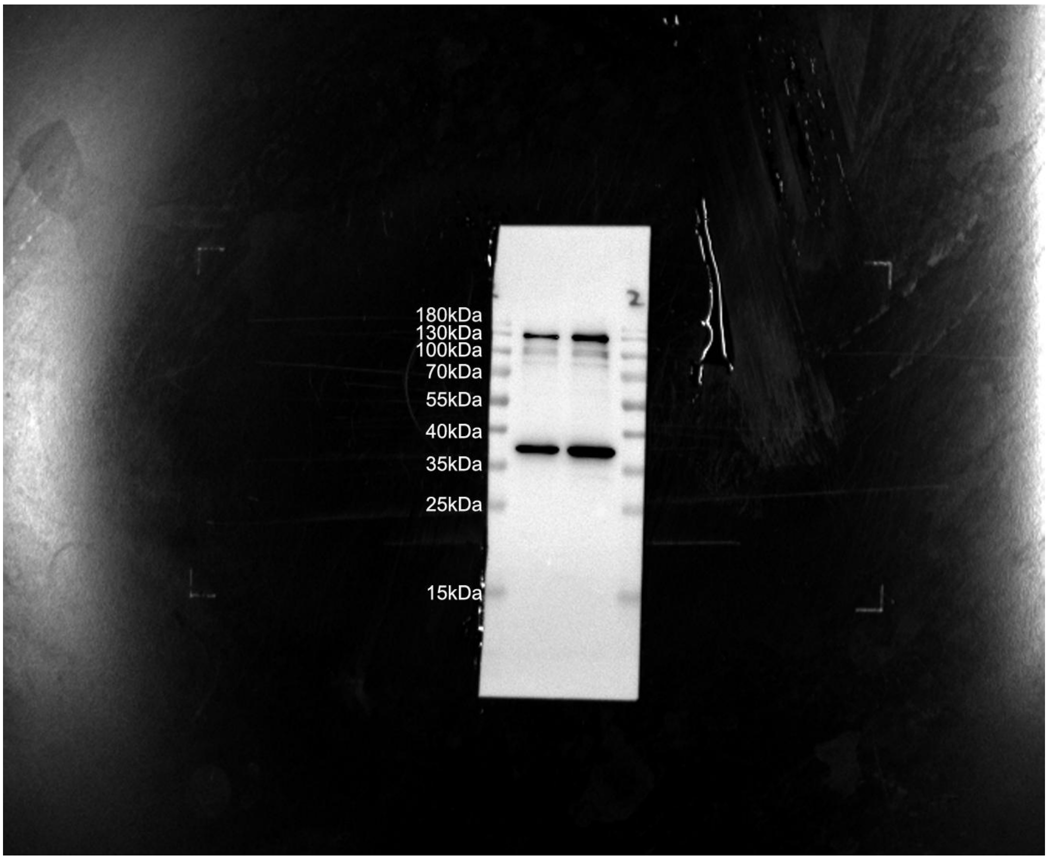

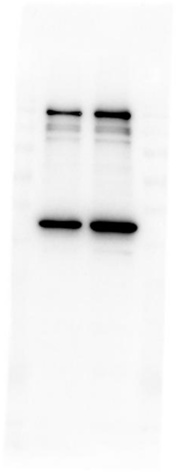

β-actin

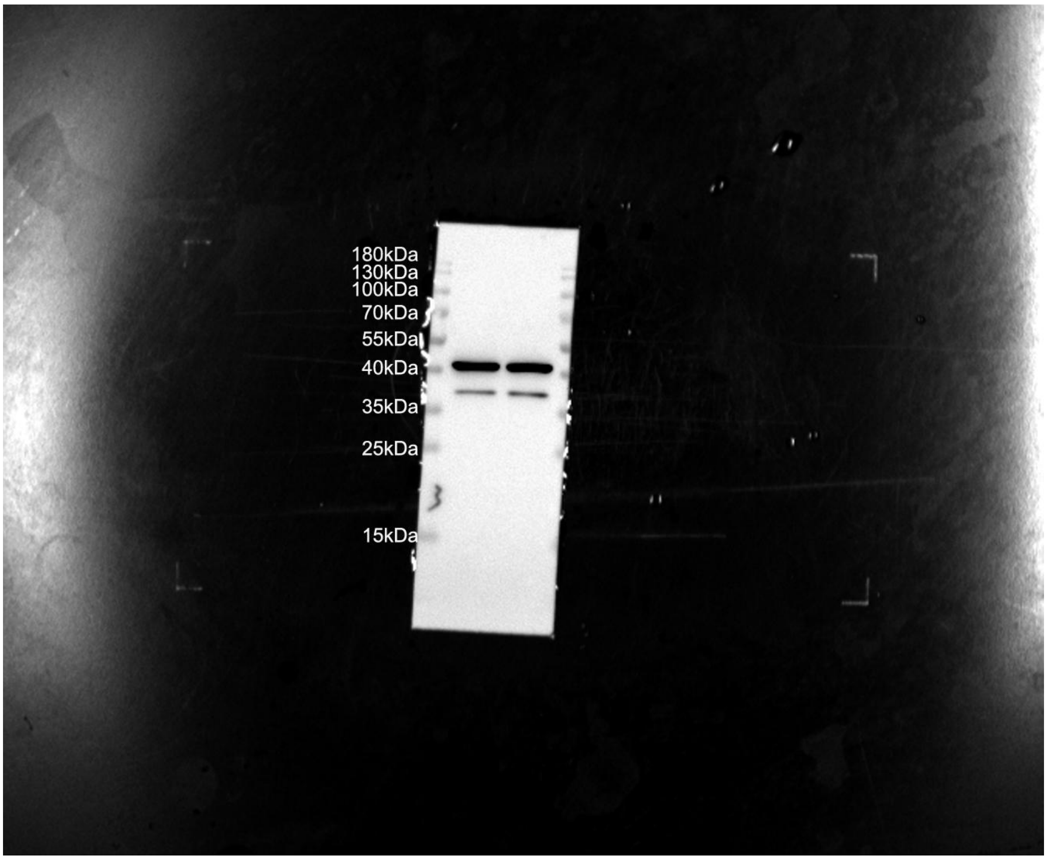

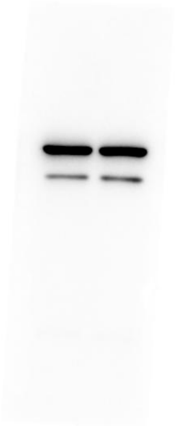

Fig5C SW480 oe-RFC2

MET

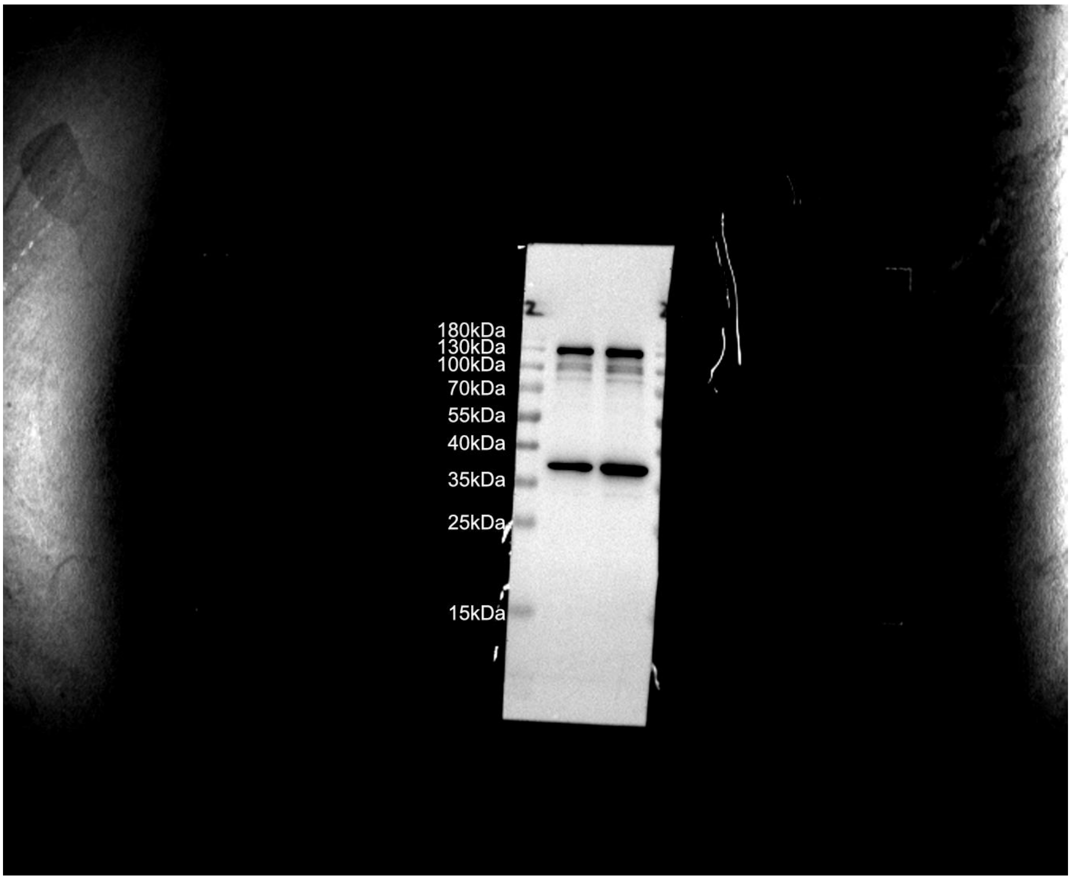

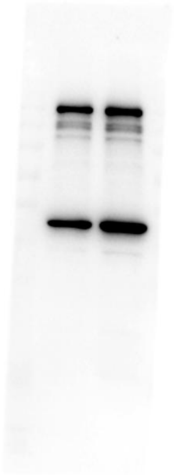

β-actin

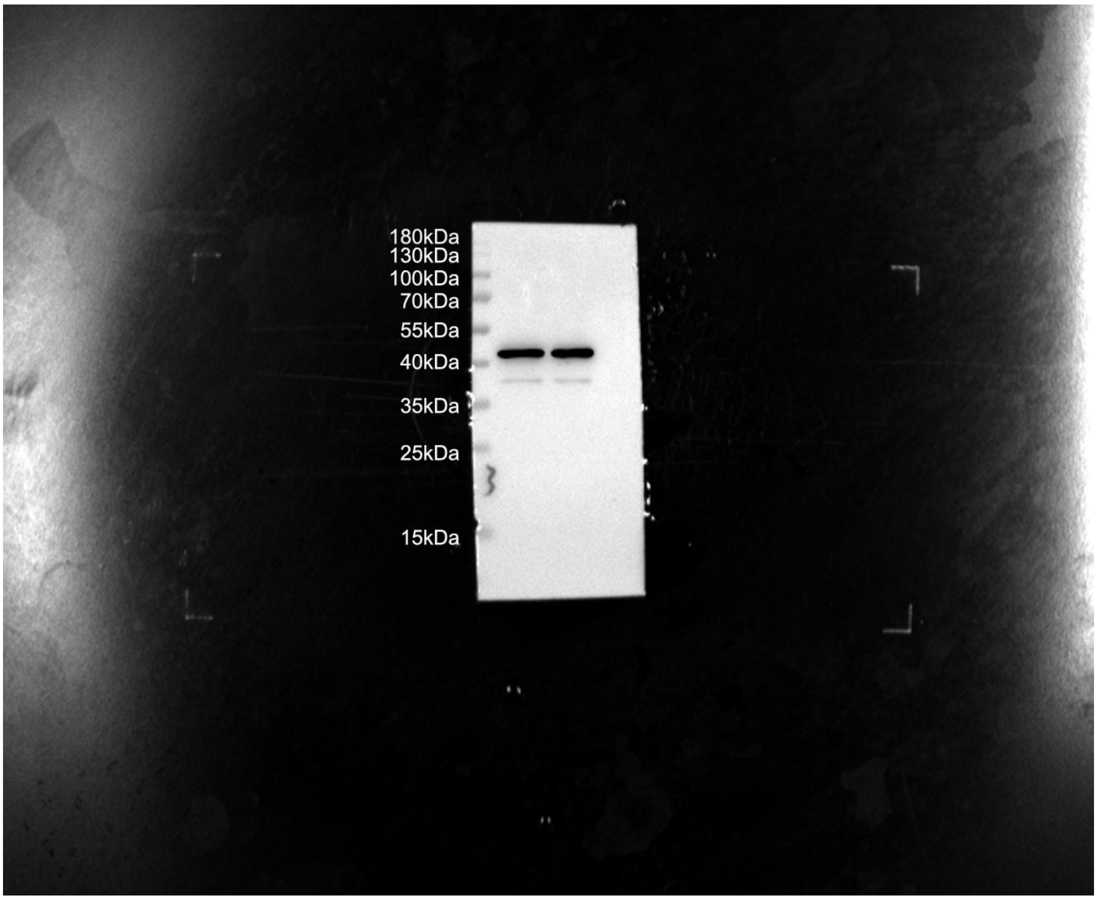

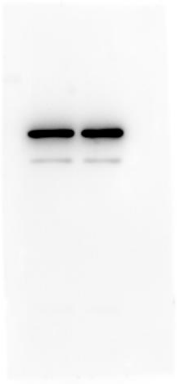

Fig5D HCT116 si-RFC2

p-PI3K

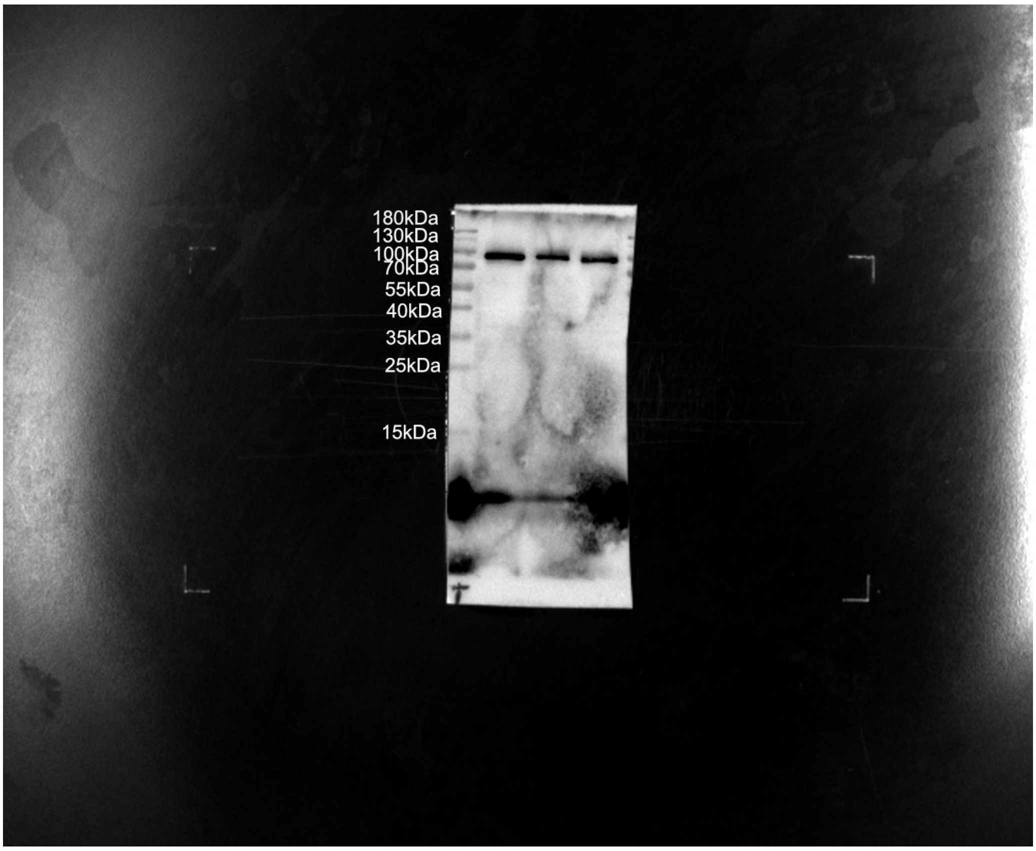

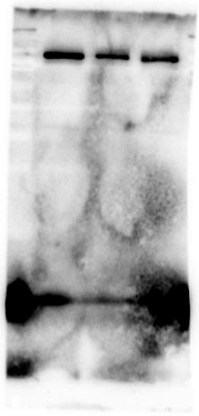

PI3K

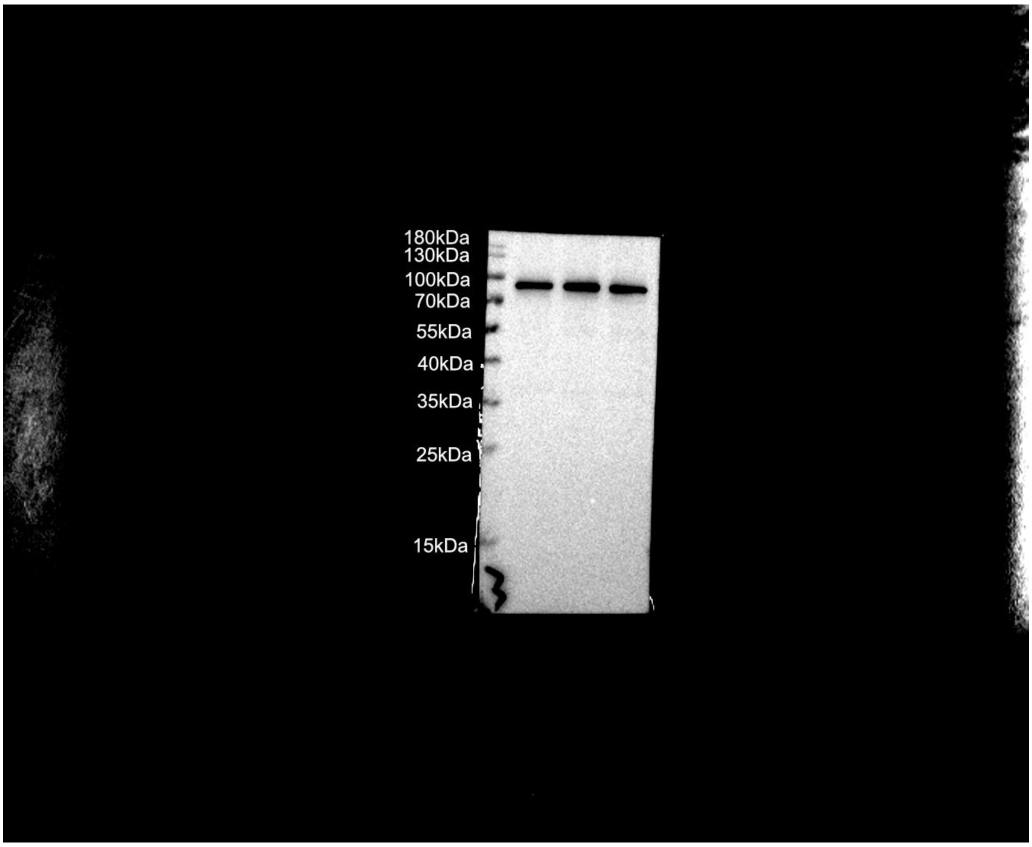

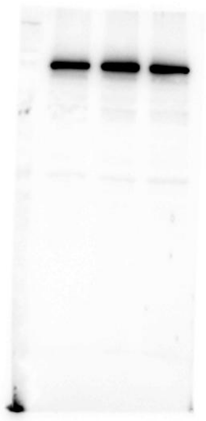

p-AKT

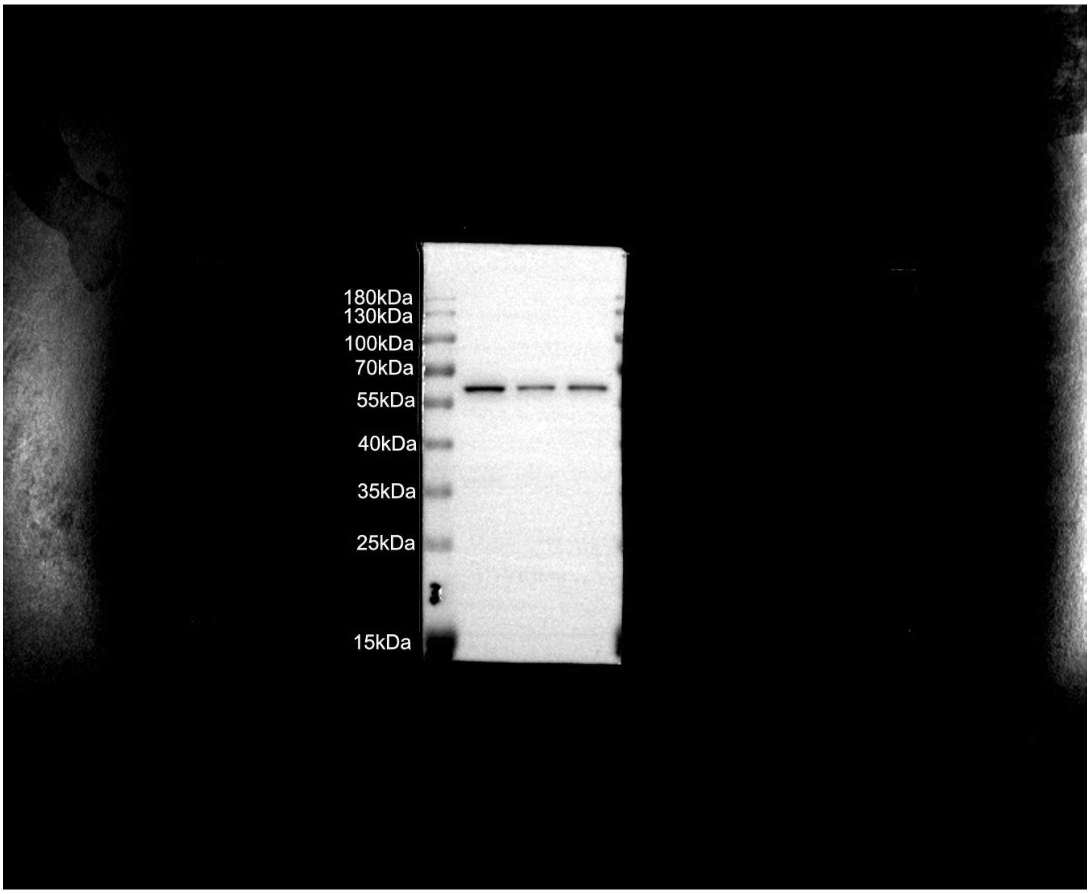

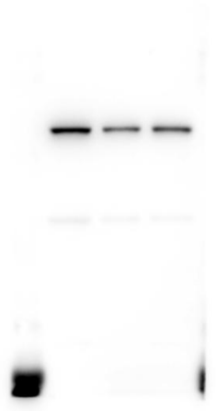

AKT

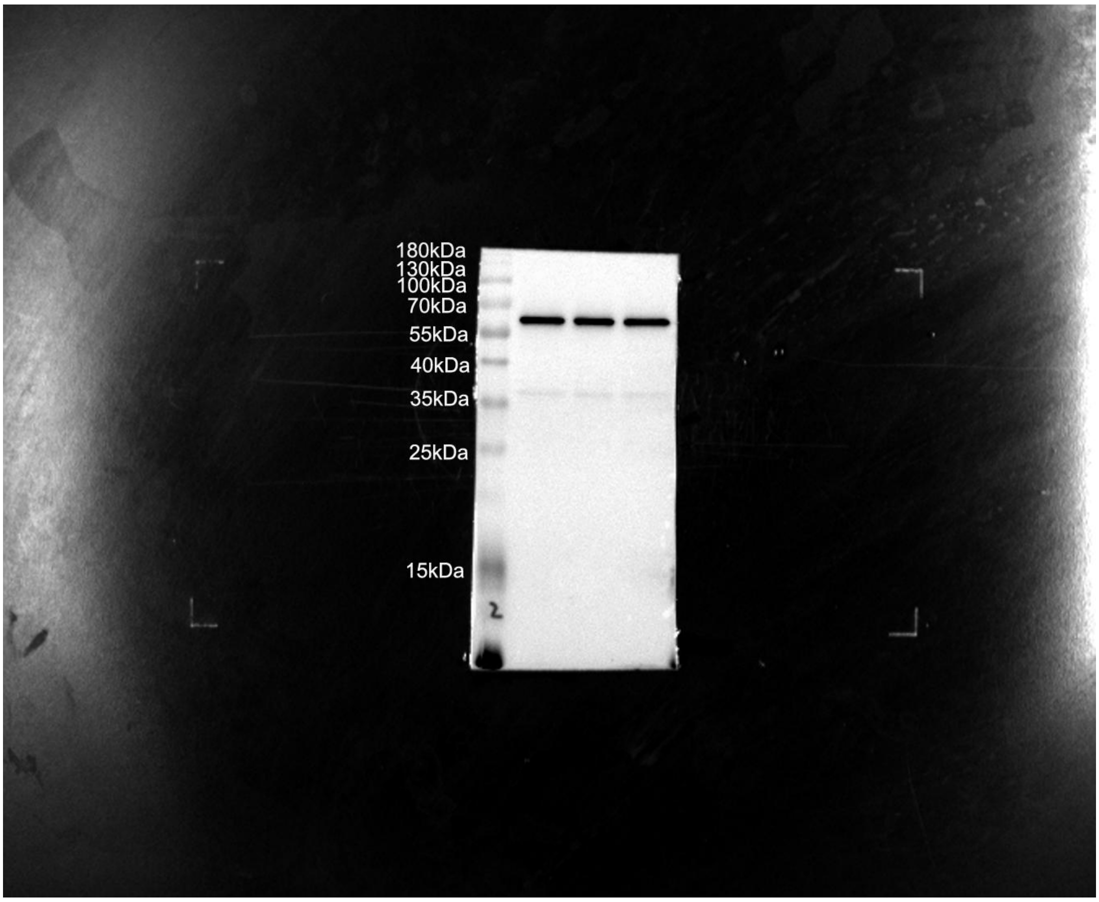

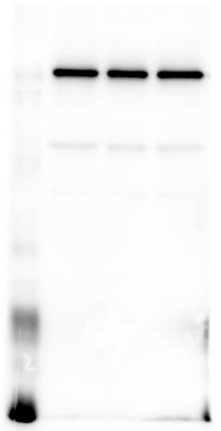

p-mTOR

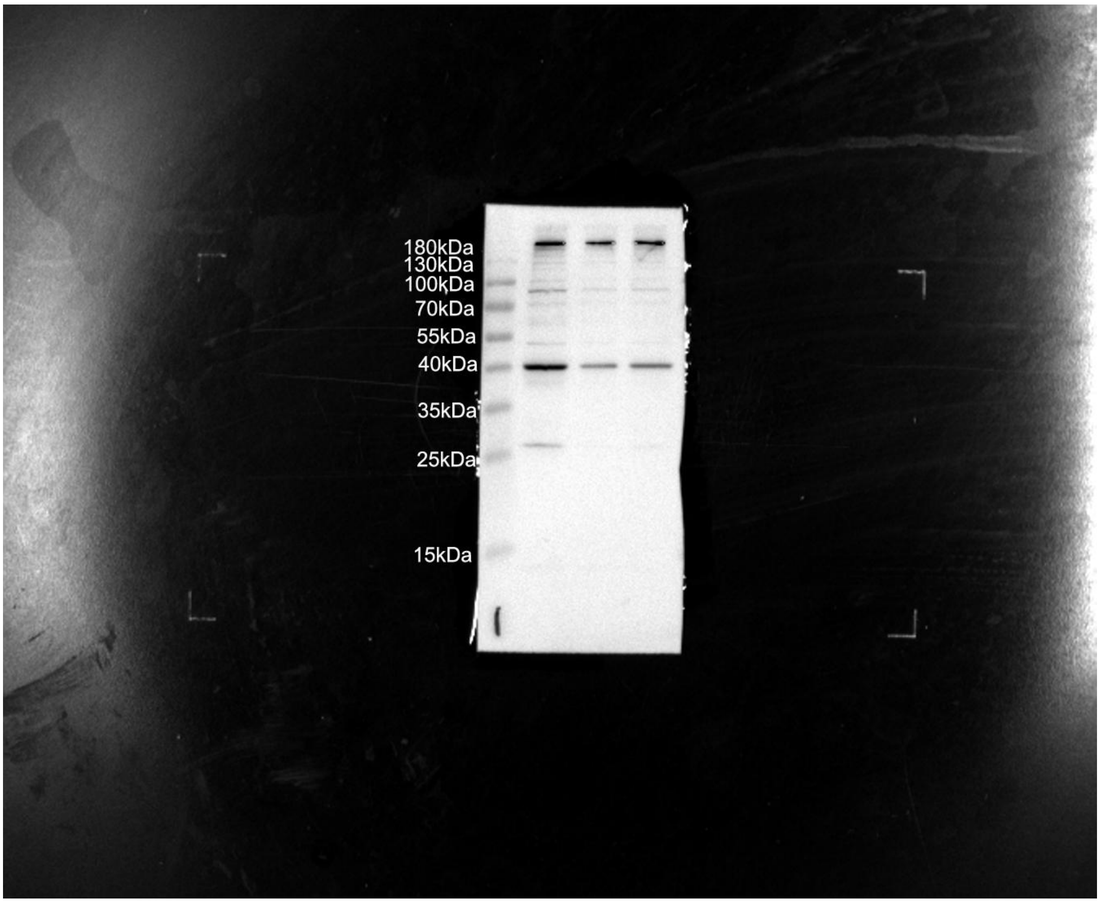

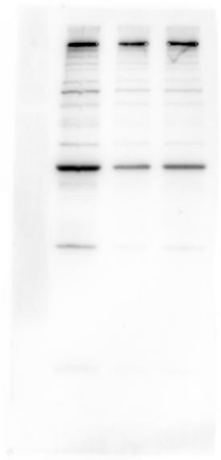

mTOR

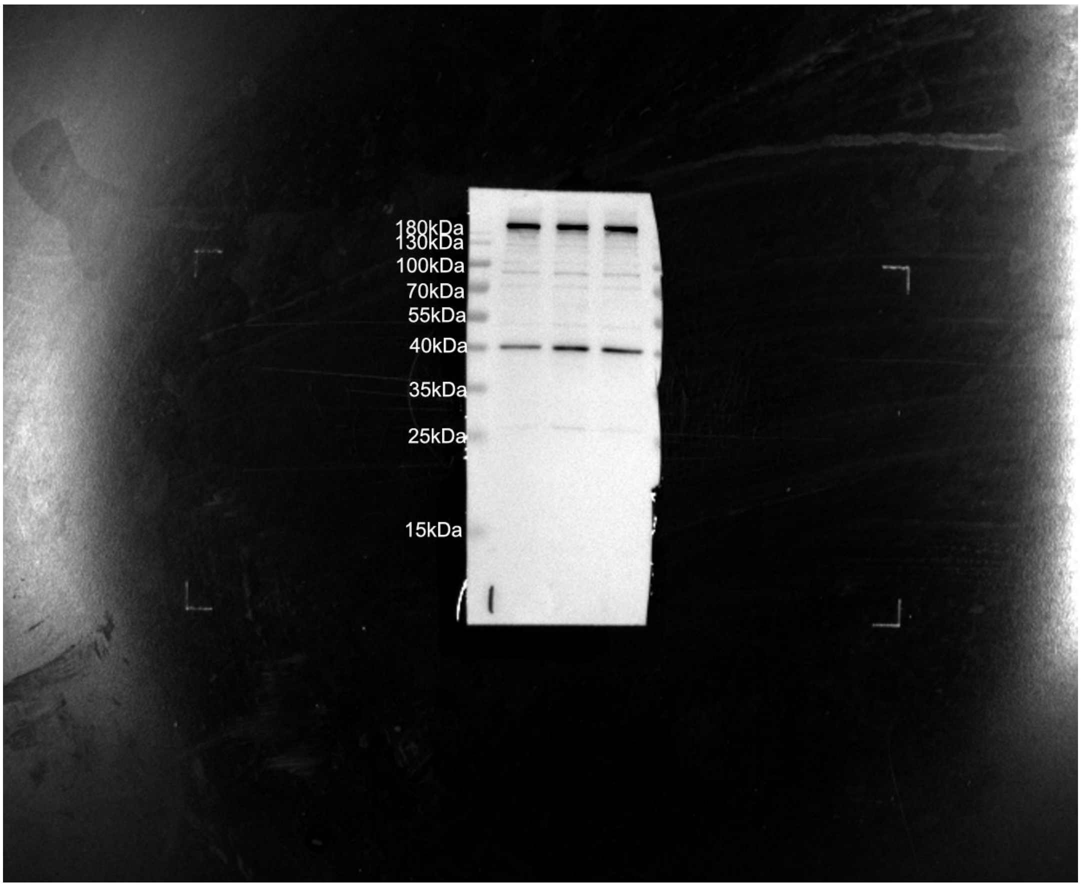

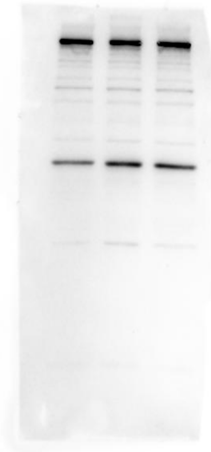

p-70S6K

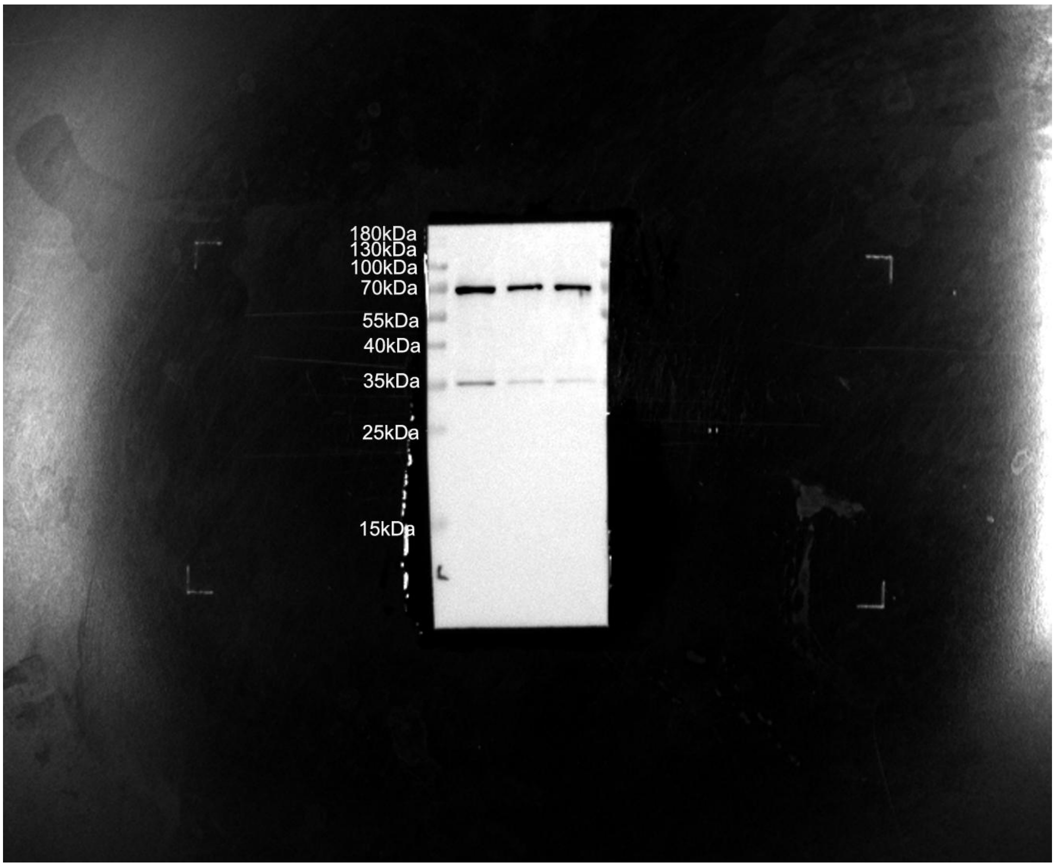

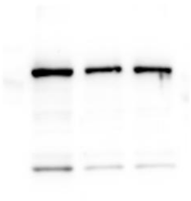

70S6K

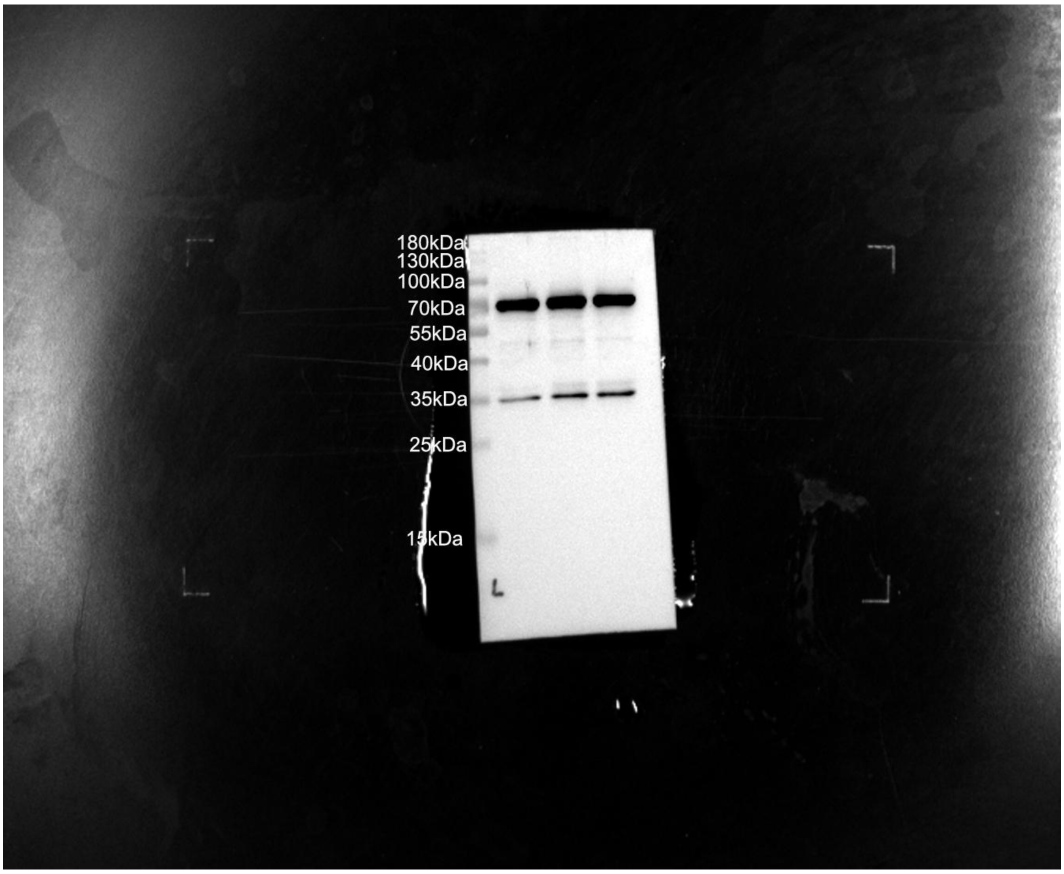

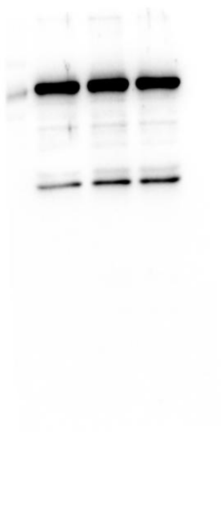

β-actin

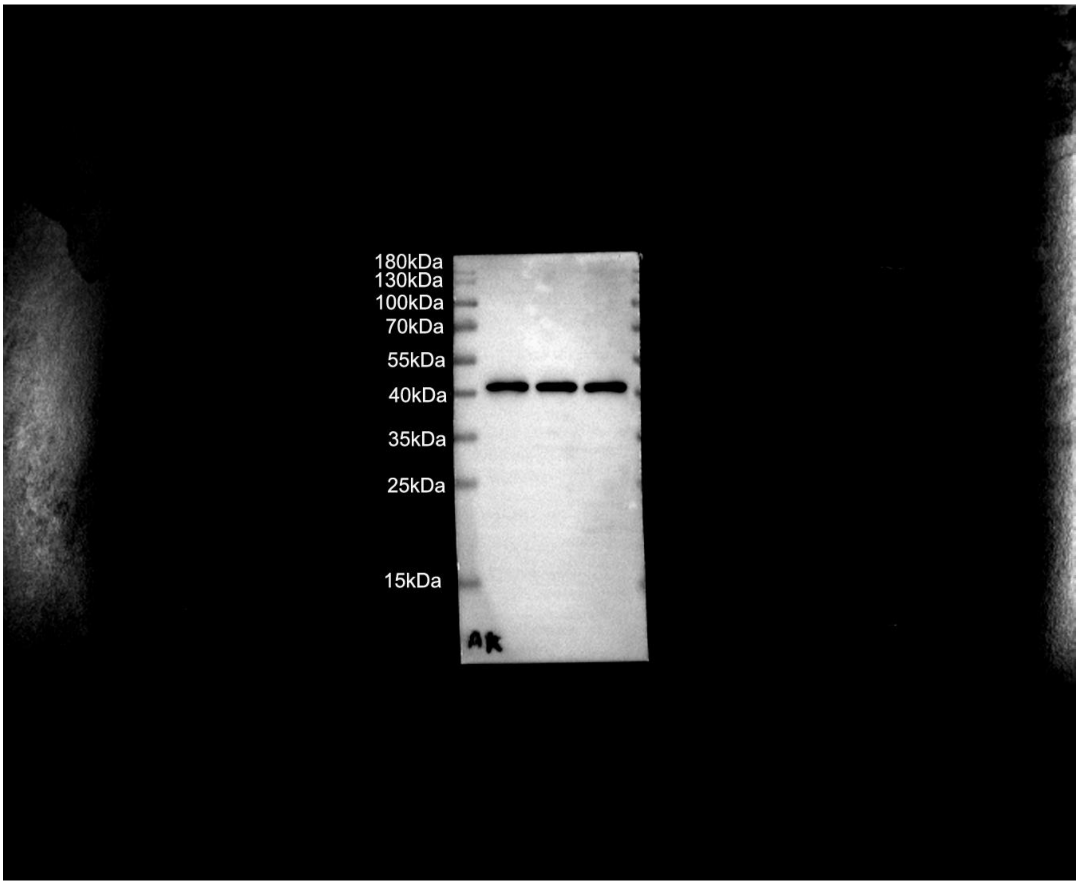
180kDa
130kDa
100kDa
70kDa
55kDa
40kDa
35kDa
25kDa
15kDa
AK

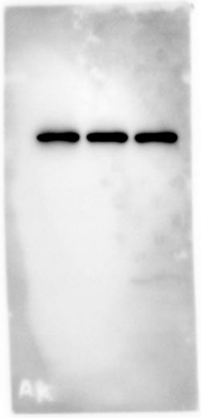

Fig5D SW480 si-RFC2

p-PI3K

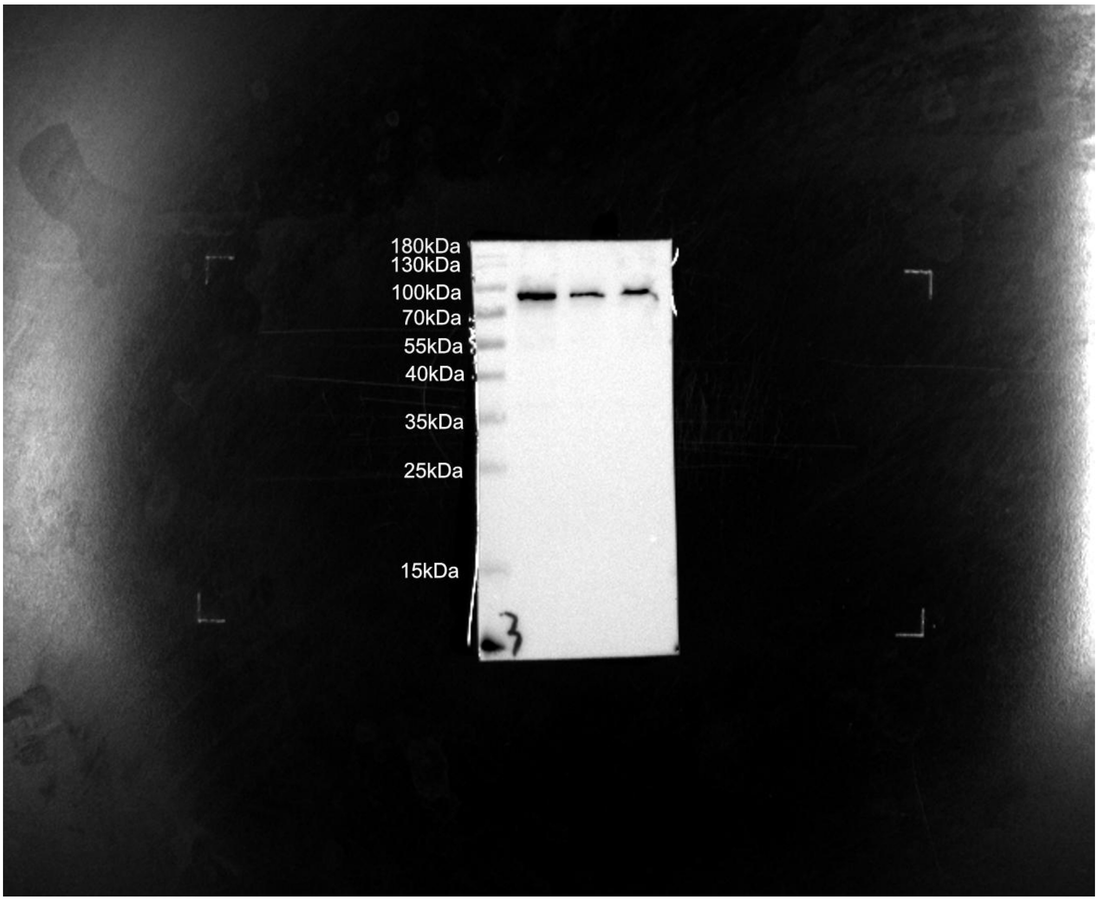

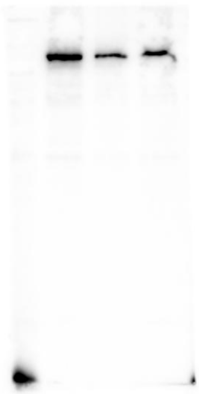

PI3K

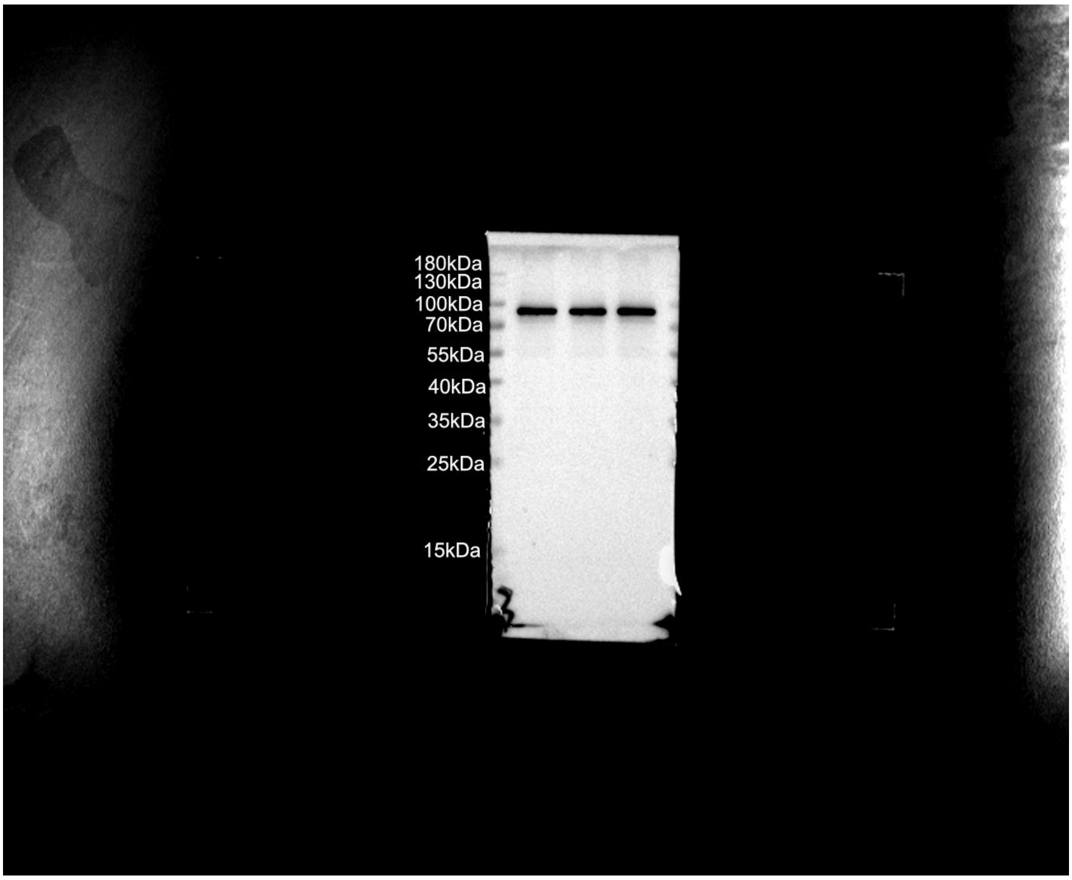

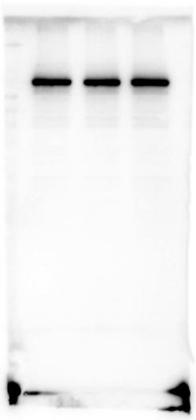

p-AKT

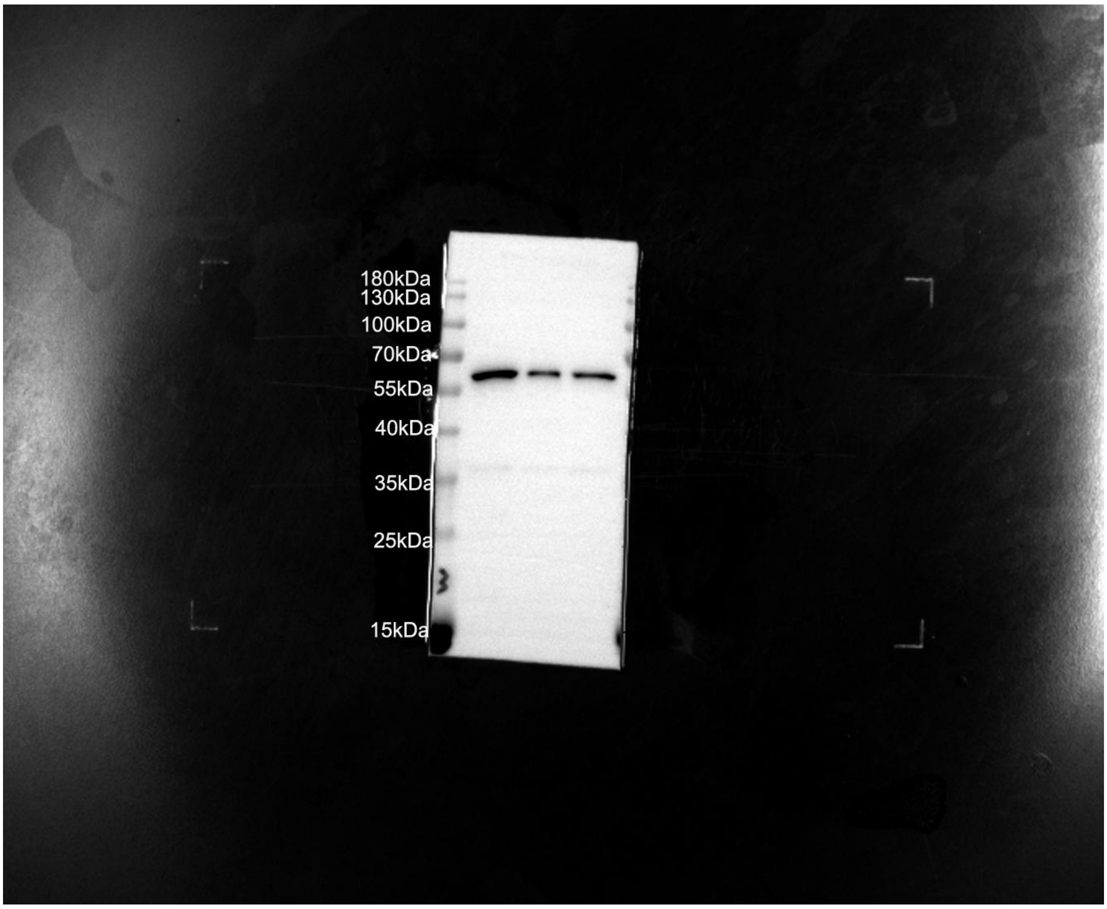

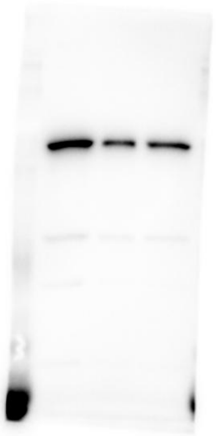

AKT

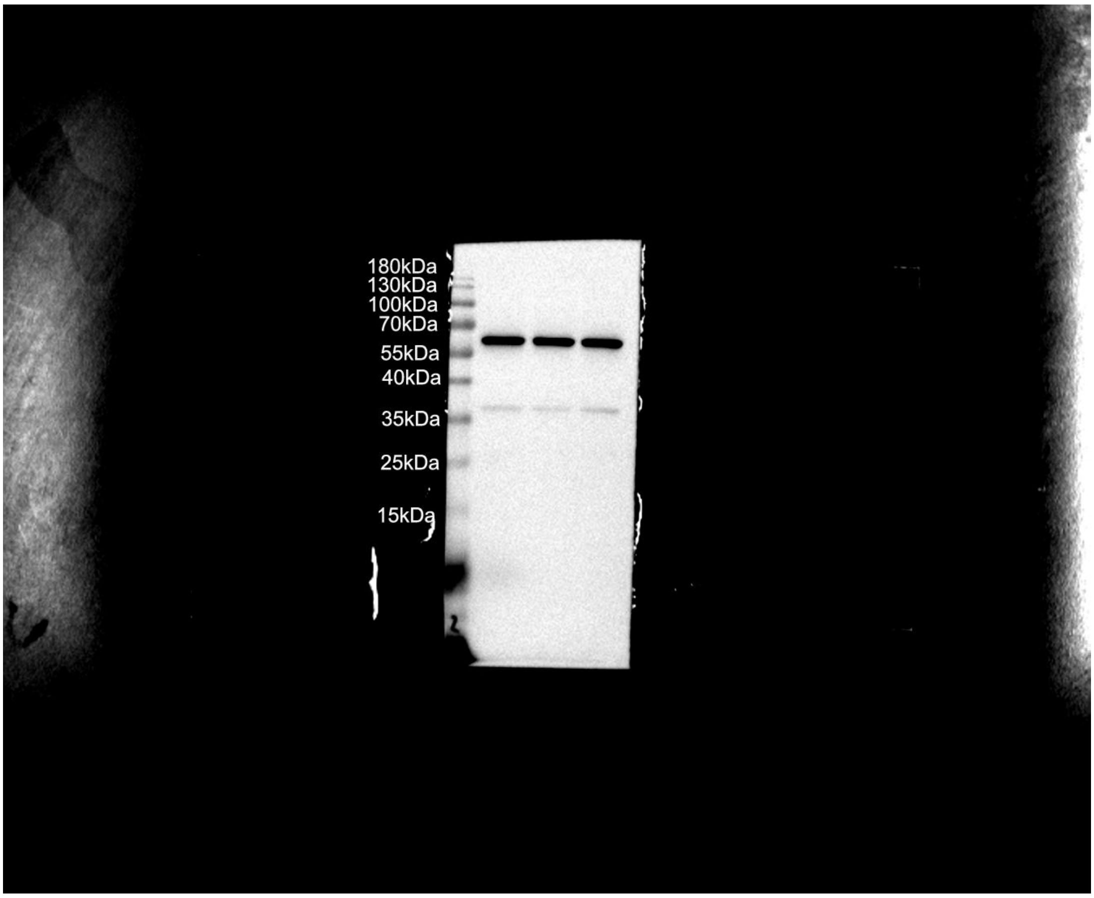

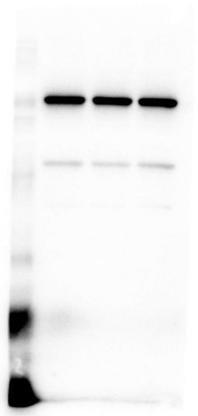

p-mTOR

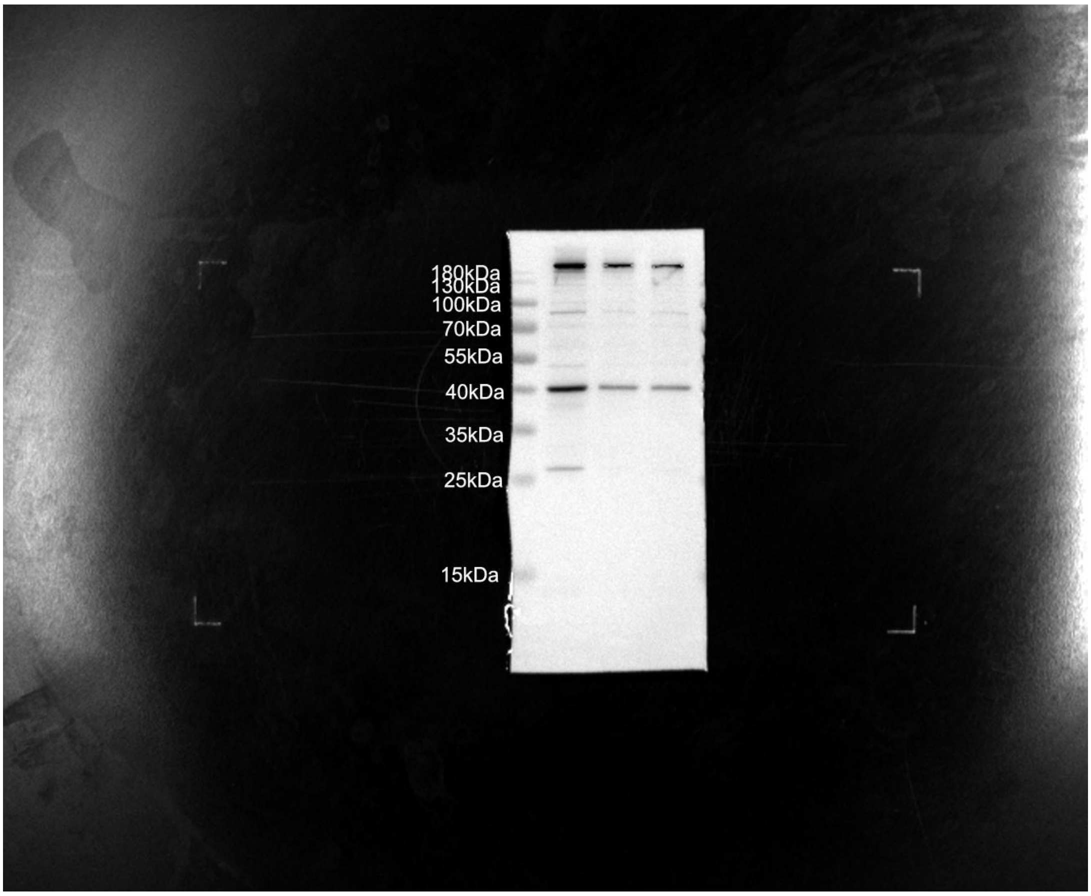

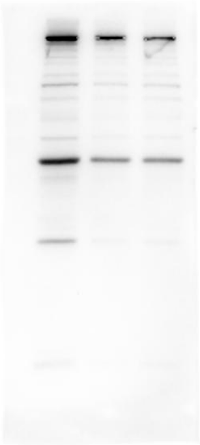

mTOR

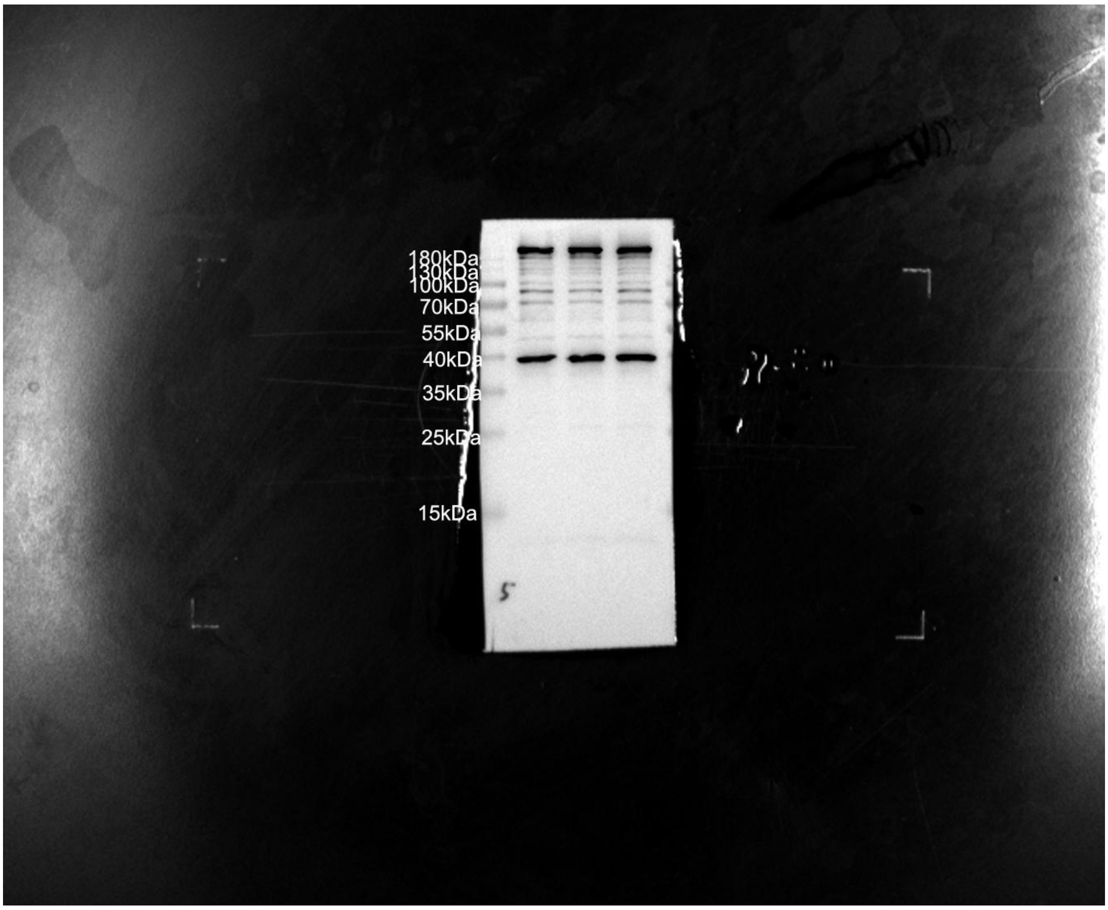

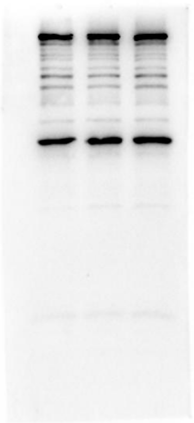

p-70S6K

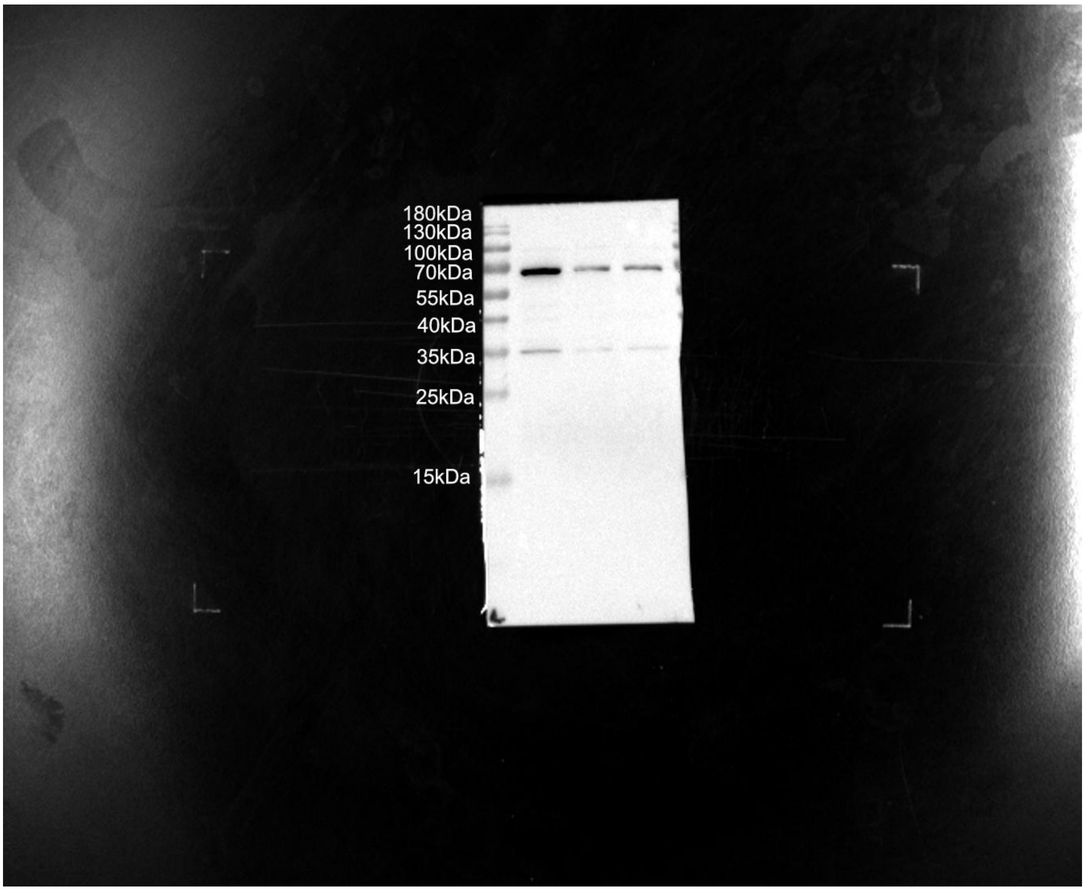

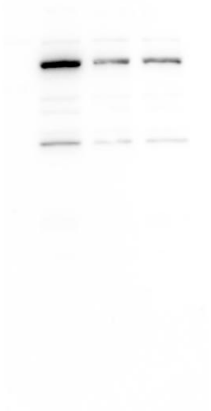

70S6K

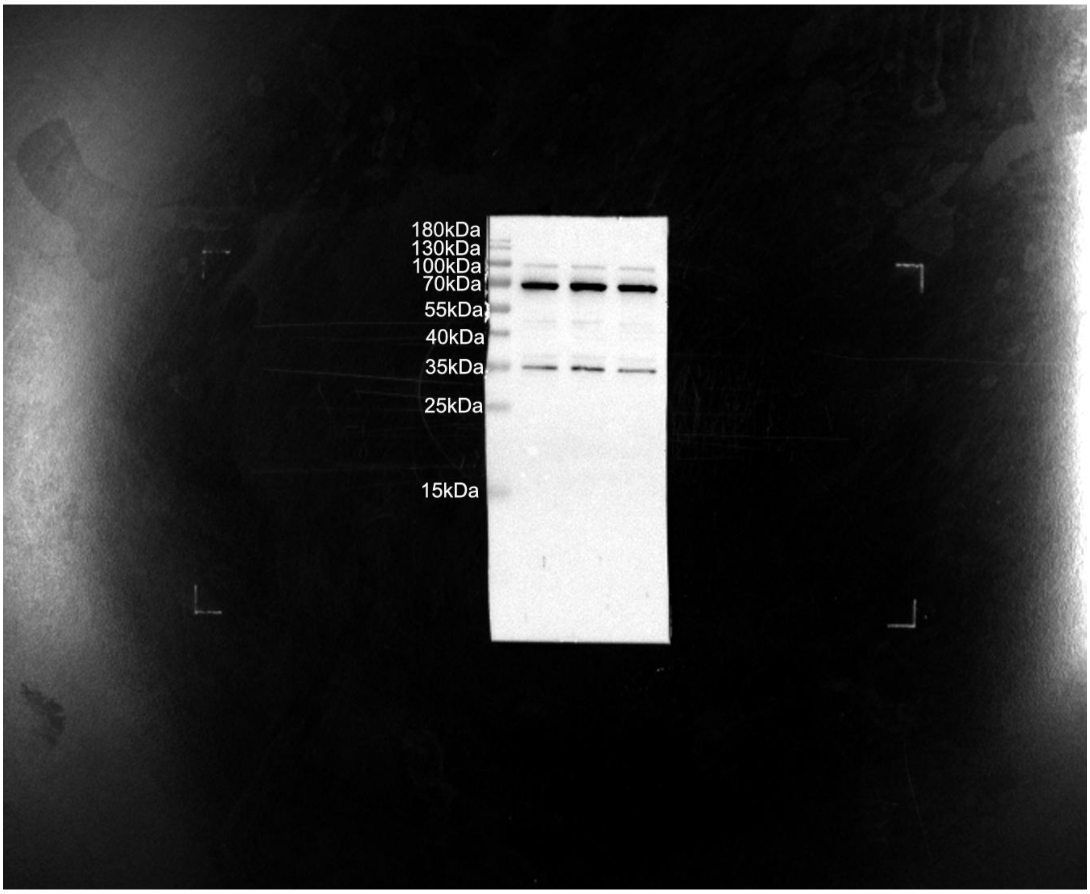

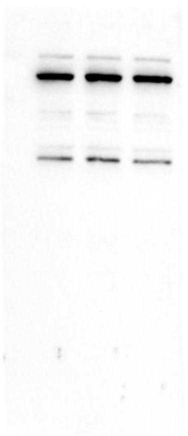

β-actin

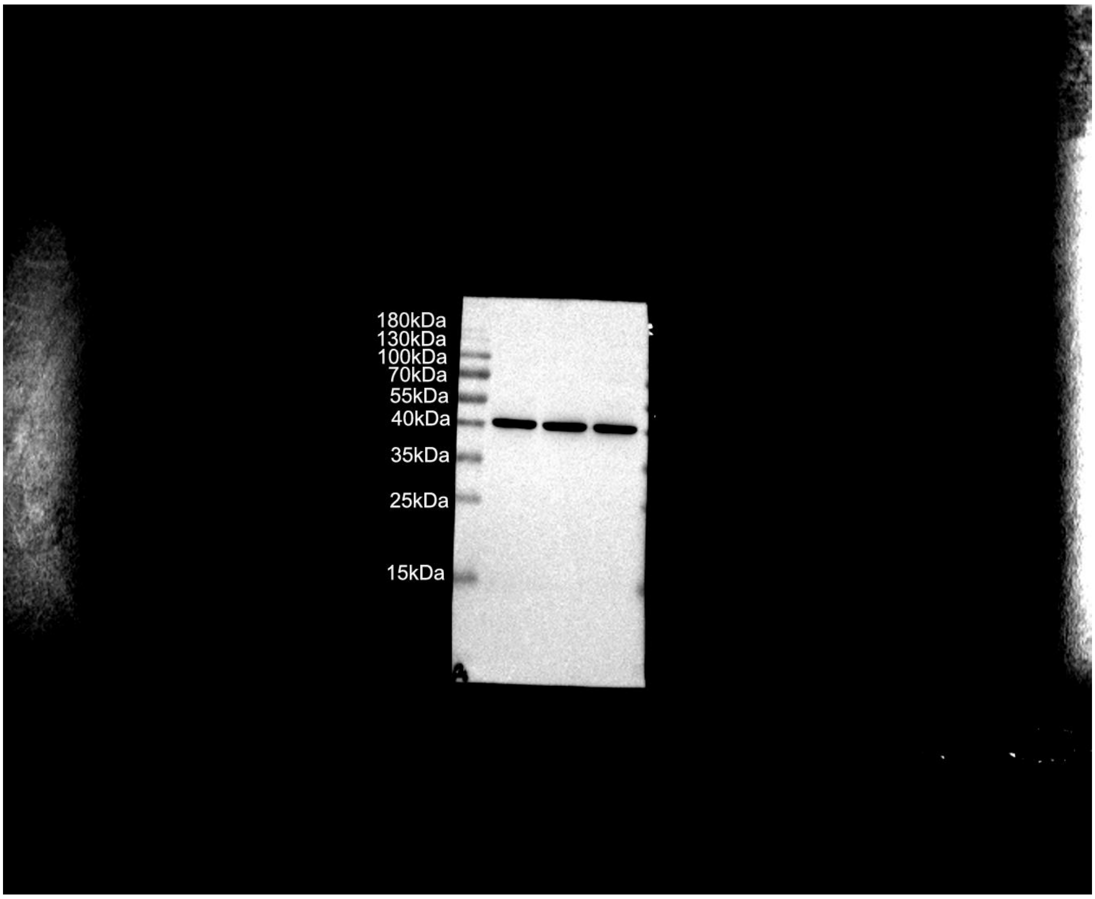

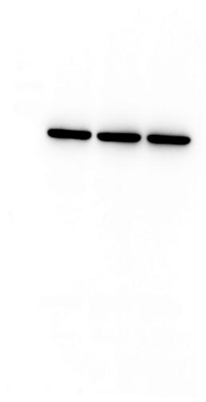

Fig5D HCT116 oe-RFC2

p-PI3K

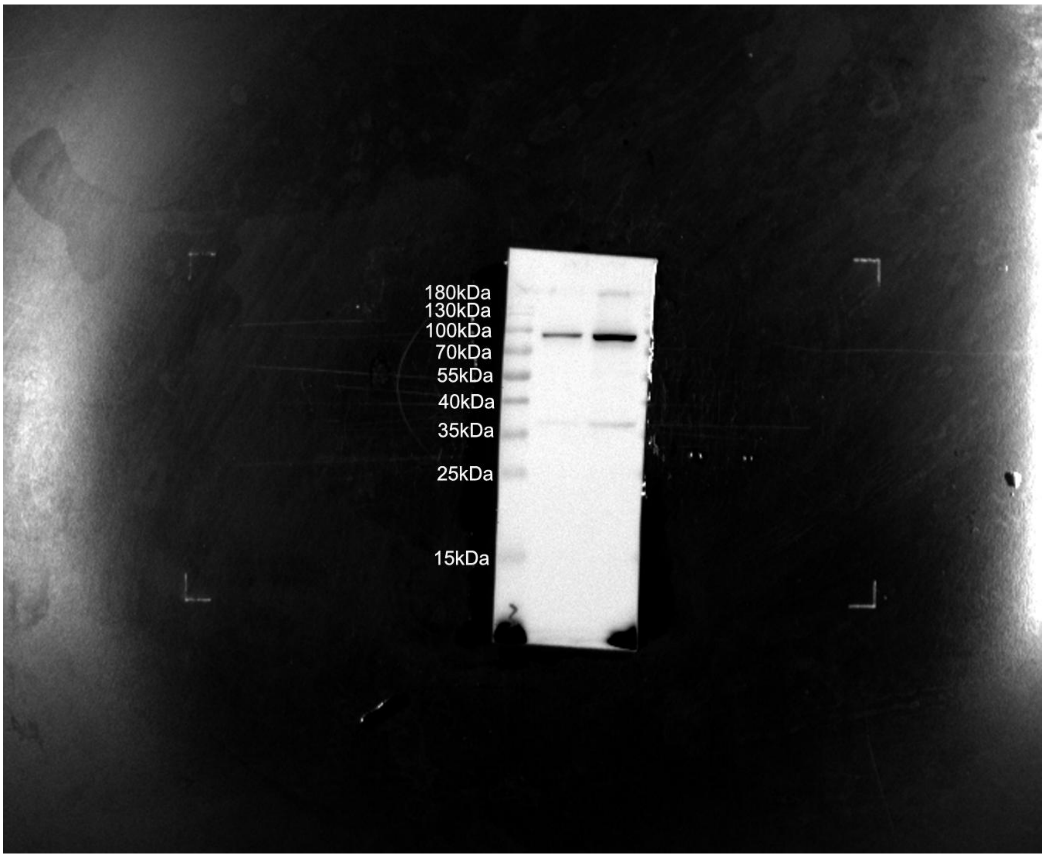

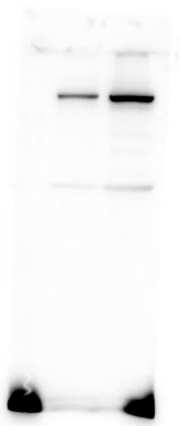

PI3K

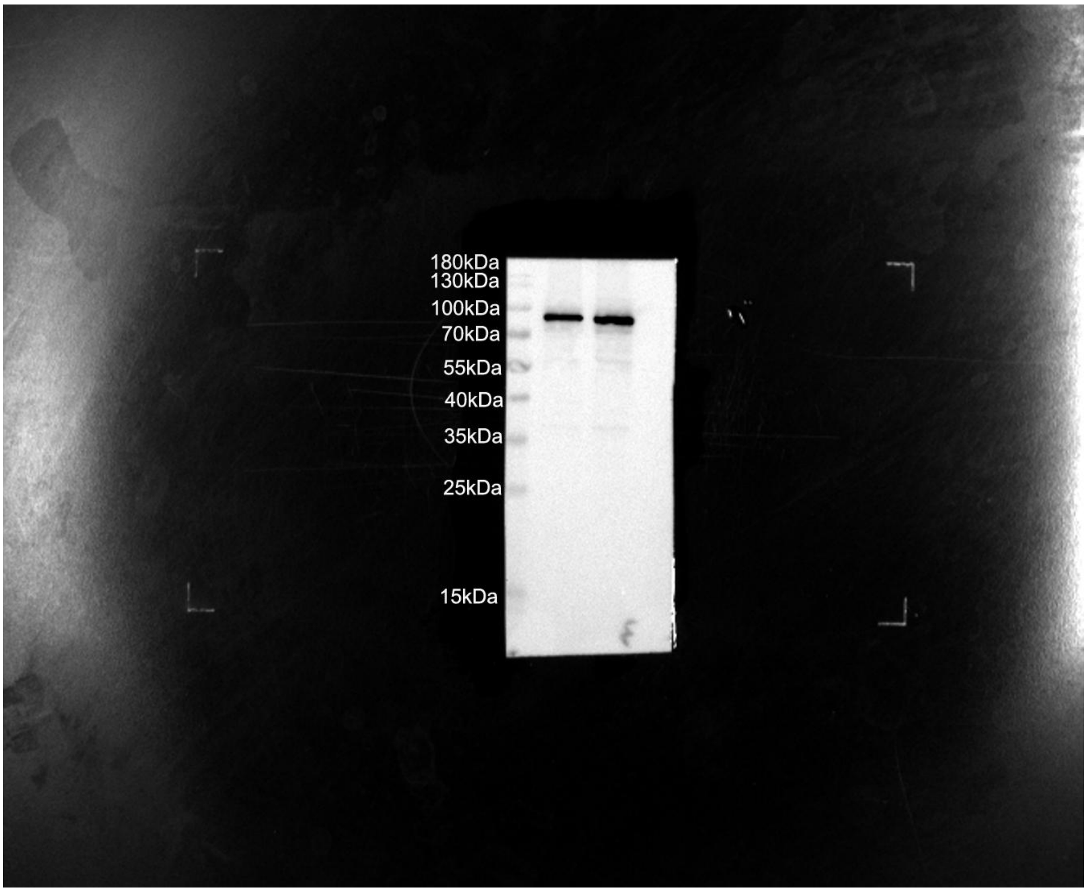

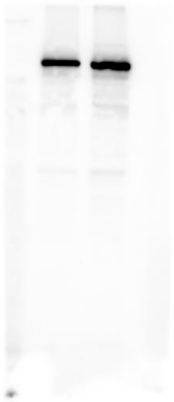

p-AKT

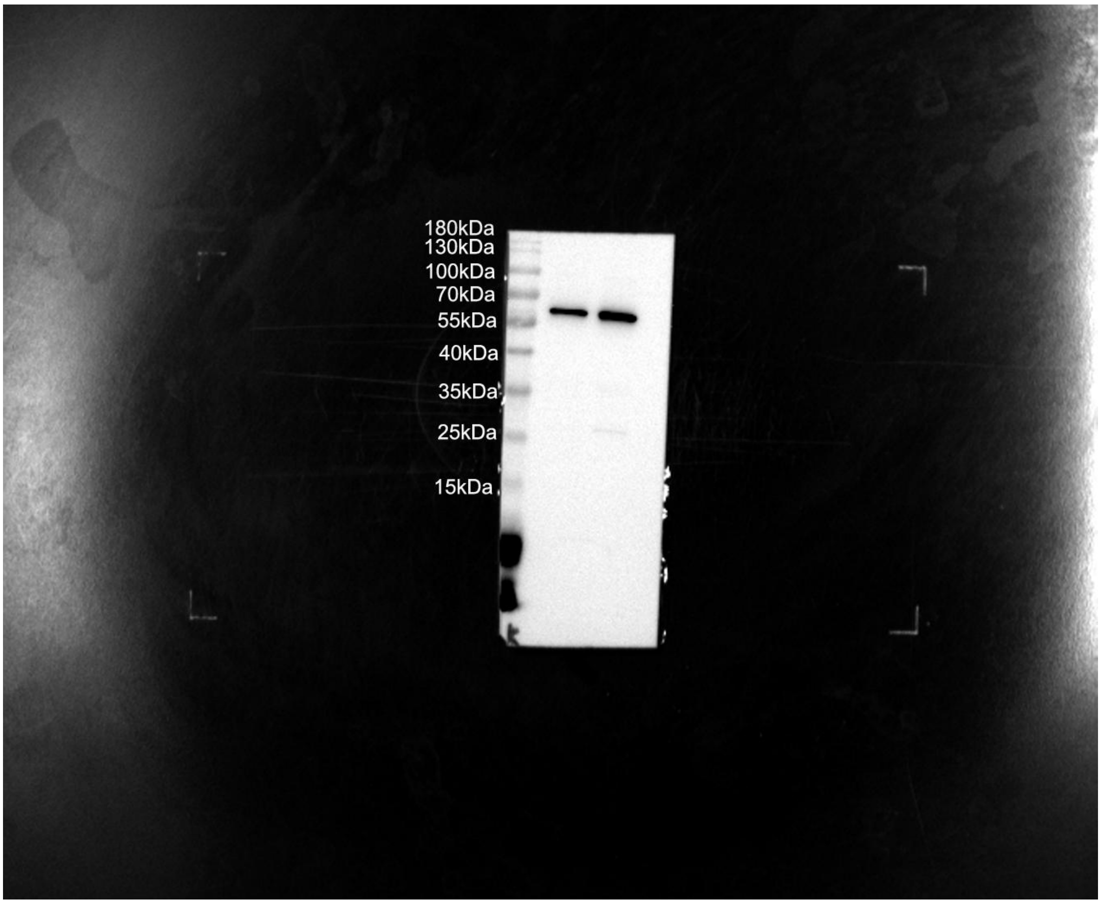

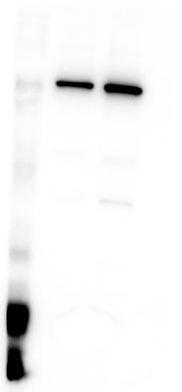

AKT

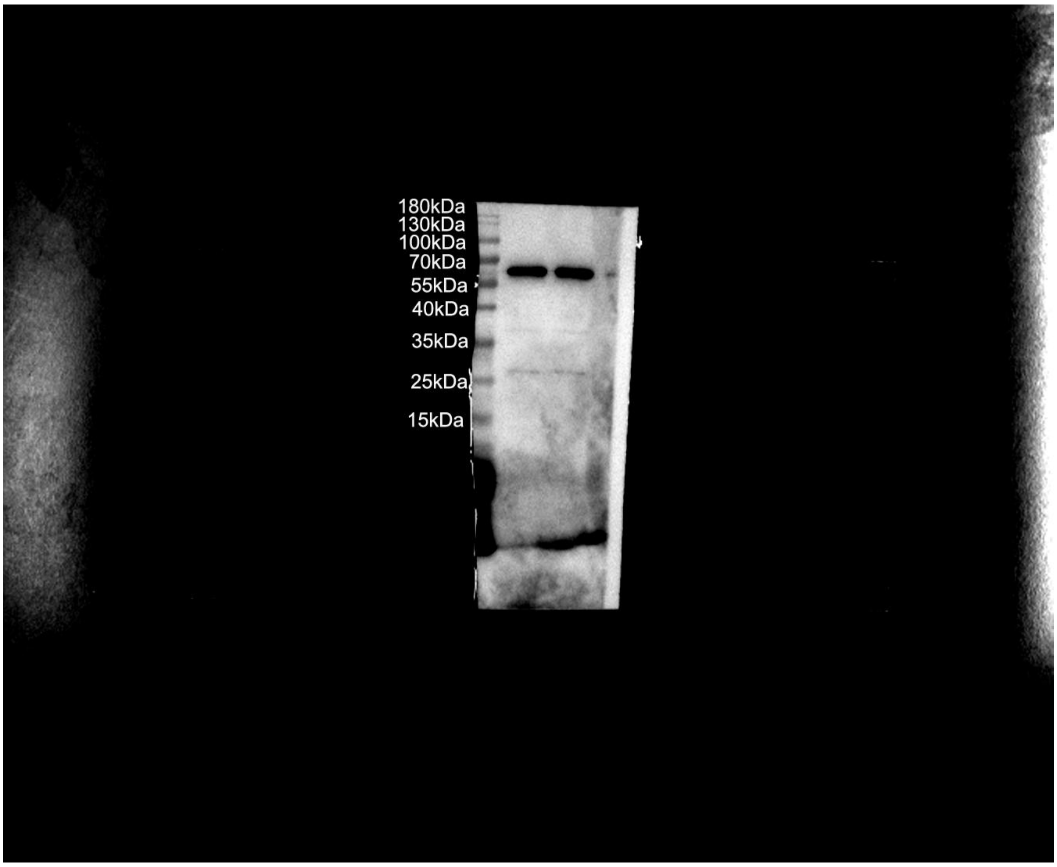

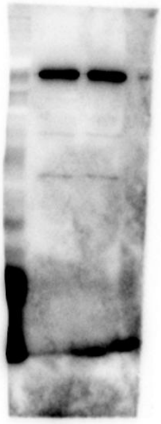

p-mTOR

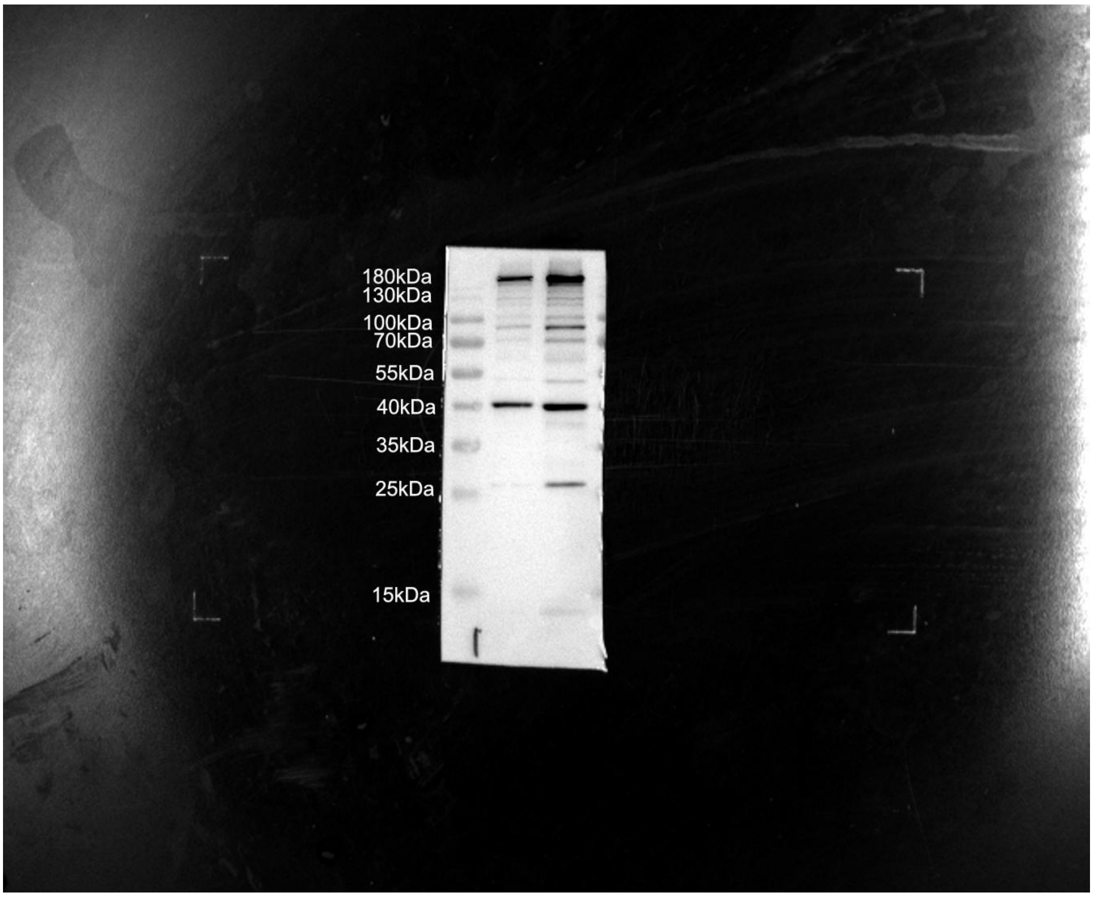

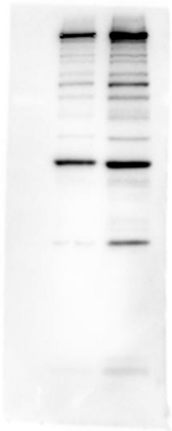

mTOR

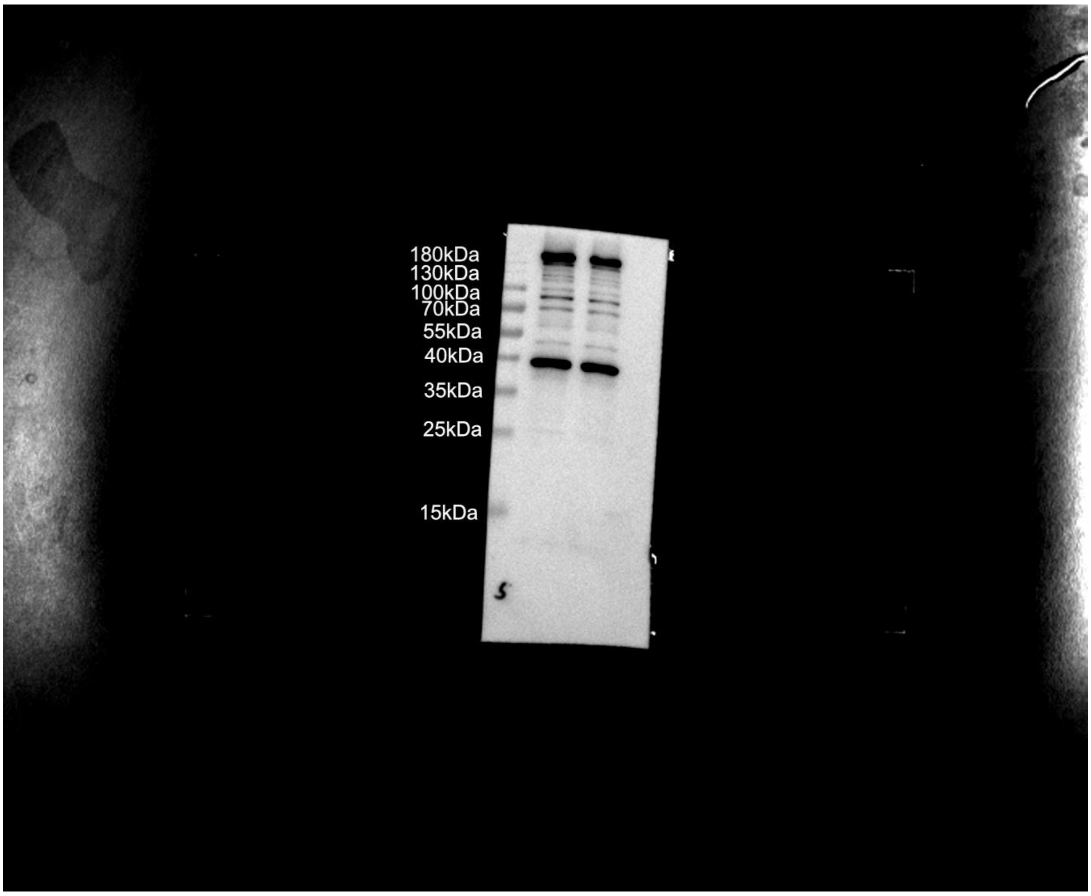

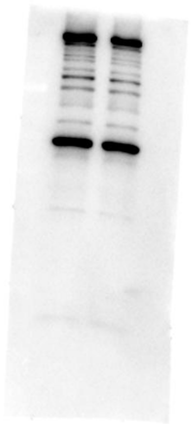

p-70S6K

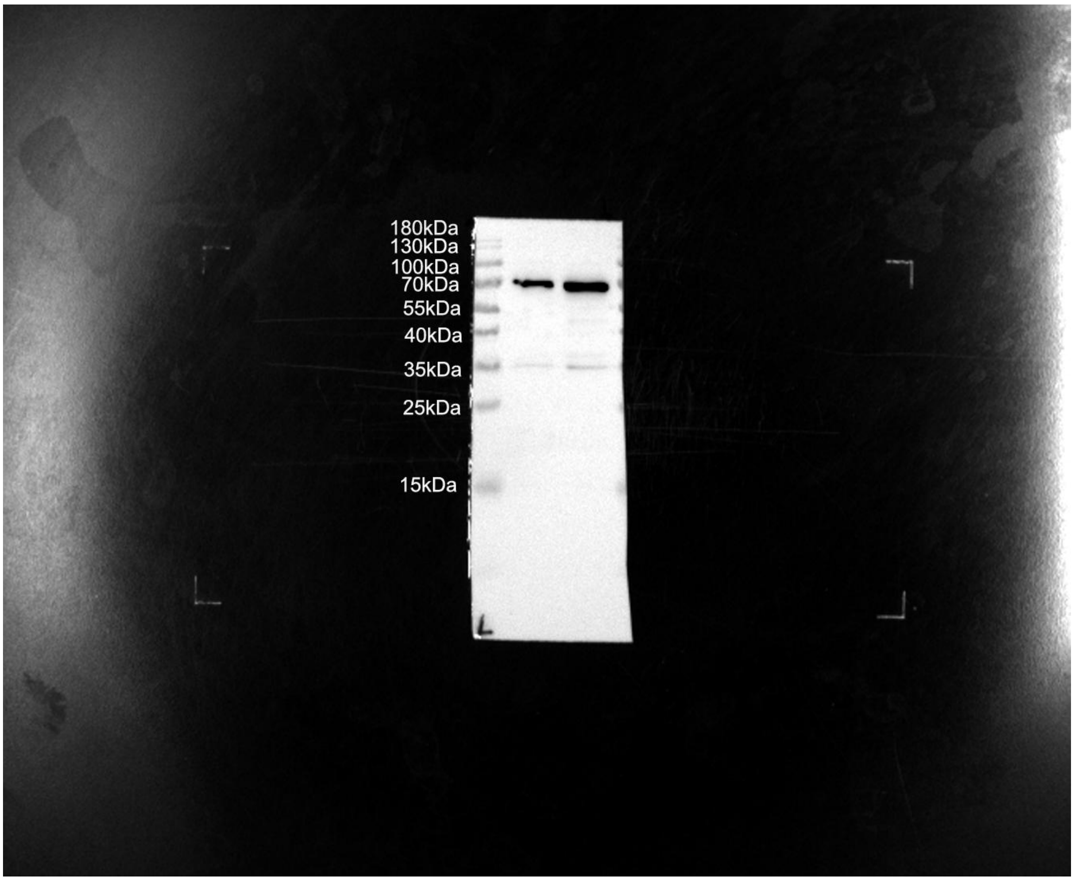

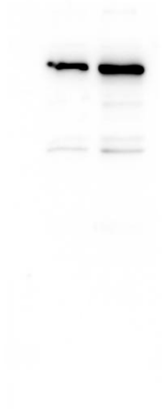

70S6K

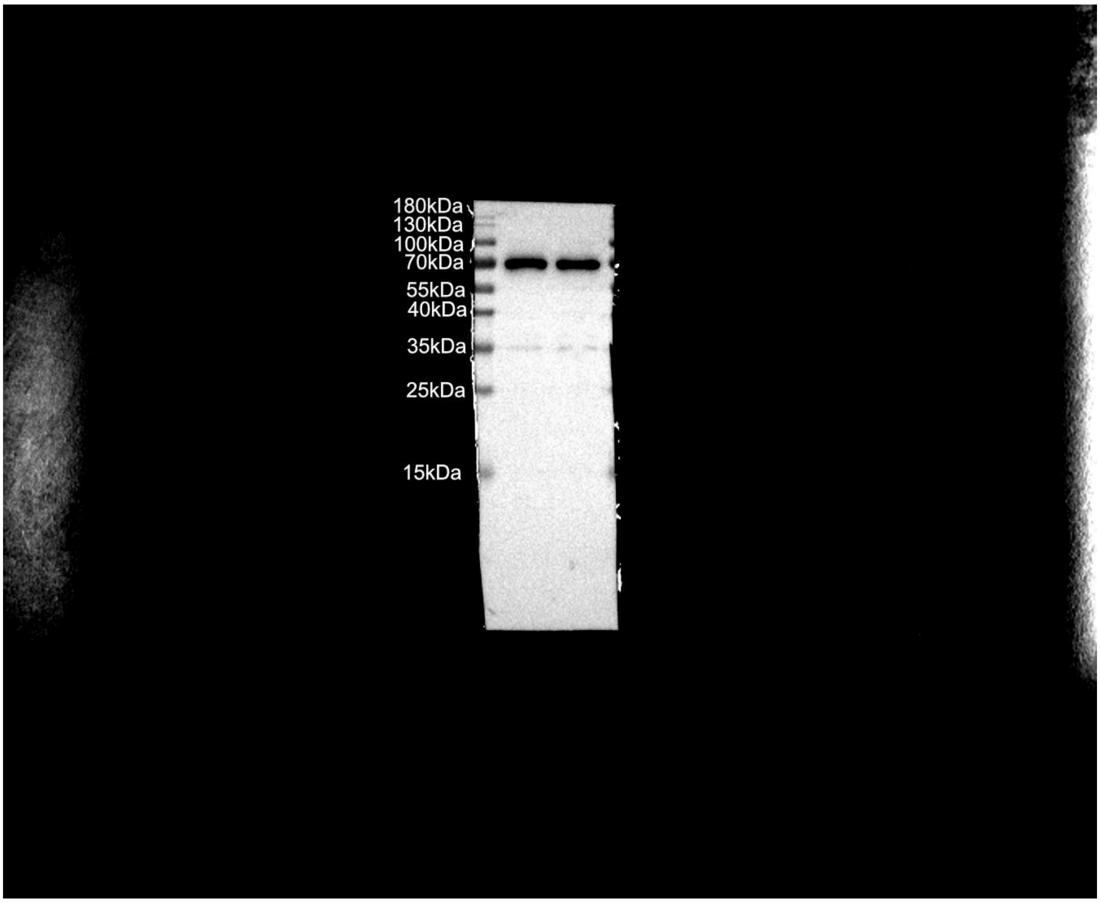

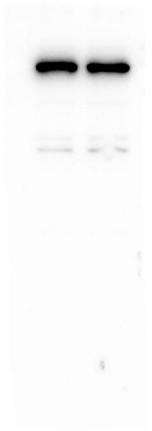

β-actin

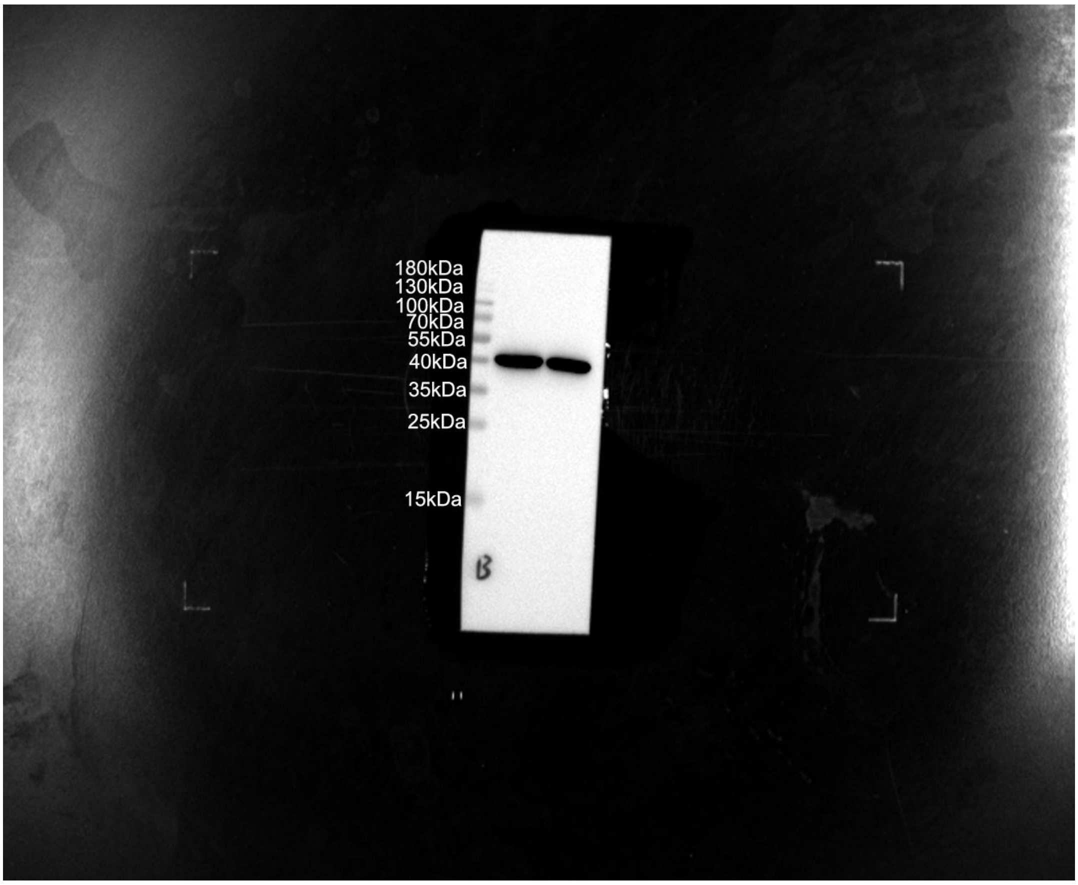

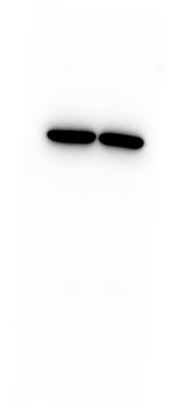

Fig5D SW480 oe-RFC2

p-PI3K

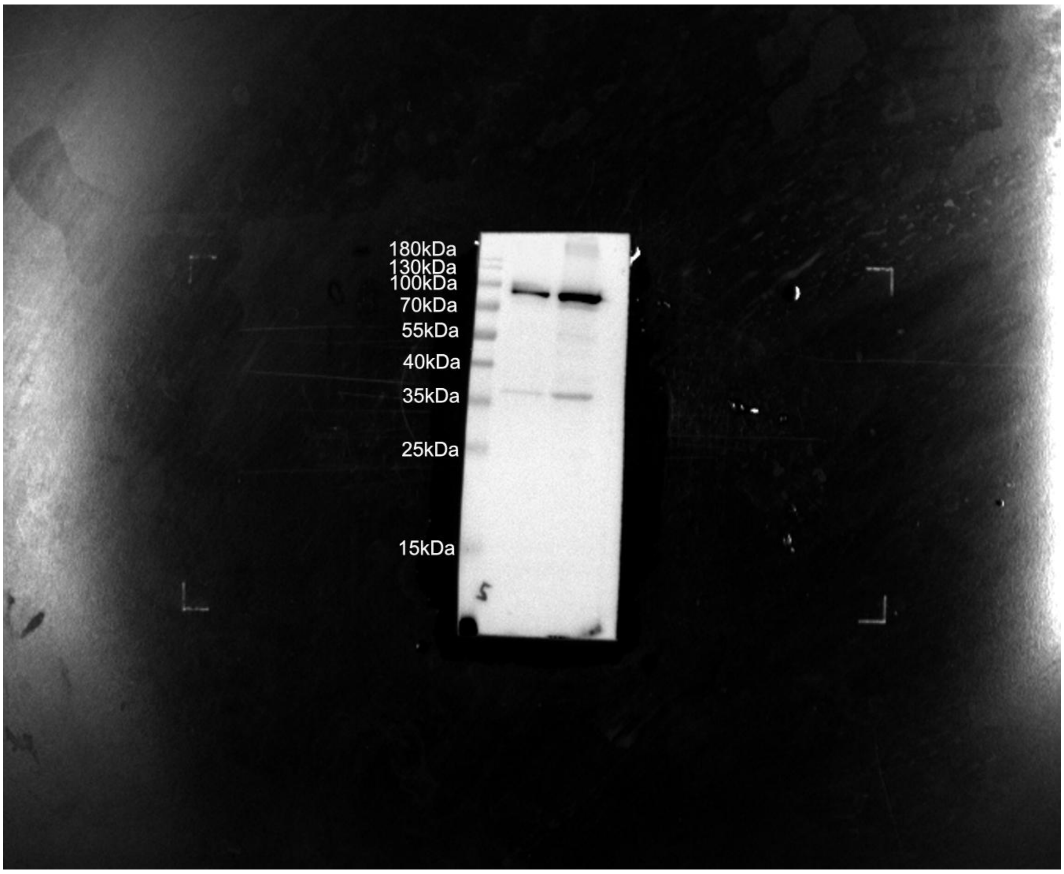

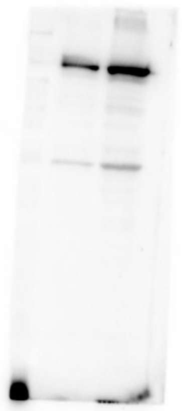

PI3K

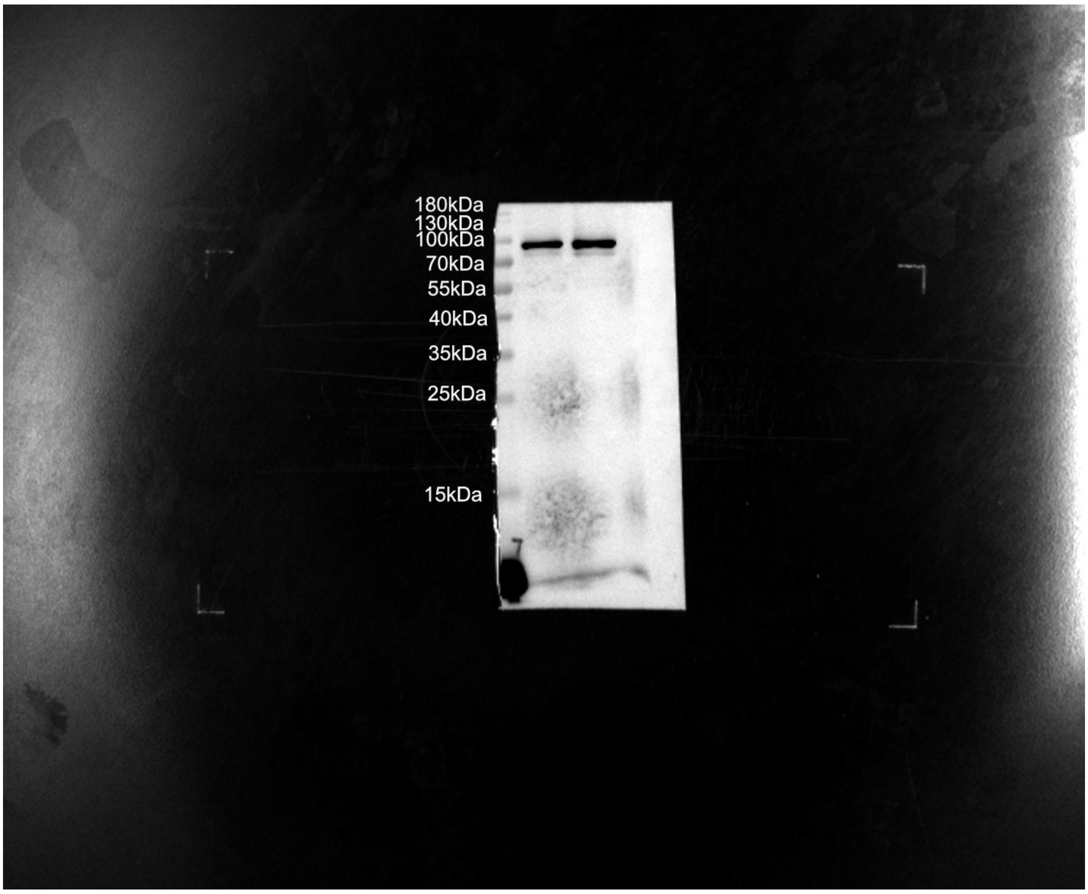

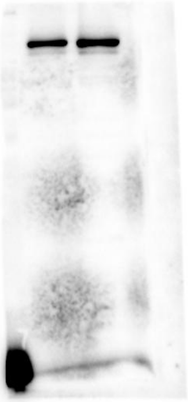

p-AKT

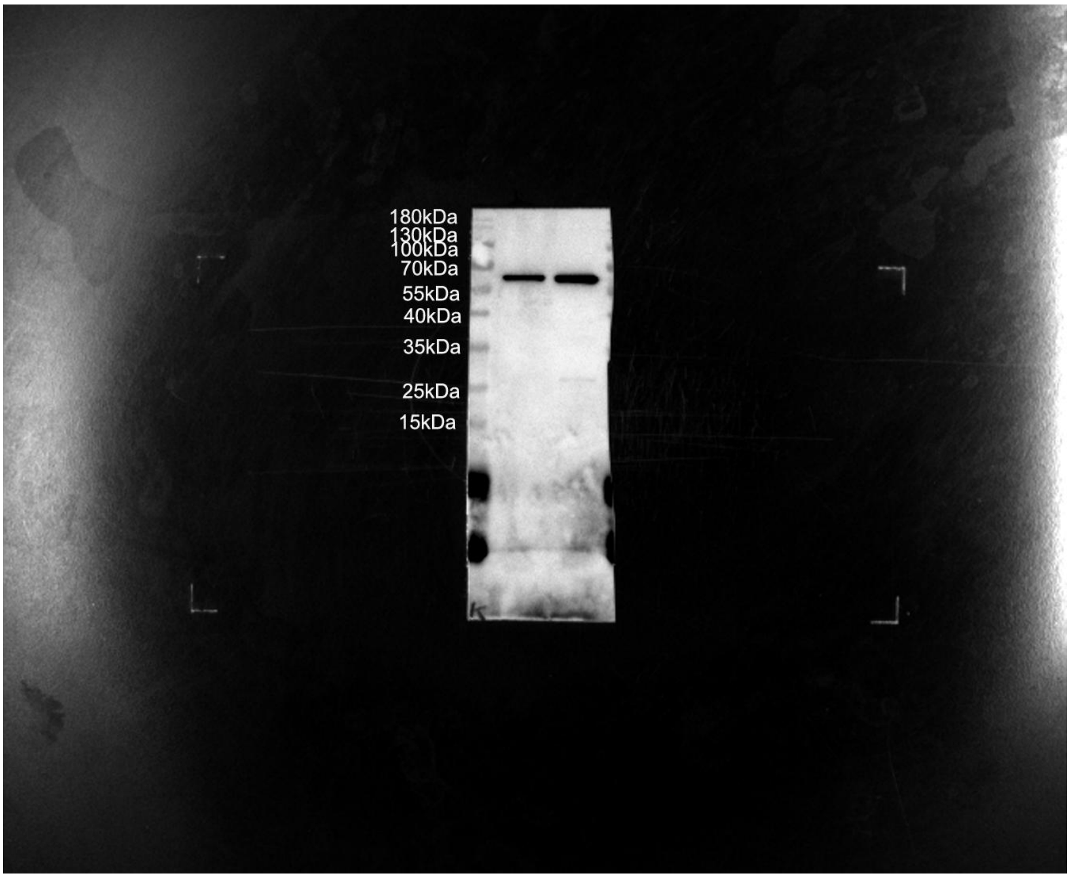

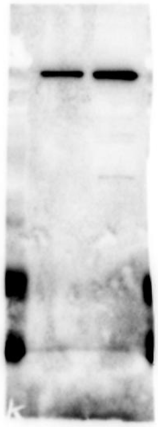

AKT

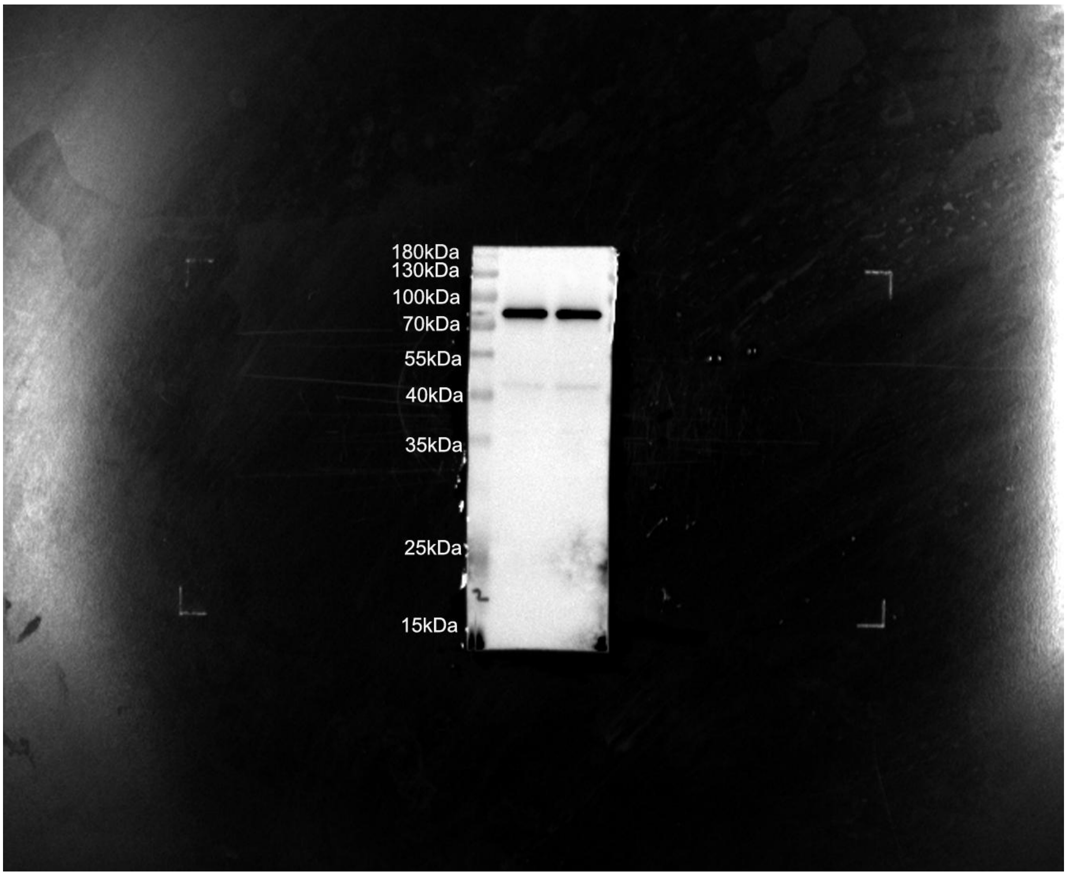

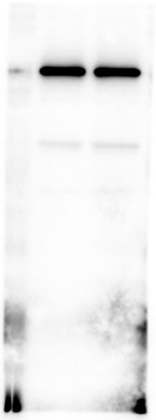

p-mTOR

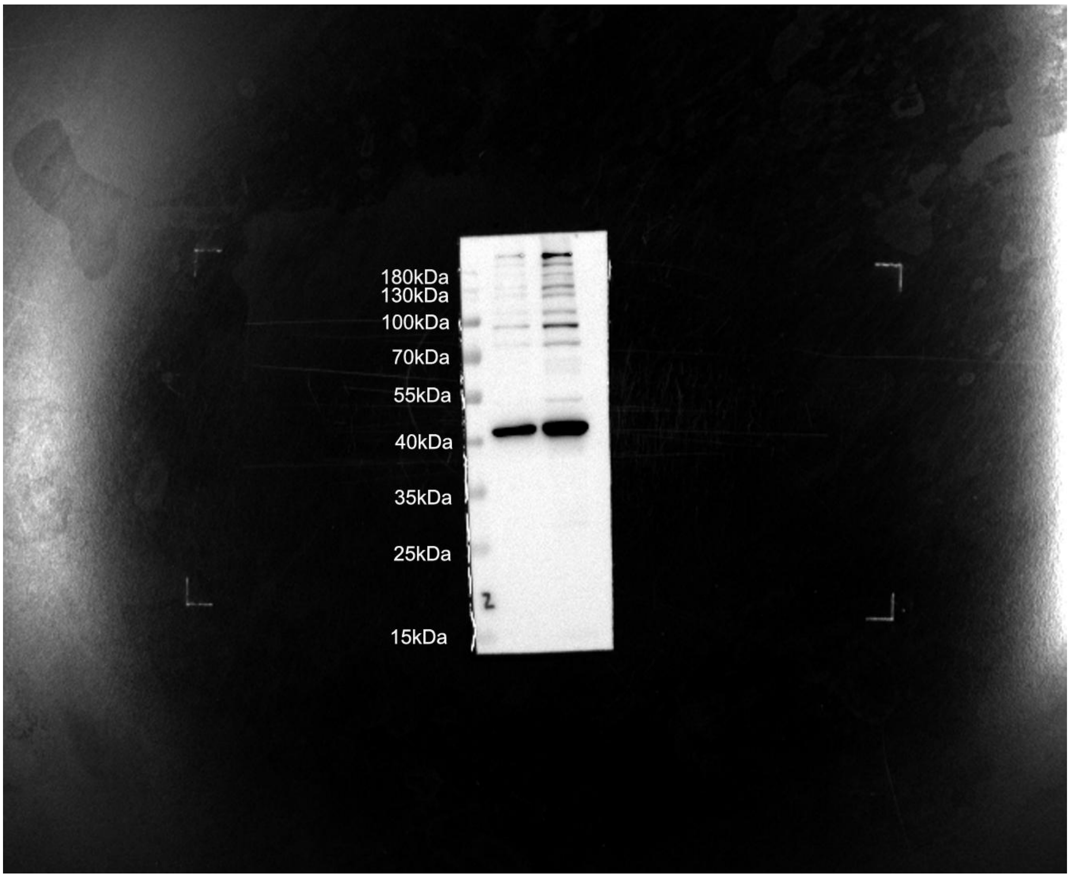

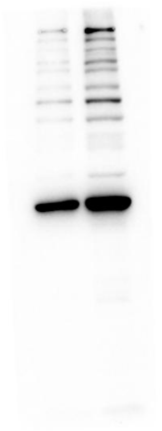

mTOR

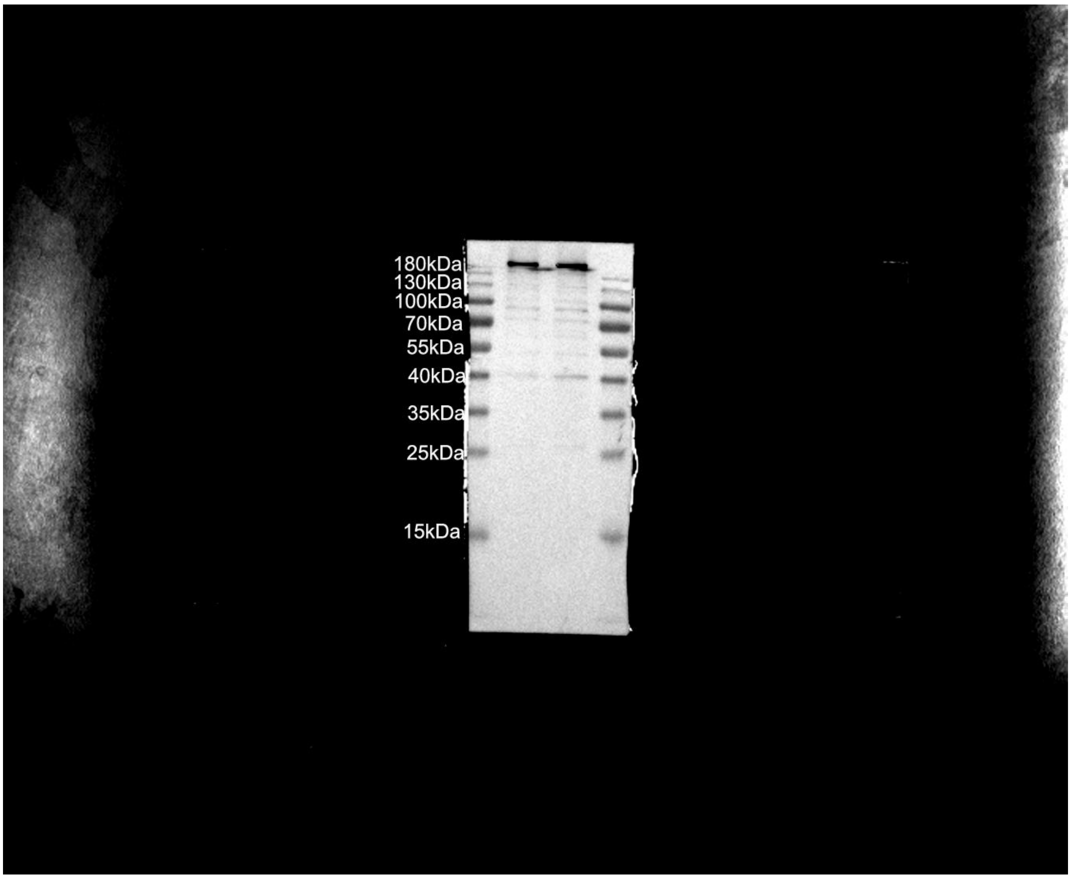

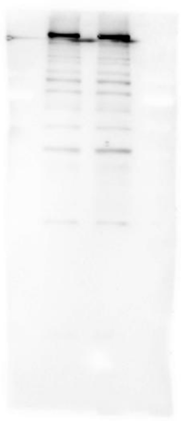

p-70S6K

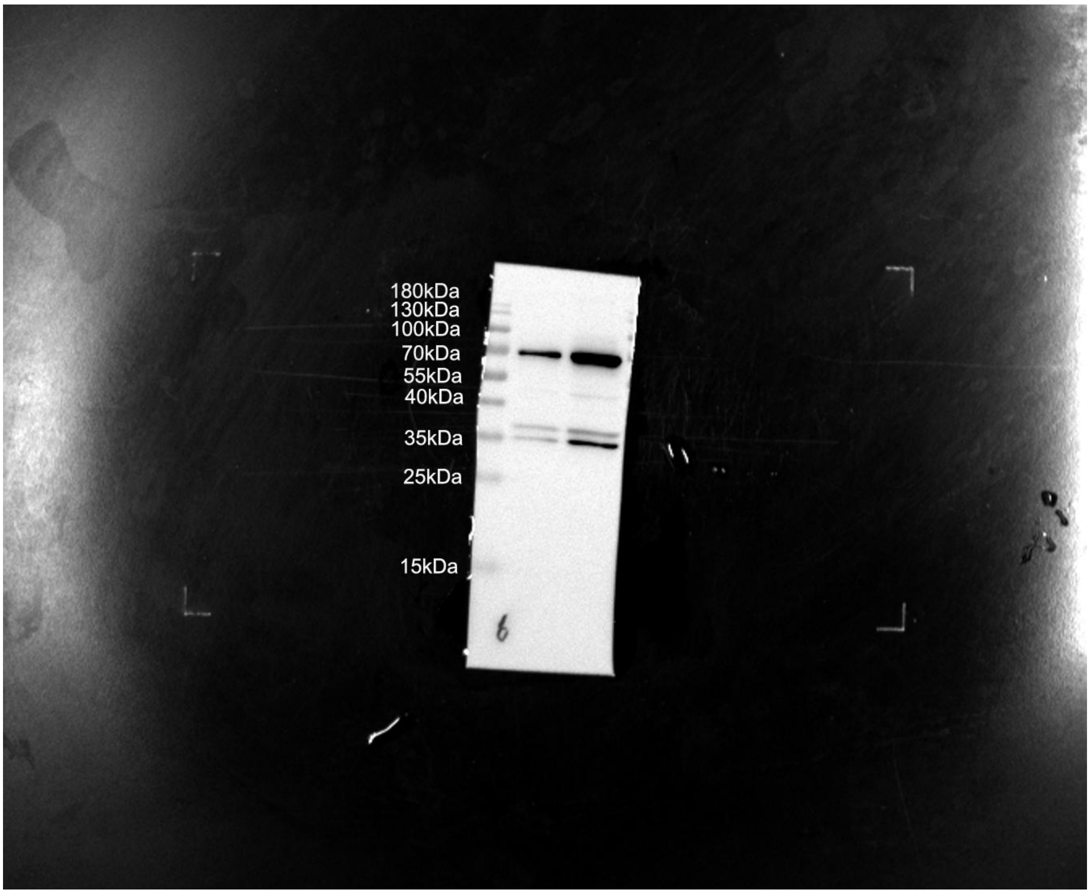

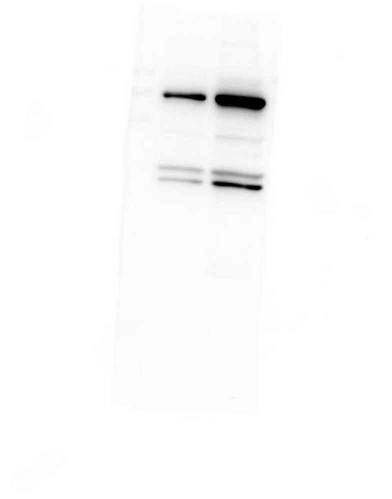

70S6K

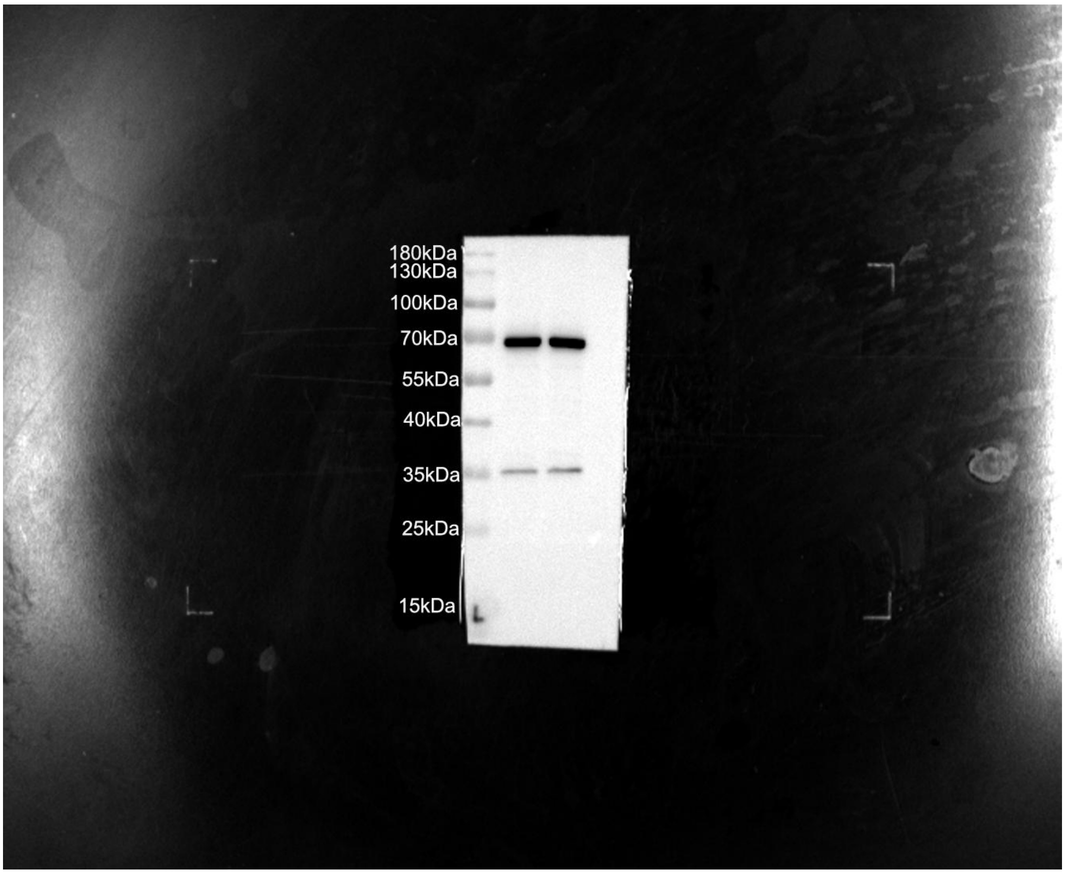

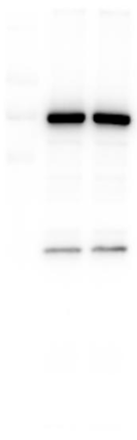

β-actin

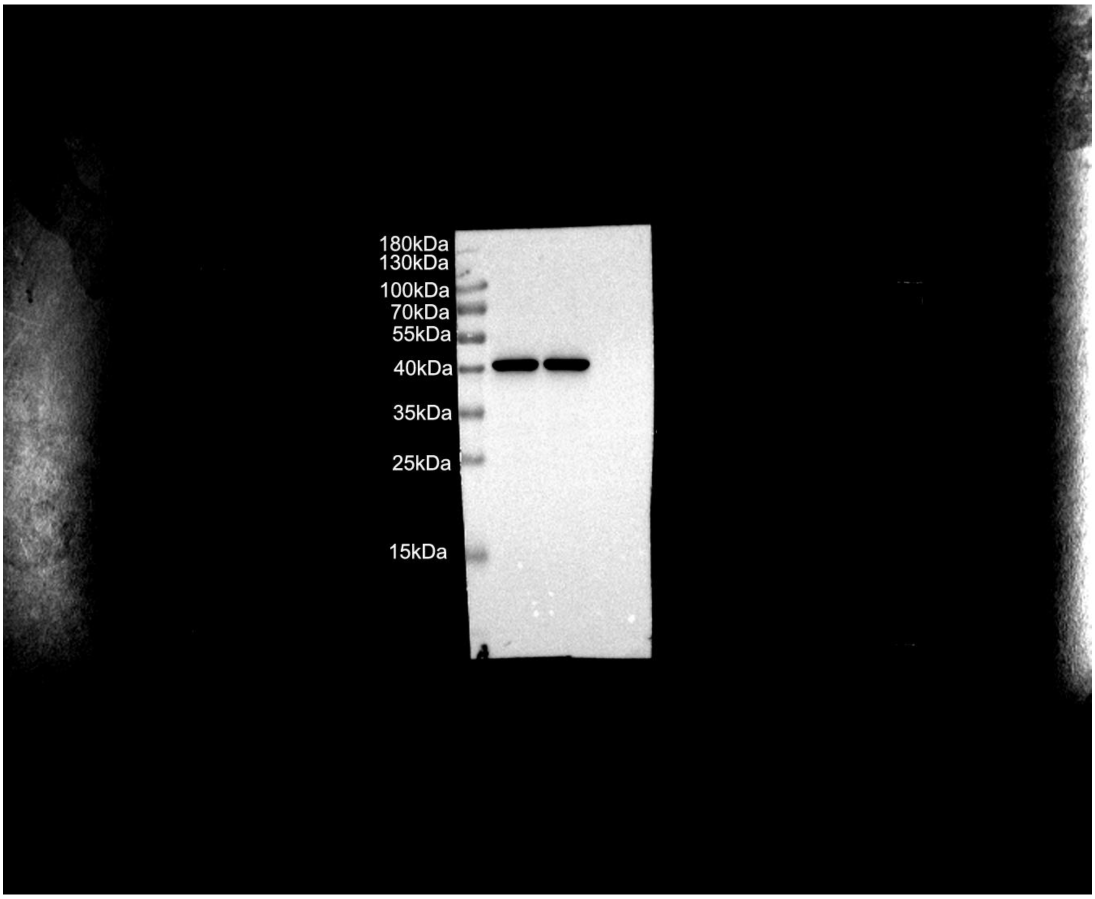

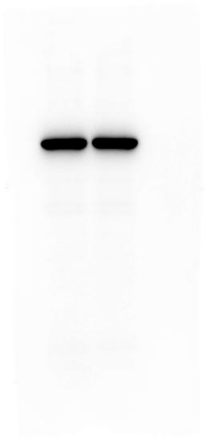

Fig6F si-RFC2

LDHA

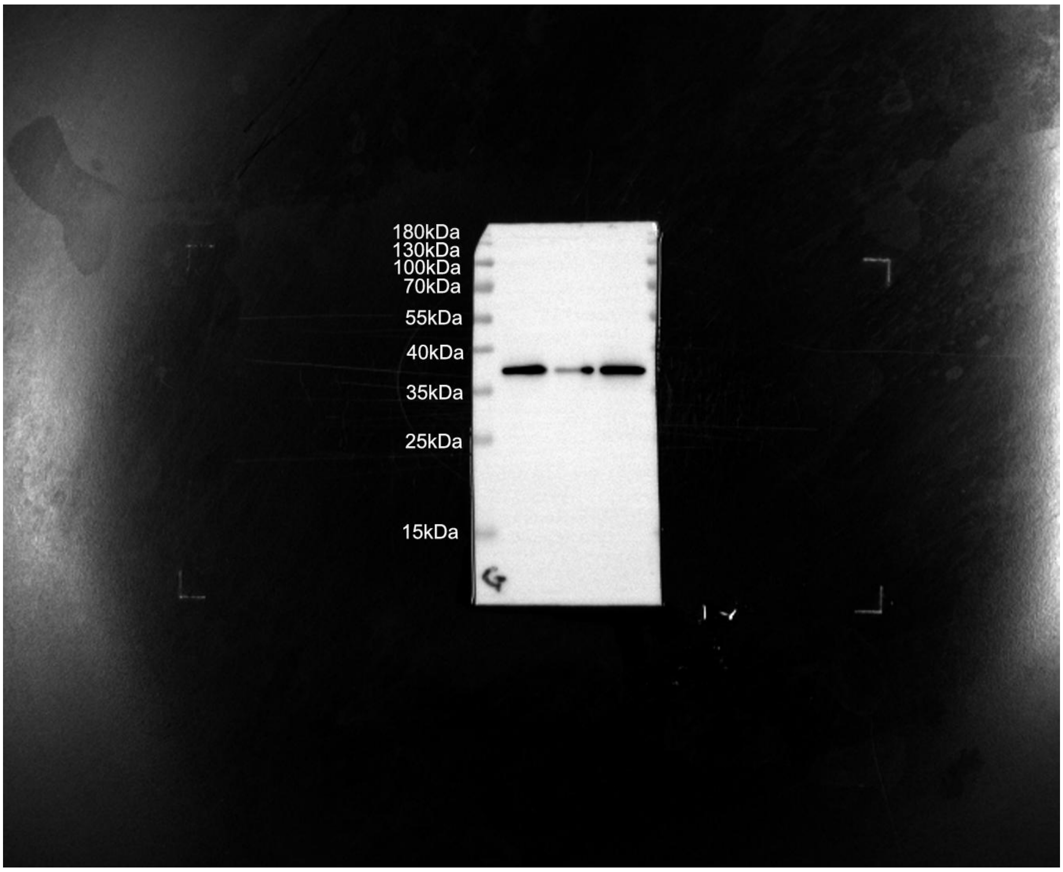

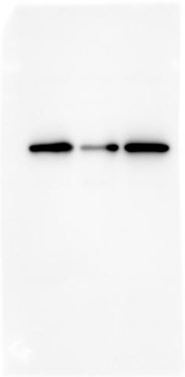

GLUT1

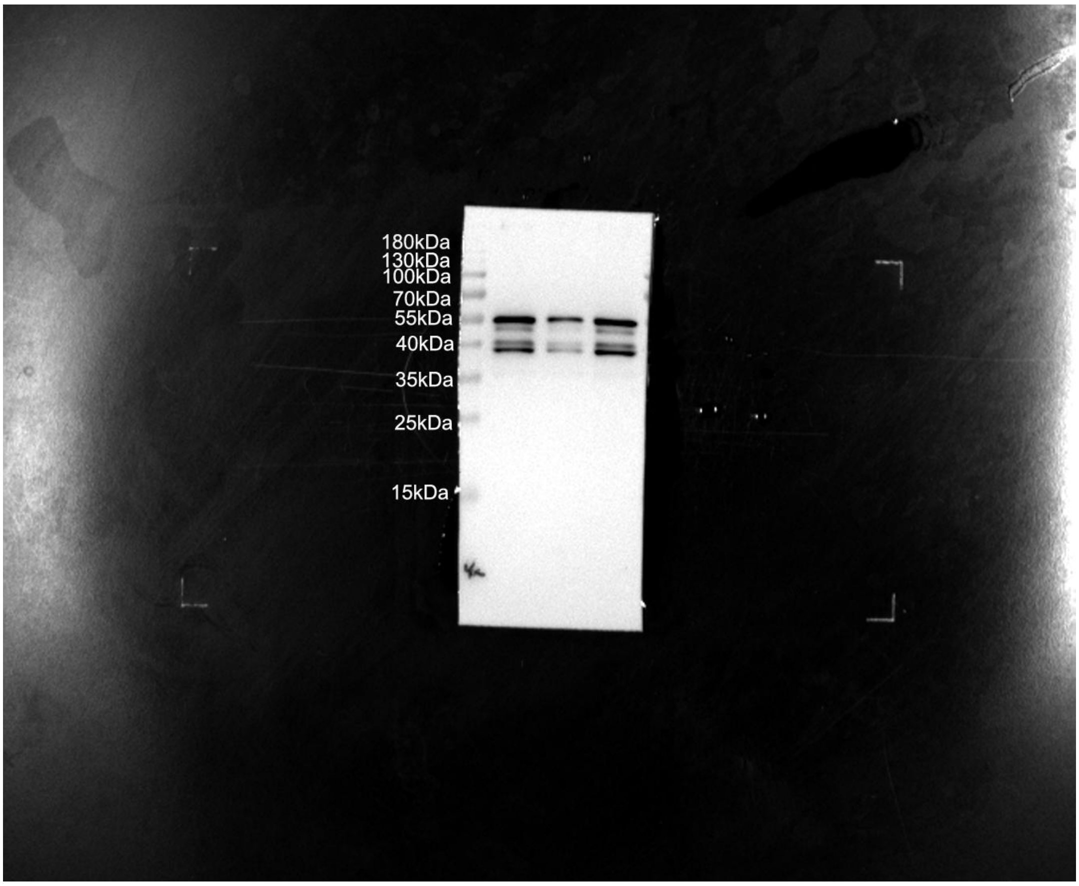

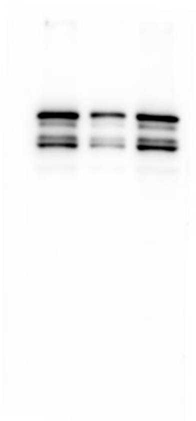

HK2

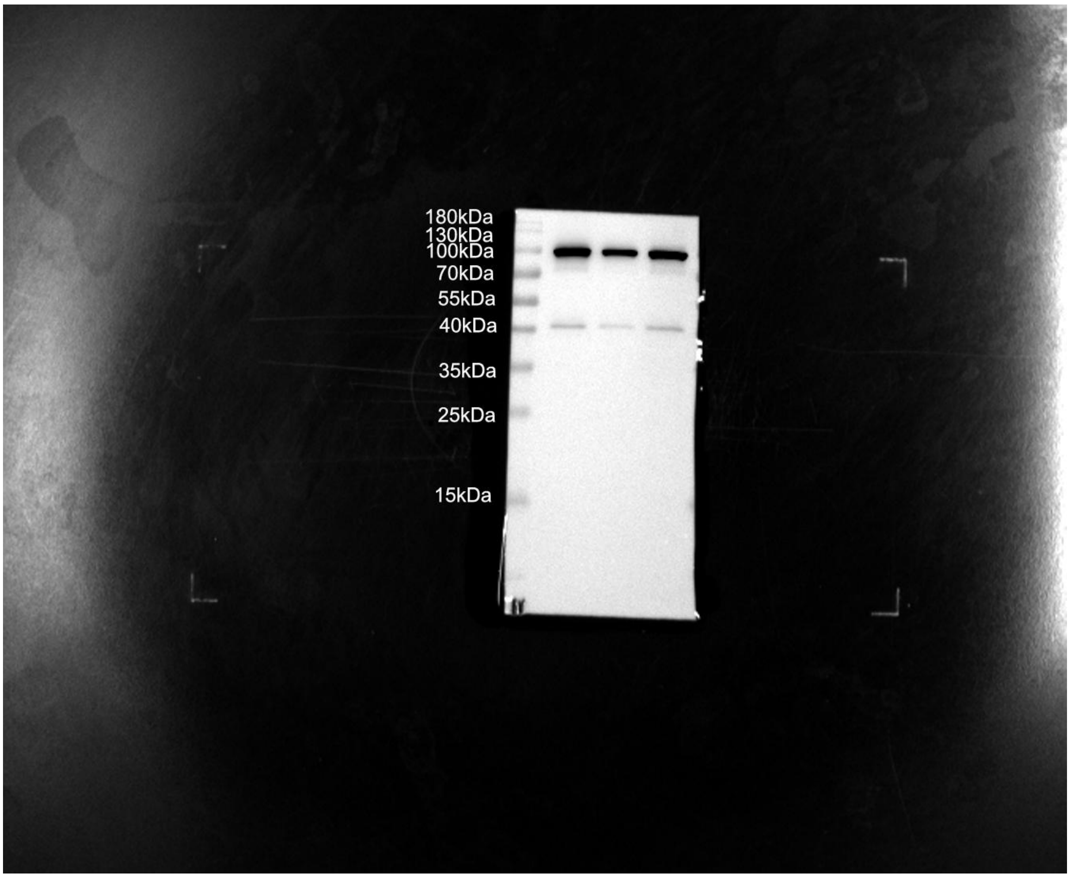

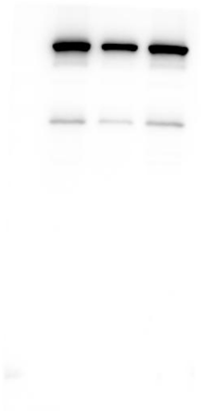

β-actin

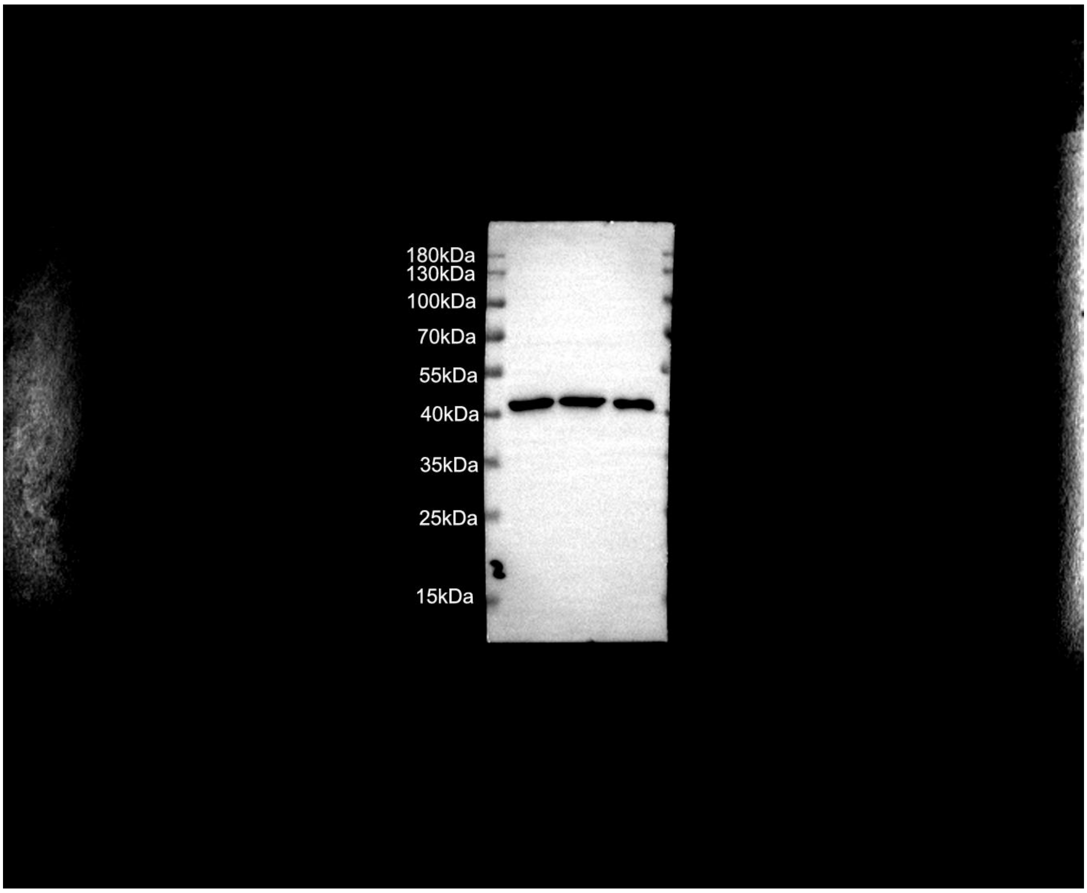

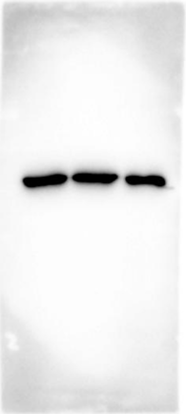

Fig6F oe-RFC2

LDHA

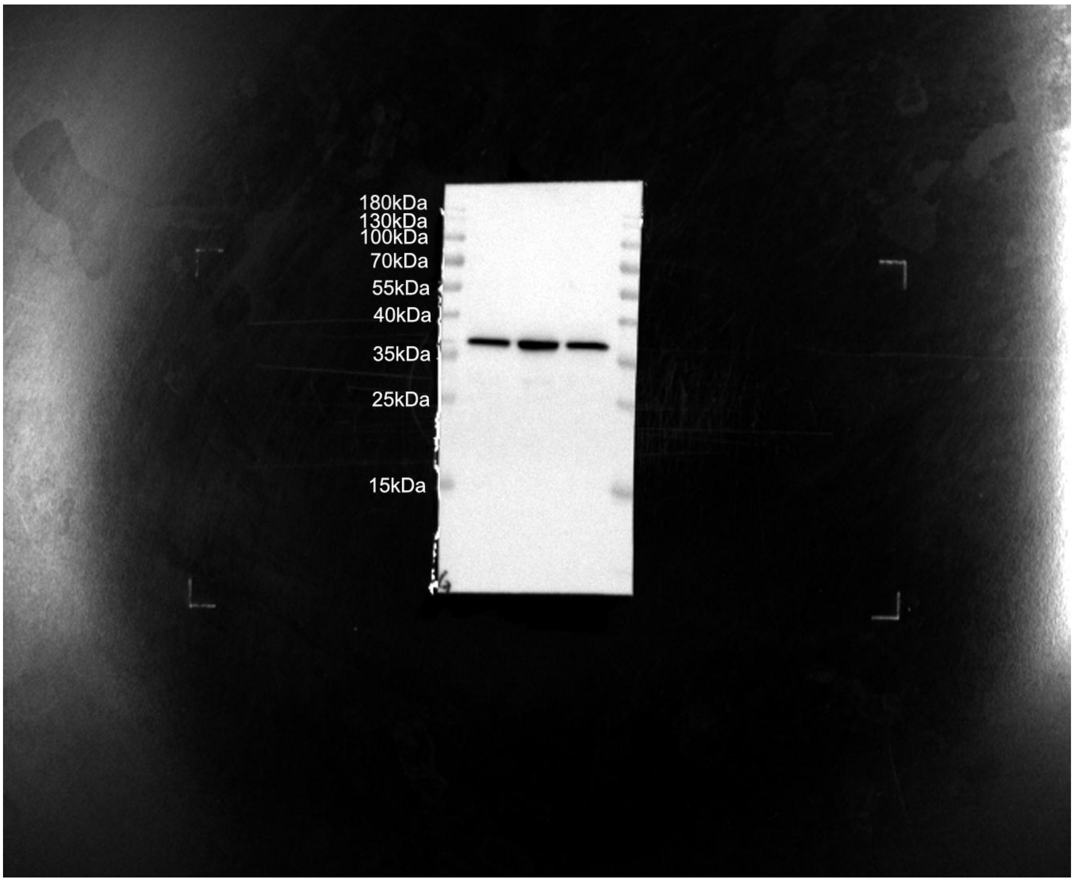

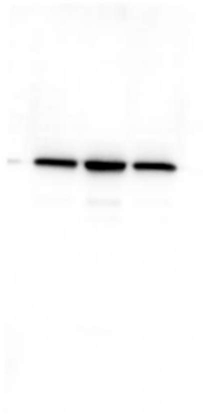

GLUT1

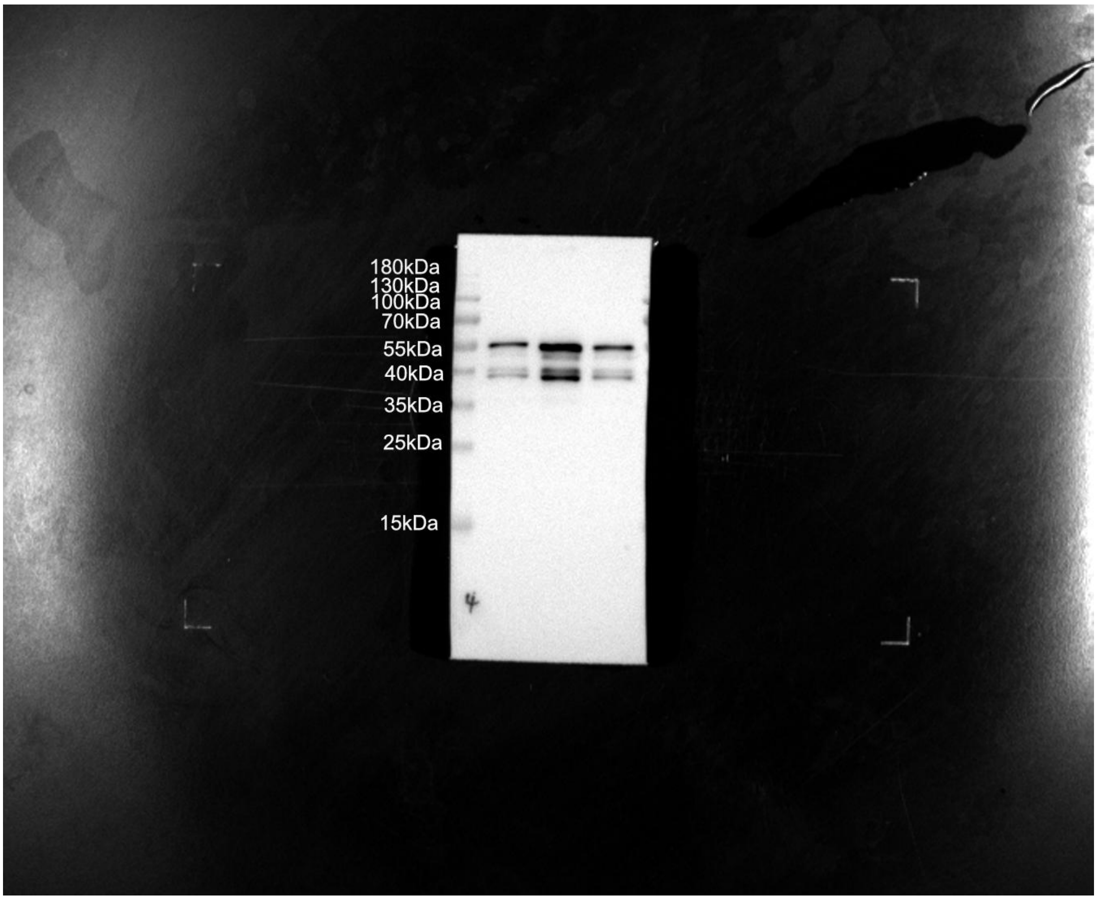

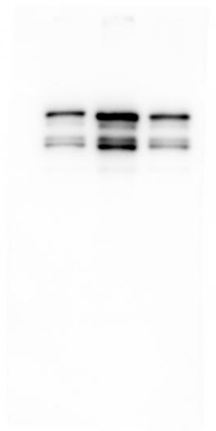

HK2

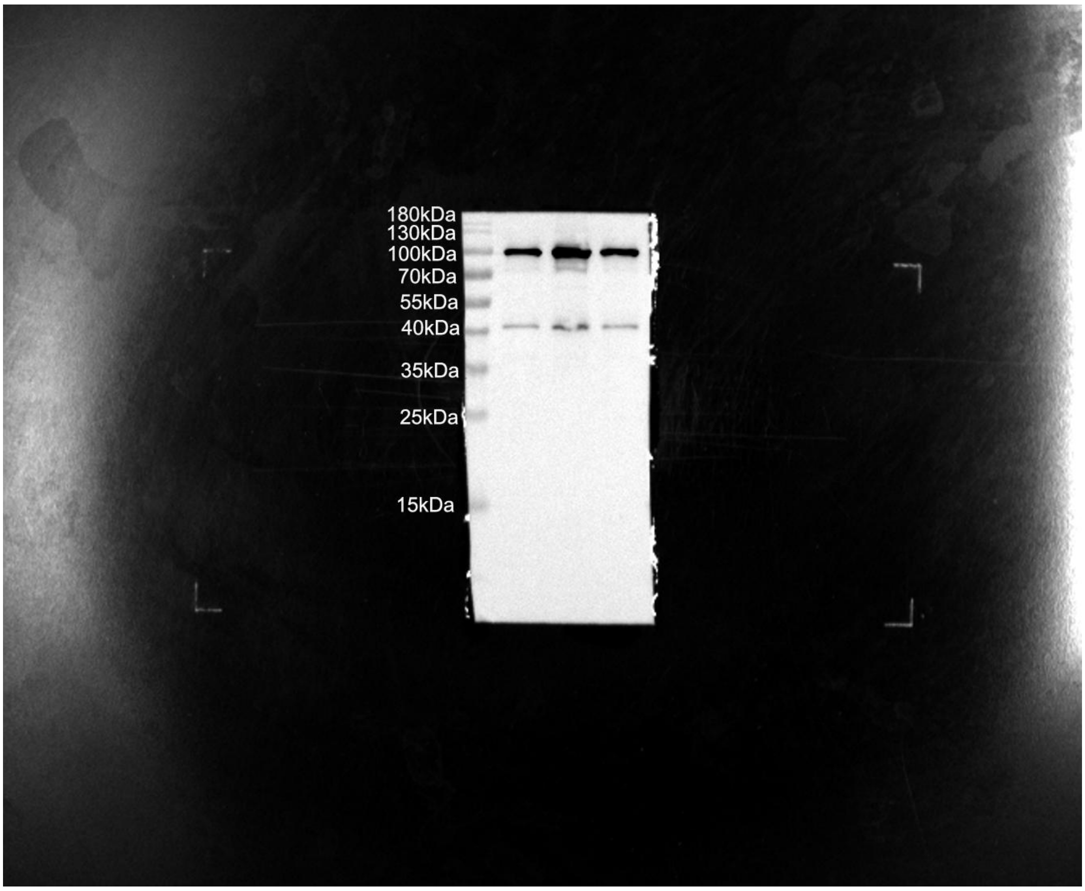

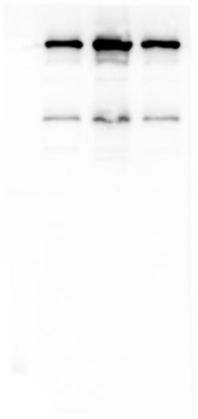

β-actin

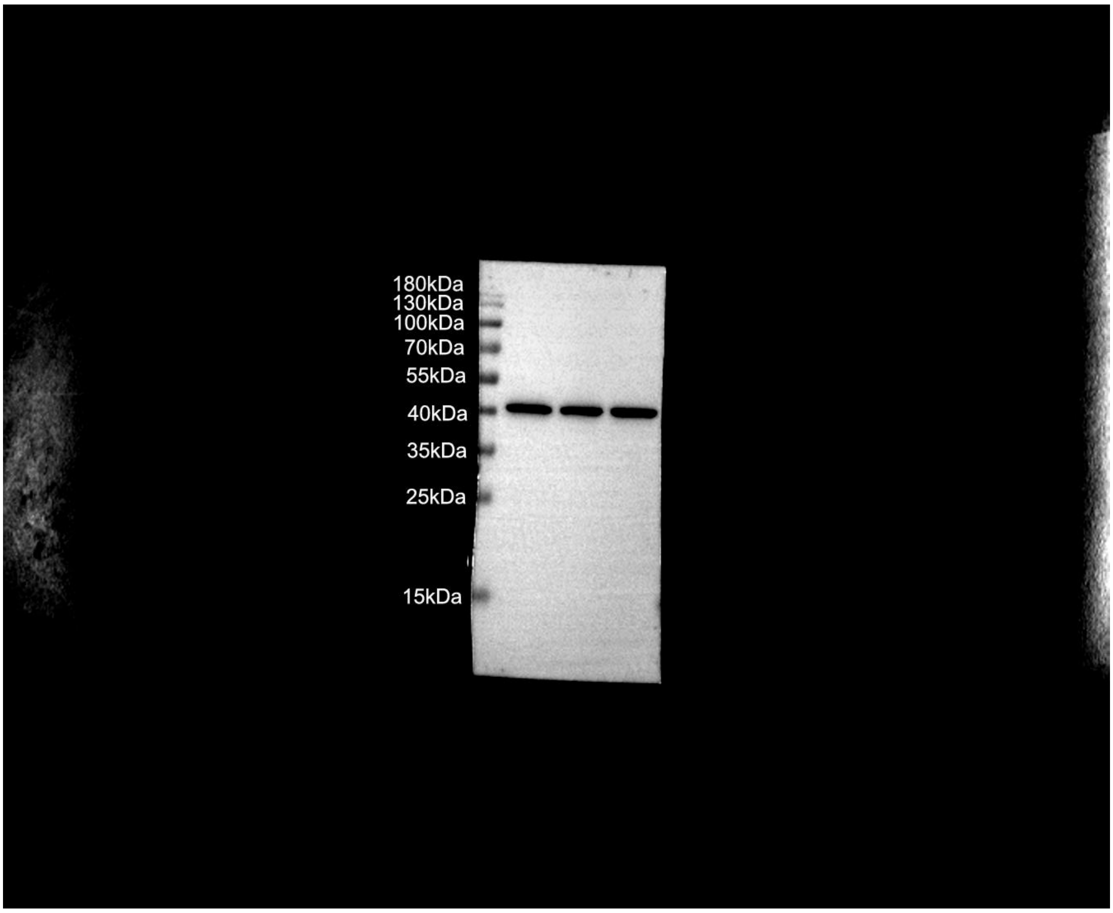

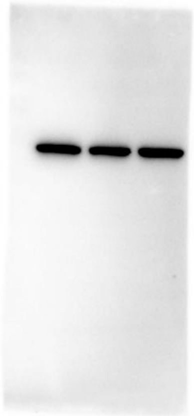

Supplement: Supplementary file 1 — Additional file 1. [file 12876_2023_2984_MOESM1_ESM.pdf]
